# *TREML4* Promotes Inflammatory Programs in Human and Murine Macrophages and Alters Atherosclerosis Lesion Composition in the Apolipoprotein E Deficient Mouse

**DOI:** 10.3389/fimmu.2020.00397

**Published:** 2020-03-30

**Authors:** Marieli Gonzalez-Cotto, Liang Guo, Megan Karwan, Shurjo K. Sen, Jennifer Barb, Carlos J. Collado, Fathi Elloumi, Erika M. Palmieri, Kimberly Boelte, Frank D. Kolodgie, Aloke V. Finn, Leslie G. Biesecker, Daniel W. McVicar

**Affiliations:** ^1^Cancer and Inflammation Program, National Cancer Institute, NIH, Frederick, MD, United States; ^2^CVPath Institute, Gaithersburg, MD, United States; ^3^Laboratory Animal Sciences Program, Leidos Biomedical Research Inc., Cancer and Inflammation Program, Frederick National Laboratory for Cancer Research, Frederick, MD, United States; ^4^Mathematical and Statistical Computing Laboratory, Center for Information Technology (CIT), NIH, Bethesda, MD, United States; ^5^Center for Cancer Research Collaborative Bioinformatics Resource, Leidos Biomedical Research, Inc., Bethesda, MD, United States; ^6^Medical Genomics and Metabolic Genetics Branch, National Human Genome Research Institute, NIH, Bethesda, MD, United States

**Keywords:** treml4, inflammation, atherosclerosis, macrophages, TREM family

## Abstract

The Triggering Receptor Expressed on Myeloid cells-like 4 (TREML4) is a member of the TREM receptor family, known modulators of inflammatory responses. We have previously found that *TREML4* expression positively correlates with human coronary arterial calcification (CAC). However, the role of *TREML4* in the pathogenesis of cardiovascular disease remains incompletely defined. Since macrophages play a key role in inflammatory conditions, we investigated if activated macrophages selectively expressed *TREML4* and found that carriage of either one of the eQTL SNP’s previously associated with increased *TREML4* expression conferred higher expression in human inflammatory macrophages (M1) compared to alternatively activated macrophages (M2). Furthermore, we found that *TREML4* expression in human M1 dysregulated several inflammatory pathways related to leukocyte activation, apoptosis and extracellular matrix degradation. Similarly, murine M1 expressed substantial levels of *Treml4*, as did oxLDL treated macrophages. Transcriptome analysis confirmed that murine *Treml4* controls the expression of genes related to inflammation and lipid regulation pathways, suggesting a possible role in atherosclerosis. Analysis of *Apoe^–/–^/Treml4^–/–^* mice showed reduced plaque burden and lesion complexity as indicated by decreased stage scores, macrophage content and collagen deposition. Finally, transcriptome analysis of oxLDL-loaded murine macrophages showed that *Treml4* represses a specific set of genes related to carbohydrate, ion and amino acid membrane transport. Metabolomic analysis confirmed that *Treml4* deficiency may promote a beneficial relationship between iron homeostasis and glucose metabolism. Together, our results suggest that *Treml4* plays a role in the development of cardiovascular disease, as indicated by *Treml4*-dependent dysregulation of macrophage inflammatory pathways, macrophage metabolism and promotion of vulnerability features in advanced lesions.

## Introduction

Atherosclerosis is a progressive and dynamic disease characterized by low grade inflammation and arterial lipid deposition. Great advances have been made regarding the characterization of disease, especially in defining progression stages (1). Human atherosclerosis can now be practically divided into several stages which has aided in characterizing critical modulators and risk factors (2, 3). There is, however, still a profound gap in knowledge regarding disease progression to complex lesions. Thus, the molecular mechanisms of atherosclerotic complications remain obscure, despite advances in modeling disease and unraveling factors in its progression, predominantly through the use of animal models.

Traditional GWAS studies and similar approaches have, thus far, met with limited success in identifying genes and pathways relevant to advanced atherosclerosis, leaving the underlying causes of disease complications largely elusive (4–8). Recently, by using an integrated multiomics and functional approach, our group identified expression of the Triggering Receptor Expressed on Myeloid cells (TREM)-like 4 (TREML4) to be strongly correlated with Coronary Arterial Calcification (CAC) (9). In patients with high CAC scores, *TREML4* expression was higher at both the transcript and protein levels. Furthermore, we identified two *cis-*eQTL SNP’s where the minor allele conferred up to 6.5-fold increased risk for CAC. Other groups have also identified dysregulated *TREML4* expression in patients with acute coronary syndrome (10), Alzheimer’s disease (11), post-stenotic collateral coronary artery disease (12) and coronary artery disease (13). However, while CAC and similar conditions are recognized as strong predictors for future cardiovascular events, it remains unclear whether they will predict plaque instability or simply correlate with plaque burden (3).

TREML4 is a member of the TREM receptor family, known modulators of inflammation and immune responses. In fact, most members of the TREM receptor family have been implicated in either the preceding inflammatory state or directly in the pathogenesis of atherosclerosis disease in both humans and mice (14–17). For example, TREM1 is upregulated in dyslipidemia and promotes atherosclerosis by inducing monocytosis and foam cell formation in both humans and mice (16, 18). Furthermore, circulating soluble TREML1, which modulates platelet-endothelial cell interactions and sepsis progression (19), also positively correlates with clinical presentation of acute coronary syndrome (14). Similarly, TREM2 is expressed in a specific macrophage subset in diseased aortas, which have been ascribed to lipid metabolism functions (20). Additionally, TREM2 dampens TLR signaling in several myeloid cell types (21, 22), controls macrophage and microglia function (23, 24) and directly binds apolipoprotein E and other atherogenic lipids (25–27). In fact, multiple members of the TREM family have been shown to exhibit lipid binding properties (28) which makes this family a remarkable target for modulating the intertwined relationship between lipid signaling and inflammation.

*TREML4* expression has been described specifically in splenic macrophages and Ly6C^lo^ monocytes (29, 30) and plays a role in lupus, an autoimmune disease characterized by inflammation in joints, connective tissue, and organs including the heart (31). Mouse studies suggest that *Treml4* potentiates Toll-like receptor 7 (Tlr7) signaling in a model of systemic lupus erythematosus and genetic *Treml4* deficiency protects animals from lupus-associated kidney failure, increased levels of inflammatory cytokines, and autoantibodies.

Here, we study the role of *Treml4* in pro-inflammatory macrophages and atherosclerosis progression; both key underlying aspects of most cardiovascular diseases. Transcriptome analysis in human and murine macrophages shows that *TREML4* is preferentially expressed in inflammatory macrophages and that it correlates with dysregulation of several inflammatory pathways, including those associated with leukocyte activation and extracellular matrix degradation. In a murine model of atherosclerosis, we found that *Treml4* favors development of disease by exacerbating lesion burden, macrophage content, stage scores, and vulnerability features of advanced atherosclerotic lesions. Finally, we have found that several inflammatory pathways potentially related to cardiovascular disease are regulated by *Treml4* in oxLDL-loaded murine macrophages, suggesting a role for *Treml4* in macrophage iron homeostasis and glucose metabolism. These results put forward unanticipated roles for *Treml4* in inflammation and atherosclerotic plaque stability.

## Materials and Methods

### Isolation and Treatment of Human Blood Monocytes

Human blood was obtained from healthy volunteers recruited through the National Cancer Institute-Frederick Research Donor Program (IRB approval number 16-003) in accordance with the Declaration of Helsinki and provided written informed consent under IRB-approved protocol. Donors were carefully sex-matched in all haplotypes for all studies. Detailed methods for macrophage differentiation, genotyping and treatment can be found in Supplementary Material.

### RNA-Sequencing and Data Processing

Murine bone marrow-derived macrophages or human blood-derived PBMCs and macrophages were isolated as described above. For mouse LPS experiments, two biological replicates per genotype were analyzed whereas for humans, 5 carriers for the minor allele in rs2803496 (permissive) and 5 non-carriers (non-permissive) donors were used. RNA-seq libraries were prepared using TruSeq Stranded RNA-Seq with Poly (13) selection using HiSeq2 (126 bp, PE). For oxLDL-treated macrophages, cells were differentiated as describe above and treated with two different concentrations of oxLDL for 24 h. Eland aligner was used for BAM files generation and converted to fastq files using NISC bam2fastq tool. Additional processing was done with CCBR RNAseq ver2 workflow (STAR-soft trimming, RnaSeqC, Subred, Deseq2, EdgeR and Limma). For differentially expressed genes analysis, Deseq2 *p*-values were corrected for multiple comparisons using the Benjamini Hochberg False Discovery Rate (FDR) calculated with the MSCL Toolbox in the JMP statistical discovery software. Three gene lists were generated for the mouse data. Genes on each of the three gene lists were found to be significantly differentially expressed if the gene passed a 5% FDR and an absolute fold change of 1.4 or greater for each of the 3 comparisons. Four gene lists were generated for the human data. Genes on each of the four gene lists were found to be significantly differentially expressed if the gene passed a 15% FDR and an absolute fold change of 1.4 or greater for each of the 4 comparisons of interest. Hierarchical clusters were generated using the JMP Statistical Discovery software. Data were normalized by the row mean and the Ward minimum variance method was used as the distance measure. Volcano plots show the log *p*-value versus the log2 fold change for each comparison of interest. Genes passing the filters mentioned above are highlighted. To determine enriched ontology pathways in all comparisons, Metascape standard analysis was performed https://metascape.org. The dataset generated for this study can be found in the Gene Expression Omnibus (GEO) under superseries GSE145384. Additional details can be found in Supplementary Material.

### Mice and Diet

*Apoe^–/–^/Treml4^–/–^* mice were generated at the Frederick National Laboratory Core Breeding Facility by crossing *Apoe*^–/^*^–^* mice (Jackson Laboratory, Bar Harbor ME) and *Treml4^–/–^*, generously provided by Dr. Juliana Idoyaga (30). Double mutant mice were intercrossed for ten generations. All animals were housed under specific pathogen–free conditions. At 6–8 weeks of age, mice were placed on RD Western Diet (Cat# D12079B, Research Diets, Inc., New Brunswick, NJ, United States). All animals used in this study were age and sex-matched except for the polarized microscopy studies and qPCR analysis of diseased aortas where all females or all males were used, respectively. Animal care was provided in accordance with procedures in: “A Guide for the Care and Use of Laboratory Animals”. Ethics approval for the animal experiments detailed in this manuscript was received from the Institutional Animal Care and Use Committee (Permit Number: 000386) at the NCI-Frederick. Mice were anesthetized with 0.1 ml of a ketamine/xylazine mixture IP injection before all procedures and adequacy monitored by paw withdrawal reflex.

### Mouse Bone Marrow Isolation, Culture, and Treatments

Detailed methods can be found in Supplementary Material.

### Flow Cytometry and Blood Cell Counts

After detachment, cells were transferred to flow cytometry tubes, washed with FACS buffer, blocked with anti-Fc receptor antibody 2.4G2 and stained with PE anti-mouse Treml4 antibody (Clone 16E5,1:50, BioLegend, San Diego, CA, United States) and APC anti-mouse CD36 (Clone HM36, 1:100, BioLegend, San Diego, CA, United States). Samples were analyzed by flow cytometer (Becton-Dickinson SORP or Fortessa). Doublets and debris were gated out and dead cells were excluded by DAPI before quantification of median fluorescent intensities with FlowJo (FlowJo, LLC, Ashland, OR, United States). Whole blood analyses were performed in EDTA-treated blood before red blood cell lysis. Briefly, 40 μl of blood was washed with FACS, blocked, and stained with antibody cocktail (CD45, F4/80, Ly6G, Ly6C; all from Biolegend). Technical assistance for hematology was provided by the Pathology/Histotechnology Laboratory, LASP, Frederick National Laboratory for Cancer Research/Leidos Biomedical Research Inc.

#### Histological Analyses of Atherosclerosis Progression, Calcified Cores, and Calcium Content

Detailed dissection, perfusion and tissue collection methods can be found in Supplementary Material. Sections for every ten steps were selected for Oil Red O, hematoxylin and Movat Pentachrome staining (Sigma-Aldrich, St. Louis, MO and Polyscience, Inc., Warrington, PA, United States, respectively). Additional slides where all the three sinus leaflets were observed were used for immunohistochemistry, collagen, and von Kossa staining. The brachiocephalic artery (BCA) was excised from perfusion-fixed mice and mounted in paraffin for sectioning. 8–10 μm paraffin sections were stained with Movat per manufacturer instructions. Calcified cores were identified and quantified in at least four sections per mouse, ten steps apart throughout the length of the aortic root. For calcium content, slides were incubated with 1% Aqueous Silver Nitrate Solution (Sigma-Aldrich) and placed under ultraviolet light for 20 min. Unreacted silver was removed by performing several rinses with distilled water and 5% Sodium Thiosulfate (Sigma-Aldrich) for 5 min, counterstained with 0.1 % Nuclear Fast Red Solution (Sigma-Aldrich) for 5 min, dehydrated through graded 70–100% alcohols, and cleared with Xylene. A coverslip was applied using EcoMount Mounting Media and scanned using ZEISS Axio Scan Z1 Slide Scanner, model AX10.

### Immunofluorescence Staining

Aortic sinus cryosections were permeabilized with 0.05% Triton X, blocked with 5% BSA for 30 min and incubated with Rat anti-mouse MOMA-1 (1:200, Thermo Fisher Scientific, United States) overnight at 4°C. After extensive washing, sections were incubated with goat anti-rat FITC-conjugated IgG antibody (1:500, Jackson ImmunoResearch, West Grove, PA) for 1 h. Sections were counterstained with DAPI (1:1000) and mounted with fluorescence mounting media (Dako North America, Inc., Santa Clara, CA, United States). For immunostaining of CD107b, smooth muscle actin (SMA) and matrix metalloproteinase 9 (MMP9), paraffin embedded sections were used. Briefly, slides were de-paraffinized and antigen retrieval was performed by boiling 1X EDTA solution, pH 8.0 (Fisher bioreagents) for 10 min. Peroxidase activity was blocked using 3% H_2_O_2_ (Sigma-Aldrich) for 5 min, followed by incubation with Avidin-biotin complex (Avidin/Biotin Blocking Kit, Vector Laboratories) for 30 min, and blocked with Dako Protein Block, serum-free Solution (Agilent) for 10 min. Slides were incubated overnight at 4°C with Purified rat anti-mouse CD107b (MAC3, BD Pharminger, 553322, 1:50), monoclonal anti SMA, mouse absorbed (Dako, 1:200) and Polyclonal rabbit anti-Mmp9 (Abcam, 1:1600). After incubation of secondary antibodies, slides were developed with ImPACT NovaRED Peroxidase (HRP) substrate (Vector Laboratories), and counterstained with Dako or Hematoxylin for 2 min.

### Lesion Scoring and Morphometric Analysis

Measurements of calcification, lesion size, foam cell area, and stenosis were performed on ZEN software (Zeiss, United States). Data represents mean lesion area from at least three sections per mouse. Lesion severity was scored using a system modified from Stary et al. by two pathologists blinded to the phenotypes (32). Scores for each leaflet were averaged to represent a global lesion stage score per mouse or presented individually for each genotype. Individual plaque scores were tabulated as frequency for the type of lesion. Picrosirius red staining positive area and fluorescence intensities was quantified with Image J software. Calcium-positive staining and additional quantifications were done in scanned images with Halo Imaging Software. Parameters were scored or quantified in at least three sections in the same location per mouse. Polarized light microscopy methods can be found in Supplementary Material.

### Serum Cholesterol and Cytokine Analysis

Detailed methods for cholesterol and cytokines analysis can be found in Supplementary Material.

### Quantitative PCR Analyses

Total RNA was extracted from whole thoracic aortas after PBS perfusion using Trizol and purified using the high pure RNA isolation kit (Roche, United States), or the kit alone for culture cells. cDNA was synthetized from 1 μg total RNA by the High Capacity cDNA Reverse Transcriptase Kit (Roche, United States) according to manufacturer. All PCR reactions were performed with TaqMan Universal PCR Master Mix (Roche, United States). Mean values of two technical replicates were expressed as relative expression using the ΔΔCt method. TaqMan Gene Expression Assay probes (Thermo Fisher Scientific/Applied Bioscience) Probe information can be found in Supplementary Material.

#### Metabolomics Analysis

After treatment, 10 × 10^6^ bone marrow-derived macrophages were pelleted and snap-frozen in liquid nitrogen. Samples were further processed and analyzed at West Coast Metabolomics Center (University of California, Davis, Davis, CA, United States) as previously described (33). Samples were further normalized using the sum of peak heights for all individual identified metabolites.

### Statistical Analyses

Statistical analyses were performed using GraphPad Prism 6 (La Jolla, CA, United States). Data are represented as mean ± standard error of the mean (SEM) or paired data points (as indicated) and were analyzed with either paired or unpaired Student’s *t*-test for comparison between two groups and Two-way ANOVA with either Sidak’s or Dunnett’s multiple comparisons test. Scoring data was analyzed by Mann-Whitney test or chi-squared test for the frequency distribution of individual plaques. All other data were assumed to be normally distributed. Data are considered statistically significant at *p*-value < 0.05.

## Results

### Transcriptional Profiling Reveals TREML4 Is Upregulated in Human and Mouse Inflammatory Macrophages and Regulates Inflammatory Programs

**FIGURE 1:**
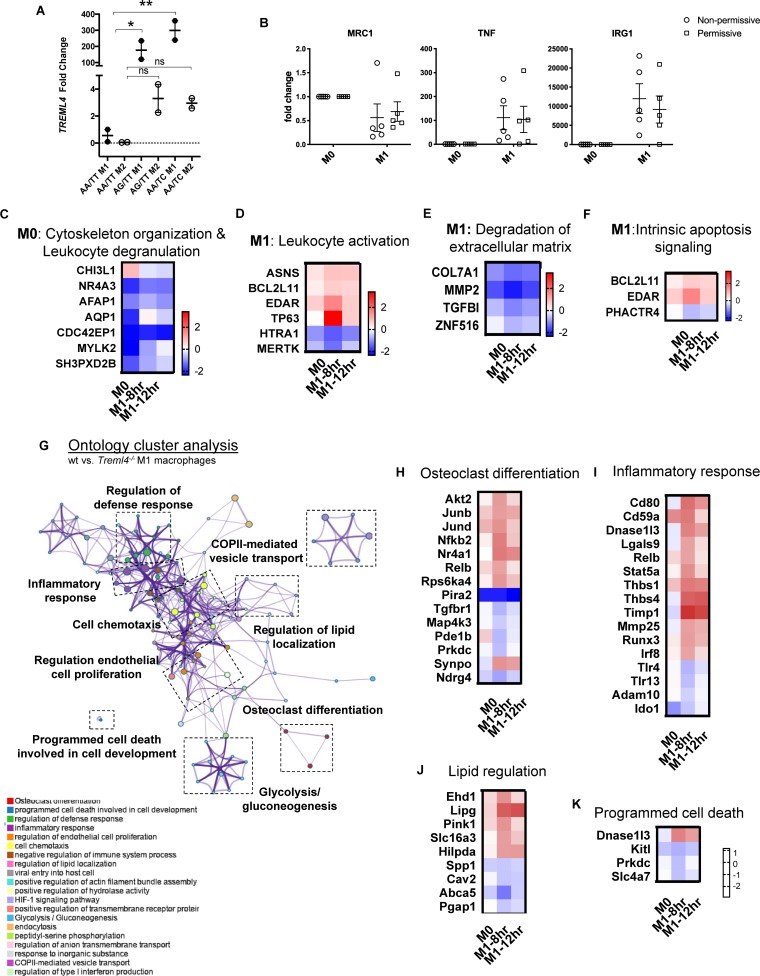
Transcription profiling reveals TREML4 is upregulated in human and mouse inflammatory macrophages and dysregulates inflammatory programs. **(A)** Exploratory analysis of *TREML4* expression by qPCR in the different haplotypes combinations for rs2803495/rs2803496 after either LPS (M1) or IL4 (M2) stimulation for 16 h. Data are represented as fold changes over non-permissive (AA/TT) samples. *P < 0.05, **P < 0.01, ns = not significant, *n* = 2 subjects per haplotype (One-way ANOVA analysis with Dunnett’s multiple comparisons test). **(B)** Confirmatory analysis of qPCR for mannose receptor 1 (MRC1), tumor necrosis factor (TNF) and immune-responsive gene 1 (IRG1) in M1 macrophages permissive for TREML4 expression (AA/TC haplotype). **(C–F)** Expression heatmaps (log2 fold-changes) of DEG in permissive macrophages belonging to indicated enrichment terms: *Actin Cytoskeleton Organization* in M0, *Leukocyte Activation* in M1, *Degradation of Extracellular Matrix* in M1 and *Intrinsic Apoptosis Signaling* in M1. **(G)** Enrichment ontology clusters of DEG in murine M1-BMDM as visualized by Metascape after enrichment with GO-Biological Processes, KEGG Pathway and Reactome ontology sources. The most statistically significant term within similar term clusters (enclosed by interrupted lines) is chosen to represent the cluster. **(H–K)** Heatmaps of log_2_ fold changes in *WT* compared to *Treml4^–/–^* M1 macrophages. Selected ontology terms; *Osteoclast Differentiation*, *Inflammatory Response*, *Lipid Regulation* and *Programmed Cell Death*.

We previously reported that *TREML4* expression in human blood was associated with two eQTL, rs2803495 and rs2803496, and that carriage of either minor allele (G for rs2803495 and C for rs2803496) was associated with higher *TREML4* expression and higher CAC scores (9). We termed donors carrying one or both minor alleles “permissive” for *TREML4* expression. Detailed analyses suggested substantial expression in neutrophils of permissive donors, however, inflammatory macrophages are the predominant leukocyte subset in cardiovascular disease. Thus, we asked if *TREML4* expression was induced during macrophage polarization. An exploratory analysis was performed using isolated peripheral blood monocytes (PBMC’s) from healthy donors of the different haplotypes for both SNP’s and TREML4 expression was examined after macrophage maturation and overnight treatment with either LPS/IFNγ (M1) or IL4 (M2) (Figure 1A). As compared to macrophages from non-permissive individuals (not carrying the minor allele for either SNP), cells from permissive donors showed an increase of up to 300-fold *TREML4* expression after M1 polarization, while no significant increase was seen after M2 polarization. We next investigated whether *TREML4* expression directly affected M1 polarization markers by qPCR and found that *TREML4* expression does not affect upregulation of *TNF* or *IRG1* or the correspondent downregulation of *MRC1* in M1 macrophages (Figure 1B).

To gain additional insight into the gene expression profile controlled by *TREML4* expression in inflammatory macrophages, we performed RNA-sequencing analysis on human blood-derived macrophages from donors carrying the permissive haplotype which showed dramatically higher (100–300 fold increase) *TREML4* expression after M1 polarization (AA/TC) as compared to only slight induction (2–3 fold) by IL4 (Figure 1A). As expected, M0 macrophages of permissive and non-permissive donors are remarkably similar, with only 22 differently expressed genes (DEG) (Supplementary Figure S1A) and only 4 of these genes remained dysregulated in all comparisons (*TREML4, GPAT2, LINC00431, SIGLEC12)*. However, after activation for 8 h (M1-8 h), we found 31 DEG (Supplementary Figures S1A,B and Supplementary Table S1) whereas only 4 of these genes, namely *COL7A1*, *TREML3*, *RSU1P1*, and *FRMD4A*, remained significantly changed after 12 h. At this late activation timepoint, only 7 additional genes were detected as being significantly dysregulated (Supplementary Figure S1B and Supplementary Table S1). Thus, we chose the M0 and M1-8 h DEG list to perform pathway analysis. This analysis revealed five distinct enriched pathways (Figures 1C–F and Supplementary Table S2). Surprisingly, M0 and M1 macrophages from permissive donors showed reduced expression of 6 of 8 genes annotated as members of the *Actin Cytoskeleton Organization*, and increased expression of several genes involved in *Leukocyte Activation* (Figures 1C,D and Supplementary Table S2). Additionally, M1-8 h permissive cells showed reduced expression of all gene members of the *Degradation of Extracellular Matrix* pathway, namely *Col7A1, MMP2, TGFBI and ZNF516* (Figure 1E and Supplementary Table S2), while two out of three genes members of the *Intrinsic Apoptosis Signaling* pathway remained highly expressed in both M0 and M1 cells from permissive donors (Supplementary Table S2). Taken together our data show a transcriptional profile regulated by *TREML4* in inflammatory macrophages which suggests a very specific regulatory role for TREML4 in inflammatory cells. Thus, in order to gain additional insight into whether human and mouse Treml4 expression might regulate similar pathways, we performed a similar analysis in murine macrophages. Similar to our findings in human macrophages, M0 macrophages from WT and *Treml4*^–/^*^–^* mice were transcriptionally similar, with only 15 DEG when using a cutoff of 1.4-fold change and false discovery rate of 5% (Supplementary Figures S1A,C and Supplementary Table S3). Of the dysregulated genes at basal conditions, only three genes, Ankyrin1 (*Ank1)*, *Gm10693* and Plasminogen activator inhibitor 2 (*Serpinb2)* remained dysregulated after M1 stimulation in *Treml4^–/–^* macrophages (Supplementary Figure S1D, asterisks). Additionally, in M1 macrophages a total of 194 genes were significantly dysregulated in *Treml4*^–/^*^–^* macrophages as compared to wild type (Supplementary Figures S1A,C and Supplementary Table S3). Of the 181 genes specifically related to the M1 response at 8 h, only 13 genes were still dysregulated after 12 h with an additional 34 genes at this late time point (Supplementary Figure S1A and Supplementary Table S3). Pathway analysis of the transcriptional profile of M1 macrophages after 8 h revealed that *Treml4*-dependent gene expression is associated with several pathways related to the inflammatory response and carbohydrate metabolism (Figure 1G and Supplementary Table S4). Visualization of these enriched pathways using Metascape shows that while most pathways appeared to be co-regulated (by cluster proximity), carbohydrate metabolism and cell development pathways were distinctively regulated. Most notably, we found that DEG members of the *Osteoclast Differentiation* pathway were markedly higher in wt cells, with key genes such as AKT Serine/Threonine Kinase 2 (*Akt2)*, transcription factors *Junb*, *Jund and Relb* and Nuclear Factor Kappa B2 (*Nfkb2)* showing the highest levels of *Treml4*-mediated enhancement (Figure 1H). Similarly, most genes in the *Inflammatory Response pathway* were upregulated by *Treml4* expression (Figure 1I) while Toll-like receptor 4 and 13 (TLR4, TLR13) were lower in wt cells. Importantly, several genes in the *Inflammatory Response* pathway are also involved in extracellular matrix remodeling, namely Thrombospondin 1 and 4 (*Thbs1*,*Thbs4*), Tissue inhibitor of metalloproteinases 1 (*Timp1)*, Matrix metalloproteinase 25 (*Mmp25)*, and Runt related transcription factor 3 (*Runx3)* and these all showed higher expression in wt cells. *Treml4* expression also affects genes of the *Lipid Regulation* pathway, including Lipase G (*Lipg)*, GPI inositol-deacylase (*Pgap1)*, ATP-binding cassette A5 (*Abca5)*, Hypoxia inducible lipid droplet-associated (*Hilpda)*, and Caveolin 2 (*Cav2)* (Figure 1J). Finally, we found that *Treml4* expression downregulated most genes in the *Programmed Cell Death Involved in Cell Development* pathway (Figure 1K). Importantly, canonical markers of macrophage polarization were not differentially expressed at any timepoint, which suggests that classical polarization was not altered by *Treml4* status. Taken together, our data show that *Treml4* is selectively overexpressed by mouse and human inflammatory macrophages and supports several macrophage inflammatory programs.

### *Treml4* Is Expressed in Atherogenic oxLDL-Loaded Macrophages and in Diseased Aortas and Affects Plaque Burden in *Apoe^–/–^* Mice

**FIGURE 2:**
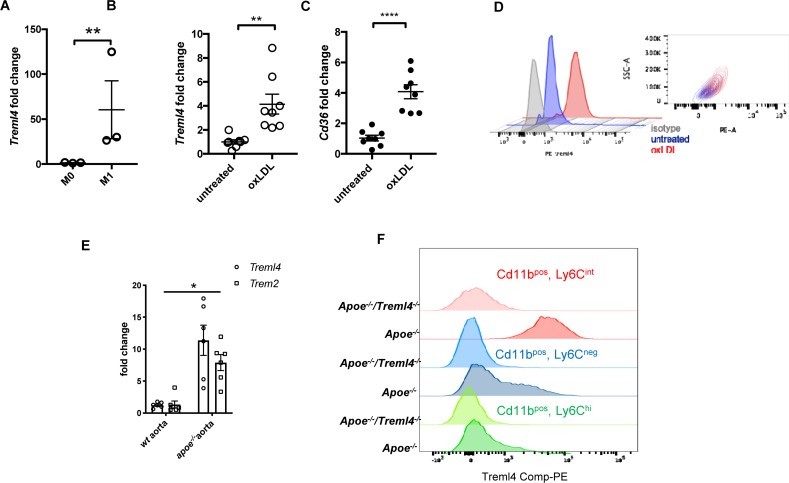
*Treml4* is expressed in atherogenic oxLDL-loading of BMDM and in diseased aortas of *Apoe^–/–^.* **(A)** *Treml4* expression in BMDM after no stimulation (M0) or LPS/IFNγ(M1) for 16 h. **P < 0.01, *n* = 5 biological replicates (unpaired Student’s *t*-test). **(B,C)** Fold change expression of *Treml4* and *Cd36* in BMDM after overnight incubation with oxLDL (30 μg/ml) in lipoprotein-free media, **p < 0.01, ****p < 0.0001, *n* = 8 biological replicates (unpaired Student’s *t*-test). **(D)** Flow cytometry analyses of Treml4 expression in BMDM after overnight treatment with oxLDL. Representative plot of two independent experiments, *n* = 3. **(E)** qPCR analyses of Treml4 and Trem2 expression in thoracic aorta from wt and Apoe*^–^*^/^*^–^* mice. *P < 0.05, *n* = 5 male mice per genotype (unpaired Student’s *t*-test). **(F)** Flow cytometry analysis of Treml4 expression in Cd11b+, Ly6C^hi^, Ly6^neg^ or LyC6^int^ monocyte population in *Apoe^–/–^* mice.

In our RNA-sequencing analysis of M1 macrophages, we noted that several dysregulated pathways have been shown to be relevant in cardiovascular diseases, particularly in the development of atherosclerosis. This, together with our previous demonstation of association between TREML4 polymorphisms and cardiovascular calcification led us to ask whether *Treml4* might contribute to atherosclerosis progression. Consistent with our findings in human blood-derived macrophages, BMDM expressed little *Treml4* until M1 polarization (Figure 2A). Since macrophage activation during the development of atherosclerosis is driven by LDL particles, particularly oxidized LDL (oxLDL), we tested the ability of oxLDL to induce *Treml4* expression in murine BMDM. Treatment of BMDM for 24 h with oxLDL resulted in significantly increased *Treml4* mRNA and surface protein expression (Figures 2B,D). *Treml4* expression in oxLDL-treated macrophages correlated with expression of *Cd36*, the main scavenger receptor induced by oxLDL during foam cell formation (34) (Figure 2C). To determine if *Treml4* was expressed in atherosclerotic plaques, we harvested arteries from *Apoe^–/–^* mice, which develop spontaneous atherosclerotic lesions. While qPCR analysis in whole aortas of 20-week-old *Apoe^–/–^* mice showed no *Treml4* upregulation when compared to WT (Supplementary Figure S1), we found *Treml4* expression to be significantly induced in the *Apoe^–/–^* mice when fed a WD for 16 weeks. Increased *Treml4* expression was similar to *Trem2* expression levels, which has been previously found to be induced in diseased aortas (Figure 2E) (20). Although we could not directly assess Treml4 expression in the vessel due to technical limitations, the most likely explanation for this data is that *Treml4* is expressed in inflammatory macrophages, elicited by proatherogenic lipoproteins, and found within atherosclerotic lesions during disease progression.

**FIGURE 3:**
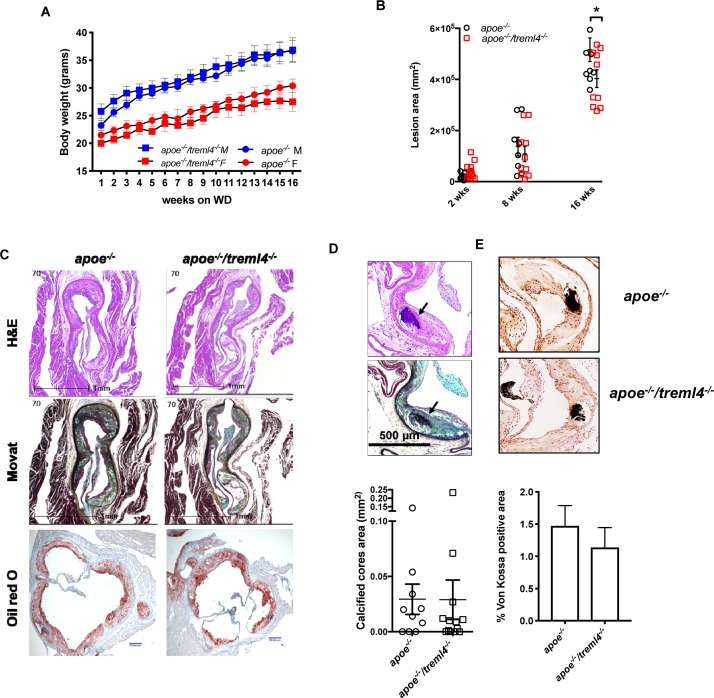
*Treml4* affects plaque burden but not calcification in the Apoe*^–^*^/^*^–^* mice. **(A)** Body weight progression of male (blue symbols) and female (red symbols) Apoe*^–/–^* and *Apoe^–/–^/Treml4^–/–^* mice after 16 weeks WD, *n* = 10 per genotype (Two-way ANOVA with Sidak’s multiple comparison test). **(B)** Lesion area progression for 2, 8, and 16 weeks after WD. *P < 0.05, *n* = 10 and 12 at 2 weeks, *n* = 8 and 10 at 8 weeks, *n* = 10 and 13 at 16 weeks for *Apoe^–/–^* mice and *Apoe^–/–^/Treml4^–/–^* mice, respectively (Two-way ANOVA with Sidak’s multiple comparison test). Both male and female mice are represented here. **(C)** Representative images of aortic sinus sections stained with hematoxylin and eosin, Movat Pentachrome, and Oil Red O (ORO) of *Apoe^–/–^* and *Apoe^–/–^/Treml4^–/–^* mice after 16 weeks of WD feeding. Original magnification: 200X, scale bar = 1 mm or 200 μm for ORO. **(D)** Representative images of histological identification of calcified cores (indicated by black arrows) within atherosclerotic lesions after 16 weeks of WD and quantification of positive areas. **(E)** Representative images of von Kossa staining and quantification of positive areas (unpaired Student’s *t*-test).

To investigate whether atherosclerosis progression was affected by *Treml4* expression, we assessed early, intermediate, and advanced disease stages in *Apoe^–/–^/Treml4^–/–^* knockout mice and *Apoe^–/–^* controls after feeding a WD for 2, 8, and, 16 weeks. We first corroborated the expression profile of *Treml4* in peripheral monocytes of the *Apoe^–/–^* mice after WD and found that, similar to previous reports, *Treml4* expression was confined to CD11b+, Ly6C^low–int^ cells (Figure 2F). During WD feeding, serum cholesterol levels increased over time but they remained similar between the genotypes (Table 1). Similarly, weight and food consumption remained unchanged in the *Apoe^–/–^/Treml4^–/–^* mice as compared to the *Apoe^–/–^* mice during the study (Figure 3 and Supplementary Figure S1C). Cross-sectional analyses of the aortic root after 2 or 8 weeks of WD showed that total plaque area was similar in both genotypes (Figure 3B). However, we found that at advanced disease stage (16 weeks WD), *Apoe^–/–^/Treml4^–/–^* mice have slightly decreased plaque areas compared to controls (Figures 3B,C). In accordance with other studies, male mice showed the greatest difference in this parameter (data not shown).

**TABLE 1:** Cholesterol levels of *Apoe^–/–^* and *Apoe^–/–^/Treml4^–/–^* after WD.

	Cholesterol levels (μg/ml)		
	2 weeks	8 weeks	16 weeks
*Apoe^–/–^*	696.5 ± 30.09	884.8 ± 86.95	1238 ± 132.1
*Apoe^–/–^/Treml4^–/–^*	752.3 ± 39.95	736.5 ± 55.23	1051 ± 111.9

*Data represented as mean ± SEM, N = 5 for 2 weeks, N = 13–14 for 8 weeks and N = 16 for 16 weeks. Analyzed by two-way ANOVA with Šidák ‘s multiple comparison test.*

The genetic associations between *TREML4* eQTLs and disease we have previously described showed correlations between the permissive genotypes, *TREML4* expression and the extent of coronary artery calcification. In murine models of atherosclerosis, calcification is a very late event only evident after 16–20 weeks of WD (35). Therefore, we assessed atherosclerotic calcification in the *Apoe^–/–^/Treml4^–/–^* mice after 16 weeks of WD. Although calcified cores were easily distinguished within plaques, they were rare even in the *Apoe^–/–^* mice (Figure 3D). Unexpectedly, histological and morphological analyses of the aortic root showed no differences in calcified core areas (Figure 3D), frequency of lesions containing calcified cores (Supplementary Figure S2A) or microcalcifications detected by von Kossa staining (Figure 3E), between the two strains. Thus, in this model, while *Treml4* expression affect lesion size in advanced disease, it does not affect calcification in aortic root lesions.

### *Treml4* Effects on Lesion Stage

**FIGURE 4:**
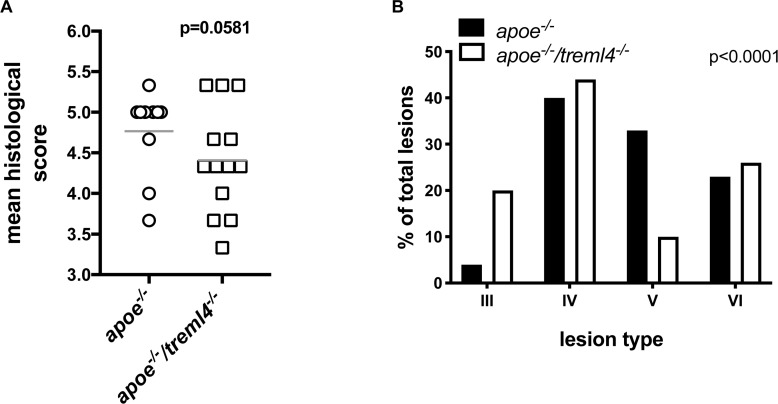
*Treml4* effect on lesion stage after 16 weeks of western diet feeding. **(A)** Atherosclerosis mean histological scoring in the aortic sinus of *Apoe^–/–^* and *Apoe^–/–^/Treml4^–/–^* mice. *P* = 0.0581, *n* = 10 *Apoe^–/–^*, *n* = 13 *Apoe^–/–^/Treml4^–/–^* (Mann-Whitney *U* test). **(B)** Plaque phenotype distribution based on histological staging of individual lesions in all mice, *p* < 0.0001 (Chi-square test).

Because calcification is only one characteristic of late atherosclerosis, we next investigated if *Treml4* influenced plaque composition, a key consideration for disease outcome. To this end, atherosclerosis lesion stage within the aortic root was scored by two pathologists blinded to the genotypes using a modification of an established scoring system (32) (Table 2). We found that the average histological score, for the entire aortic root, was lower in the *Apoe^–/–^/Treml4^–/–^* mice when compared to controls after 16 weeks of WD (Figure 4A). Although this result did not reach statistical significance (*p* = 0.0581), these findings suggest there may be underlying changes in individual plaque stages. Therefore, we evaluated the stage frequency of individual plaques in the aortic root as described in Table 2 (Figure 4B and Supplementary Table S5). We found that 20% of individual plaques evaluated from *Apoe^–/–^/Treml4^–/–^* mice were Type III plaques, whereas only 3.3% of plaques in *Apoe^–/–^* mice were at this stage, which represents an early lesion phenotype with some pathological intimal thickening and extracellular lipid pools. In fact, most *Apoe^–/–^* lesions have instead progressed to the advanced atheroma stage (Type IV–VI). Interestingly, the lesion frequency was most different for Type V plaques, with only 10.3% of the *Apoe^–/–^/Treml4^–/–^* lesions at this stage compared to 33.3% of the *Apoe^–/–^* lesions. Type V plaques are of particular interest because they are composed of multilayered atheroma, a less defined lipid core, fewer inflammatory cells, and fibrotic material deposition. In agreement with our direct assessment of calcification, the frequency of Type VI plaques, which almost exclusively contain calcified material, was similar between the *Apoe^–/–^/Treml4^–/–^* and the *Apoe^–/–^* lesions (23.3% vs. 25.6%). These phenotypic changes were only found in atherosclerotic lesions after 16 weeks of WD since lesion stage was similar in both genotypes after 2 and 8 weeks of WD feeding (Supplementary Figure S3). Taken together, these data suggest *Treml4* facilitates a more severe plaque phenotype at advanced disease stages.

**TABLE 2:** Scoring system used in this study.

Lesion score	Nomenclature	Histopathological classification
1	Intimal thickening	Microscopic lipid droplets, small groups of macrophage foam cells
2	Intimal xanthoma	Fatty streaks, layers of foam cells; some lymphocytes
3	Pathological intimal thickening	Extracellular lipid pools among layers of smooth muscle cells
4	Fibrous cap atheroma	Well-defined lipid core
5	Fibrocalcific plaque	Fibrotic or collagen-rich plaque, few inflammatory cells, necrotic core and/or calcification possible
6	Plaque erosion, calcified nodule	Lesion with surface defect, hemorrhage or thrombosis

### *Treml4* Effect on Lesional Macrophage Content and Monocytic Populations

**FIGURE 5:**
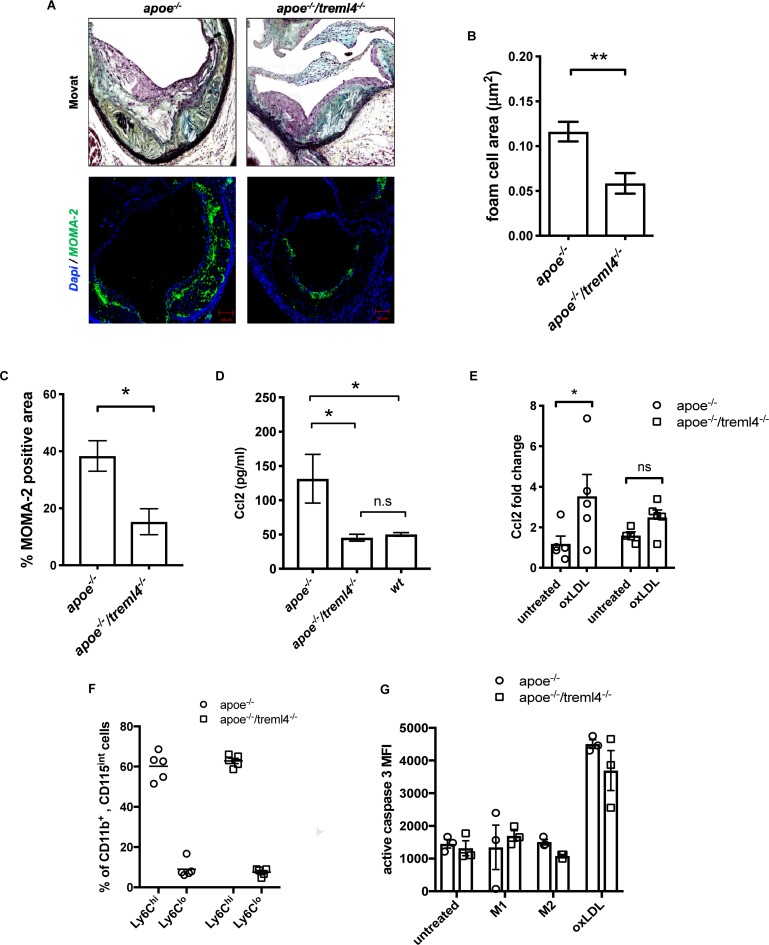
The effect of *Treml4* on lesional macrophages and monocytic populations. **(A)** Representative images of foam cell/macrophage areas in Movat pentachrome-stained or MOMA-2 immunofluorescence in aortic sinus lesions, scale bar = 100 μm. **(B)** Histological quantification of foam cell areas within lesions and **(C)** percentage of MOMA-2 positive area. *P < 0.05, **P < 0.01 (Student’s *t*-test). **(D)** Serum Ccl2 levels in 16 week western diet-fed mice. *P < 0.05, ns = not significant, *n* = 5 per genotype (One-way ANOVA analysis with Tukey’s multiple comparison test). **(E)** Ccl2 fold change in bone marrow-derived macrophages left untreated or treated with 25 μg oxLDL overnight. **(F)** Percentage of circulating Ly6C^hi^ and Ly6C^lo^ monocytes in 10 weeks WD mice, *n* = 5. **(G)** Caspase 3 activity by flow cytometry in BMM from *Apoe^–/–^* and *Apoe^–/–^/Treml4^–/–^* after treatment with 100 ng/μl LPS (M1), 50 ng/μl IL4 (M2) or 25 μg oxLDL overnight, *n* = 3 per genotype.

To further investigate the differences in plaque composition between the *Apoe^–/–^* and *Apoe^–/–^/Treml4^–/–^* mice after 16 weeks WD, morphometric and immunohistological evaluation of several plaque characteristics was performed. We found that foam cell area within plaques, which is histologically identified as lipid-laden cells with fibroblastic feature by Movat Pentachrome staining, was significantly decreased in the *Apoe^–/–^/Treml4^–/–^* mice (Figures 5A,B). Indeed, macrophage immunostaining with anti-monocyte/macrophage antibody (MOMA-2) showed a significant decrease in macrophage-positive areas in the *Apoe^–/–^/Treml4^–/–^* mice (Figure 5A, bottom panel and C) while staining for neutral lipid deposition by ORO showed no significant changes between the two genotypes (Supplementary Figure S2B). Consistent with the lower foam cell and macrophage positive area, we found that *Apoe^–/–^/Treml4^–/–^* mice had lower levels of circulating Ccl2 (Figure 5D) while other cytokines relevant to atherosclerosis remained unchanged (Supplementary Figure S4). Consistent with this decrease in circulating Ccl2, we found that *Apoe^–/–^/Treml4^–/–^* BMDM failed to upregulate Ccl2 upon oxLDL treatment to the same extend as *Apoe^–/–^* BMDM (Figure 5E).

Because of the observed difference in foam cell and macrophage areas in the plaques and since murine *Treml4* expression in the periphery is largely specific to the Ly6C^low–int^ population, we investigated whether monocytic populations might be skewed in the *Apoe^–/–^/Treml4^–/–^* mice. However, we found that both circulating monocyte number and frequencies of Ly6C^lo^ and Ly6C^hi^ monocytes were similar between the two strains after WD feeding (Figure 5F and Supplementary Figures S4A,B) as was the intermediate population (Ly6C^int^ monocytes, data not shown). We also investigated if *Apoe^–/–^/Treml4^–/–^* macrophages might be more susceptible to oxLDL-induced apoptosis or arrested cell cycle progression and found that while oxLDL treatment does induce higher levels of apoptosis as compared to either M1 or M2 polarization, *Treml4* deficiency does not affect caspase 3-activation or cell cycle progression in BMDM treated with oxLDL (Figure 5G and Supplementary Figure S5C). We also did not detect any differences in a cellular areas within lesions in the *Apoe^–/–^/Treml4^–/–^* mice as compared to *Apoe^–/–^* animals (Supplementary Figure S5D). In all, our results suggest that *Treml4* exacerbates plaque macrophage content and circulating Ccl2 levels without affecting circulating monocyte populations or macrophage apoptosis.

### *Treml4* Regulates Extracellular Matrix Deposition

**FIGURE 6:**
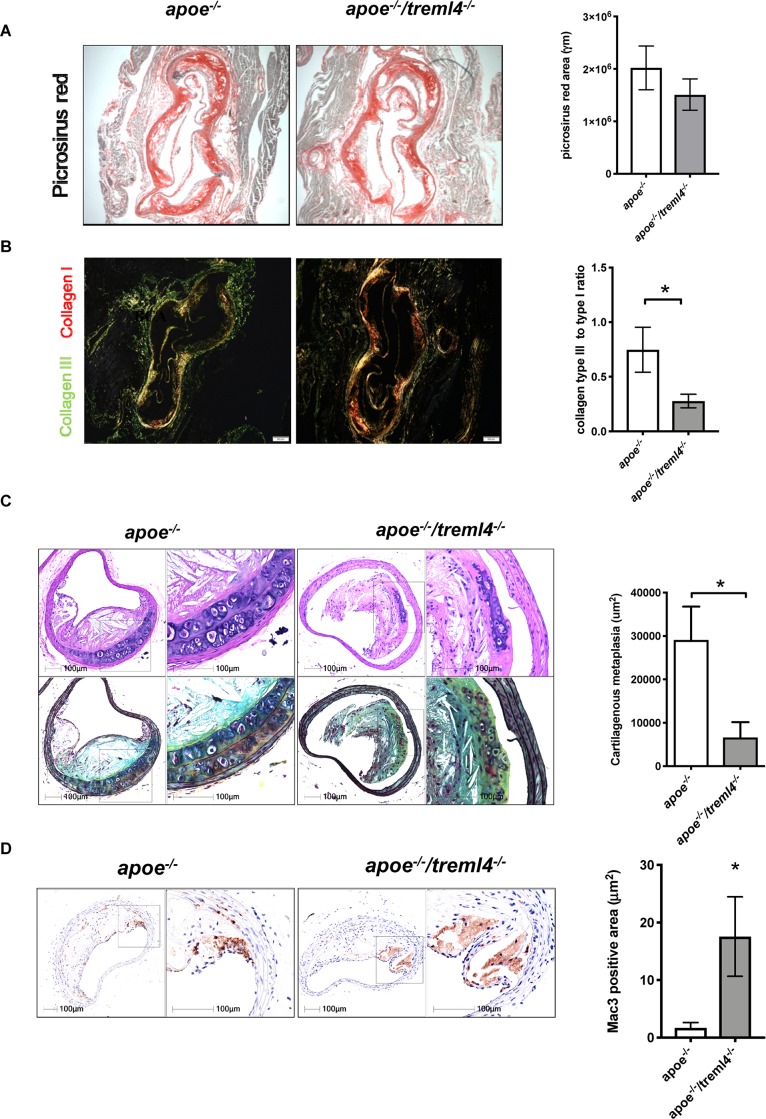
*Treml4 regulates extracellular matrix deposition.* **(A)** Representative images of Picrosirius red staining in aortic sinuses visualized under light microscopy and quantification of total collagen. **(B)** Representative images of Picrosirius red visualized under polarized light and the ratio of collagen type III to type I. *P < 0.05, *n* = 5 males per genotype (Student’s *t*-test). **(C)** Representatives images of Movat Pentachrome-stained brachiocephalic artery sections of mice after 16 weeks of western diet feeding and quantification of cartilaginous metaplasia within atherosclerotic lesions. *P < 0.05, *n* = 4 per genotype (Student’s *i*-test). **(D)** Representative images of Mac-3 positive areas within brachiocephalic artery lesions and quantification of percentage per total lesion area. *P < 0.05, *n* = 4 (Student’s *t*-test).

We next investigated whether extracellular matrix deposition within plaques was altered in the *Apoe^–/–^/Treml4^–/–^* mice. Histological analyses of collagen content within the aortic root by Picrosirius Red staining showed that total collagen content was similar between the two genotypes (Figure 6A). However, collagen maturation may play a role in lesion stability and subsequent thrombosis (36). Polarized light visualization of collagen fibers allows for differentiation of poorly resilient Type III collagen (green appearance) and mature Type I fibers (orange-red appearance). Quantification of polarized light images showed that male *Apoe^–/–^/Treml4^–/–^* mice had increased mature Type I collagen, and therefore a decreased ratio of immature Type III to mature Type I collagen (Figure 6B), suggesting disruptive changes within advanced plaques, as these ratios are often predictors of plaque stability (37). To further assess extracellular matrix deposition, we performed morphometric examinations of the brachiocephalic artery (BCA). At this site, we found that lesion score, plaque size, and stenosis were similar between the two genotypes (Supplementary Figure S6A). However, we noted significantly decreased areas of cartilaginous metaplasia in the BCA of *Apoe^–/–^/Treml4^–/–^* mice (Figure 6C). Cartilaginous metaplasia in the BCA has been shown to be a precursor of vascular calcification in both mice and humans (38, 39). Like calcification, cartilaginous metaplasia is poorly defined. However, a balance in proangiogenic factors, fibroblast populations, and extracellular matrix enzymes has been postulated to play a role in regulating ossification in the vessel wall (40–42). Consistent with these disruptive changes in the BCA, we found a corresponding increase in Mac-3 positive areas in the *Apoe^–/–^/Treml4^–/–^* mice (Figure 6D), which suggest that Treml4 deficiency delays the fibrotic mechanisms in the BCA. Of note, we did not detect any differences in smooth muscle cell actin, VonKossa staining or Mmp9 positive areas in the BCA (Supplementary Figure S6). Thus, our findings suggest that although *Treml4* does not appear to be regulating calcification in the aortic root, the BCA demonstrated significant changes suggesting differential regulation of factors related to fibrotic deposition.

### Transcriptomics and Metabolomics Analysis of Oxidized LDL-Loaded Macrophages Reveals Additional Pathways Controlled by *Treml4*

**FIGURE 7:**
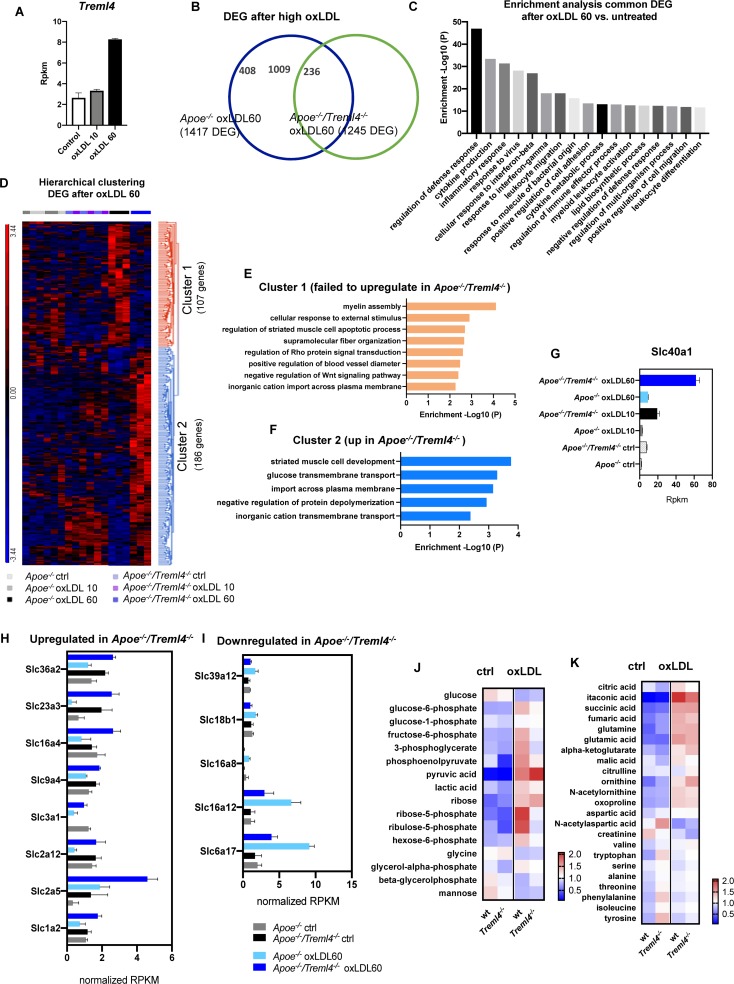
Transcription profiling of oxidized LDL-treated macrophages reveals additional pathways controlled by *Treml4.* **(A)** *Treml4* RPKM counts in *Apoe^–/–^* BMDM after treatment with either low 10 μg/ml (oxLDL10) or high 60 μg/ml (oxLDL60) concentrations for 24 h. **(B)** Venn diagram of differentially expressed genes (DEG) in *Apoe^–/–^* and *Apoe^–/–^/Treml4^–/–^* oxLDL 60 BMDM when compared to their respective untreated control. **(C)** Enrichment analysis of common DEG (1009 genes) between *Apoe^–/–^* and *Apoe^–/–^/Treml4^–/–^* after oxLDL 60 treatment. Top 17 pathways are represented in order of enrichment *p*-value. **(D)** Hierarchical clustering analysis of DEG in oxLDL 60-treated *Apoe^–/–^/Treml4^–/–^* BMDM compared to oxLDL 60-treated *Apoe^–/–^* BMDM. **(E)** Enrichment analysis of cluster 1 and **(F)** cluster 2 from the hierarchical clustering analysis. **(G)** Slc40a1 rpkm counts in all conditions tested. **(H,I)** Overrepresented Slc genes rpkm counts from cluster 1 and 2. Rpkm counts were normalized by the mean across all samples for the individual feature. **(J,K)** Intracellular metabolomics analysis of BMDM treated with 30 μg/ml oxLDL for 24 h. Heatmap represents normalized peak heights per feature.

While it has been shown that the inflammatory pathways elicited by LPS treatment are similar to those driven by foamy macrophages (43, 44), we wanted to better delineate potential mechanism by which *Treml4* might be affecting atherogenesis and changes in lesion composition. We treated BMDM from *Apoe^–/–^* and *Apoe^–/–^/Treml4^–/–^* with either low (10 μg/ml) or high (60 μg/ml) concentrations of oxLDL for 24 h and assessed their transcriptional profile by RNAseq. Preliminary analysis in *Apoe^–/–^* macrophages revealed that while low oxLDL did show a distinct transcriptional profile compared to untreated macrophages and to *Treml4* deficient cells, maximal *Treml4* expression was only achieved at the high oxLDL concentration (Figure 7A and Supplementary Figure S8A), thus we used this concentration for further analysis. When compared to their respective untreated controls, both *Apoe^–/–^* and *Apoe^–/–^/Treml4^–/–^* oxLDL treated BMDM showed dysregulation of more than 1,000 genes, although this number was lower in the *Apoe^–/–^/Treml4^–/–^* comparison (Figure 7B). Of the DEG in *Apoe^–/–^/Treml4^–/–^* upon high oxLDL, 1009 genes were also differentially expressed in a similar manner in the *Apoe^–/–^* macrophages when compared to untreated controls (Supplementary Figure S8B). Enrichment analysis of those 1009 genes showed that several pathways related to the inflammatory response where similarly regulated in both genotypes, suggesting these genes are most likely involved in the macrophage primary response to oxLDL (Figure 7C). We then focused on the DEG between the genotypes exclusively after high oxLDL since this comparison would reveal affected genes associated with *Treml4* status after stimulation (Figure 7D and Supplementary Table S6). We found a total of 293 significantly dysregulated genes between the genotypes after oxLDL. Hierarchical clustering analysis revealed that of those DEG, 107 genes failed to upregulate in *Apoe^–/–^/Treml4^–/–^* BMDM after high oxLDL (cluster 1). Importantly, the expression of these genes at baseline and after low oxLDL was heterogenous and, overall, remarkably similar between the genotypes, confirming that the upregulation of these genes was indeed exclusively related to high *Treml4* expression upon lipid loading. Enrichment pathway analysis revealed that the top 5 pathways affected by the lack of *Treml4* were *Myelin Assembly*, *Response to External Stimulus*, *Regulation of Muscle Cell Apoptosis, Supramolecular Fiber Organization* and *Regulation of Rho* (Figure 7E). Additionally, we found that several genes related to apoptotic processes or negative regulators of Wnt signaling were at the top of the list for genes upregulated by *Treml4* expression, namely *Sox9*, *Tenm4*, *Sfrp2*, *Dkk2*, and *Casp12*, suggesting *Treml4* facilitates either autocrine or paracrine regulation of cell survival in the context of lipid overload. We also identified a subset of 186 genes that were exclusively upregulated in the absence of *Treml4* (cluster 2) (Figure 7D and Supplementary Table S6) which enriched pathways related to *Muscle Cell Development*, *Glucose Transmembrane Transport*, *Import Across Plasma Membrane*, *Negative Regulation of Protein Depolymerization*, and *Inorganic Cation Transmembrane Transport* (Figure 7F). Interestingly, the top enriched pathway by the genes in cluster 2, *Striated Muscle Cell Development*, counteracts the third enriched pathway in cluster 1, *Striated Muscle Cell Apoptotic Process*. Indeed, when we investigated the genes in both pathways (*Adra1a, Capn3, Cxadr, Ttn, Xk, Fhod3, Wfikkn2, Casp12, Sfrp2, Fbxo32*), we found four of the genes to also have membership in the *Cardiac Muscle Cell Development* Pathway.

Interestingly, several genes encoding members of the solute carrier (SLC) family were overrepresented in clusters 1 and 2. We found that the top dysregulated SLC gene was *Slc40a1*, which encodes ferroportin, a transmembrane protein that exports iron in cells (Figure 7G). Along with *Slc40a1*, several glucose and small amino acids transporters were upregulated in the absence of *Treml4*, (Figure 7H) while others were downregulated in this genotype (Figure 7I). Importantly, in upregulated SLC genes, oxLDL treatment alone was not sufficient as this effect required abrogation of *Treml4*. In contrast, the downregulation of SLC genes required both oxLDL and *Treml4* abrogation. Because two of the most dysregulated SLC genes were glucose transporters [*Slc2a5* (Glut5), *Slc2a12* (Glut12)], we next investigated if intracellular metabolism was effected in these cells (Figure 7J). Targeted metabolomics analysis revealed that while untreated wt and *Treml4^–/–^* BMDM were similar in respect to their intracellular metabolite pools, *Treml4^–/–^* oxLDL treated macrophages showed lower levels of several glycolytic intermediates and increased accumulation of pyruvate, suggesting a higher rate of flux through glycolysis in these cells. Accordingly, the key pentose phosphate pathway (PPP) intermediates ribose-5-phosphate and ribulose-5-phosphate, produced from carbon diverted from the glycolytic pool, were depleted in *Treml4^–/–^* BMDM after oxLDL suggesting differences in glucose utilization to feed the PPP between the two genotypes. Importantly, levels of TCA intermediates were similar between wt and *Treml4^–/–^* BMDM after oxLDL (Figure 7K) as were sterols and lipid content (Supplementary Figure 8B). In all, our combined transcriptomics and metabolomics analysis in BMDM shows that *Treml4* regulates several key pathways related to cell survival, iron export and glucose metabolism upon atherogenic oxLDL lipid loading.

## Discussion

Here we report for the first time that *Treml4* affects inflammatory programs in macrophages as well as plaque composition during the development of atherosclerosis, although it does not appear to directly affect plaque calcification in our murine model. Thus, despite well-defined differences in atherogenesis between mouse and humans, our results expand on recent genetic association studies and put forward several lines of evidence warranting future investigation into a definitive role for *Treml4* in cardiovascular disease.

High macrophage content is recognized as one of several markers for plaque vulnerability (45, 46). Consistent with its expression on myeloid cells, we have shown that *Treml4* supports accumulation of larger foam cell areas in the aortic sinus. Accordingly, we found higher levels of circulating Ccl2 in *Apoe^–/–^* mice compared to *Apoe^–/–^/Treml4^–/–^* mice. Because inflammatory monocytes are highly dependent on the Ccr2/Ccl2 axis for recruitment to inflammatory sites (47), it is tempting to speculate that Ccl2 might be mediating a specific, and possibly local, effect on monocytic populations in this model. In agreement with this notion, we found that upon *in vitro* treatment with oxLDL, BMDM from *Treml4*^–/^*^–^* mice failed to upregulate *Ccr2* expression to the same extend as wt cells (Figure 4E).

In the periphery, *TREML4* is almost exclusively expressed by Ly6C^low*–*int^ non-classical monocytes (48). In the context of atherosclerosis, these cells have been ascribed a patrolling function and although they can enter the plaques, they do so at a much lower rate than classical monocytes (49). At first, our hypothesis for the higher foam cell area in the *Apoe^–/–^* mice was that Treml4 expression was skewing the peripheral monocytic populations or that it was affecting proliferation and survival in Treml4 positive cells. While this idea is intriguing, our *in vitro* experiments do not support this hypothesis since Treml4 does not affect caspase 3 activation or cell cycle progression even after oxLDL treatment in BMDM. Alternatively, we considered the possibility that *Treml4*-mediated effects on smooth muscle cell (SMC) content in the lesion could contribute to the higher foam cell areas in our mice. Although it is well known that SMC differentiation can contribute to foam cell formation, there is no indication of *Treml4* or *Dap12* expression in smooth muscle cells and we did not detected differences in smooth muscle cell actin (SMA) positive areas after 16 weeks of WD in the aortic sinus (data not shown) or in the BCA (Supplementary Figure S7B). These results make a smooth muscle cell-based explanation unlikely but given our limited analysis of this compartment, we cannot completely rule out this possibility. Nevertheless, our current findings suggest that the dynamics of lesional monocytes might be skewed in the *Apoe^–/–^/Treml4^–/–^* mice and whether this will translate to human lesions remains to be investigated. Moreover, the exact nature of *TREML4*-positive cells within developing lesions, the kinetics of *Treml4* expression during lesion progression and the possibility that macrophage and/or smooth muscle cell survival can be affected *in situ* remains to be investigated.

Our results showing a more complicated plaque composition in *Apoe*^–/^*^–^* mice relative to that of *Apoe*^–/^*^–^*/*Treml4*^–/^*^–^* mice correlates with the alterations in collagen content and maturity and the transcriptional profile of murine inflammatory macrophages that lack *Treml4*. In the BCA, *Treml4* favors the development of cartilaginous metaplasia. This finding is important because murine lesions in the BCA closely resemble several features of advanced human atherosclerosis (50) and studies have shown that cartilaginous metaplasia may progress to ossification (39). In agreement with lower cartilaginous deposition, we found that Mac-3 positive areas are increased in the *Apoe^–/–^/Treml4^–/–^* mice, an inverse relationship that would suggest less advanced plaques at this location as well. Our transcriptome analyses of murine inflammatory macrophages showed that *Treml4* expression regulates important pathways with potential implications in atherosclerotic plaque destabilization. Importantly, we have uncovered *Treml4*-mediated dysregulation of key pathways related to the inflammatory response, osteoclast differentiation, lipid regulation and other metabolically related pathways such as glycolysis in murine macrophages (Figures 1, 7). Although based in part on extrapolation of *in vitro* findings to the complex *in vivo* situation, our findings seemingly support a possible atherogenic role for Treml4 in the context of inflammation.

The finding that our *Apoe^–/–^/Treml4^–/–^* mouse model did not show histological differences in calcium deposition is not surprising for several reasons. First, atherosclerosis is a challenging disease to model in mice. Although the *Apoe* knockout mouse is a widely used model because of its similarities to human comorbidities and lesion characteristics (35), it has limitations. Second, the calcification process in mice and humans is markedly distinct (51), and third, the polypeptide encoded by the murine *Treml4* locus is different from the human TREML4 protein: while murine Treml4 conserves the canonical structure of most TREMs, including its association with DAP12, human TREML4 shares only 39% sequence identity with its murine counterpart (29) and its non-canonical leader sequence does not permit surface expression or association with DAP12. Therefore, the intracellular signaling or even the target cell for human and murine Treml4 is likely to be distinct, and/or the TREML4/Treml4 RNAs themselves may be responsible for some aspects of the immunological impact of this locus. Nevertheless, we show here that human inflammatory macrophages permissive for *TREML4* expression show few differences from those where TREML4 expression is limited, the one exception being specific dysregulated pathways that may skew plaque composition. Accordingly, we find that murine *Treml4* alters plaque characteristics during disease modeling, including cartilaginous deposition, which might ultimately affect atherosclerotic complications such as mineralization.

Our transcriptional profiling of BMDM upon oxLDL loading provides important insight to our understanding of the role of *Treml4* in macrophages and in the murine model of atherosclerosis. First, it confirmed that several inflammatory pathways are dysregulated by *Treml4* expression (Figures 7E,F). Despite being distinct stimuli generally associated with separate disease physiology, oxLDL and LPS both dramatically increased *Treml4* expression and both engage, to different extents, TLR4. Most importantly, oxLDL stimulation permits more direct extrapolation to our atherosclerosis model. For instance, while our *in vitro* analysis of cell cycle did not reveal effects of *Treml4* on cell proliferation or apoptosis, our RNA-seq analysis revealed that upon high oxLDL concentrations *Apoe*^–/^*^–^*/*Treml4*^–/^*^–^* macrophages failed to upregulate genes related to these processes. Second, our data show that, for the most part, the “canonical” macrophage response to an atherogenic stimuli is similar regardless of *Treml4* status (Figures 7B,C). However, comparable to the M1 response, we found that several genes of the “Cellular Response to External Stimulus” pathway failed to be upregulated in the absence of *Treml4*. For example, Matrix metalloproteinase 9 (Mmp9) and SRY-Box Transcription Factor 9 (Sox9), both of which have been implicated in ECM deposition and mineralization (52–54) were lower in oxLDL-stimulated *Treml4^–/–^* macrophages suggesting a mechanism whereby *Treml4* might control plaque composition. Lastly, our oxLDL stimulations uncovered an unexpected mechanism by which *Treml4* might be affecting lesion-associated inflammation. Iron homeostasis and glucose metabolism are key regulators of macrophage function. In macrophages, it has been shown that ferroportin is upregulated by oxLDL (55). Our data confirm those findings but also demonstrate that in the absence of *Treml4*, levels of oxLDL-induced ferroportin are 6-fold the amount seen in wt cells while other iron homeostasis genes remained unaffected (Figure 7G). In retrospect, a relationship between *Treml4* expression and iron metabolism is not entirely surprising, since splenic red pulp macrophages, which function to degrade senescent erythrocytes and recycle heme-associated iron, express high levels of *Treml4*. In fact, a link between iron homeostasis and glycolysis in macrophages is documented, as acute iron depravation results in profound metabolic changes, particularly an increase in glucose metabolism. These changes are associated with anti-inflammatory properties in human macrophages (56). As it has been recently shown that macrophages carefully leverage their metabolism to fuel effector responses, it is tempting to speculate that in oxLDL stimulated cells, high *Treml4* expression limits Slc40a1 expression, maintaining intracellular iron levels that facilitate controlled glycolytic flux. This in turn would permit the diversion of carbon to the PPP for normalization of redox balance, and therefore, macrophage accumulation in disease. Though, we did detect changes in intralesional macrophages, we did not detect any obvious differences in cell viability in the murine lesions or *in vitro* stimulations. Clearly this hypothesis warrants additional studies, given the plethora of potential signals within the lesion, but the prospect of potentially leveraging iron balance and metabolic control in patients with high TREML4 expression is intriguing.

Overall, our study suggests that Treml4 is proatherogenic by accelerating lesion burden and complexity, increasing macrophage content and circulating Ccl2 levels as well as extracellular matrix content. Additionally, we put forward several dysregulated pathways in oxLDL-treated cells including gene candidates *Slc40a1*, *Sox9*, and *Mmp9*, that warrant future investigation in human disease and finally, we suggest a possible interplay between iron homeostasis and glucose metabolism that while it still need to be verified in human disease, it is an attractive target for anti-inflammatory modulation. While we do not believe TREML4 is causative for the initiation of calcified plaques, our data suggest that TREML4 could predispose the transition of soft plaques to calcified ones by skewing lesion composition. Further studies are needed to address possible *TREML4* expression in other tissues, identify specific ligands for both the human and mouse isoforms, and to test whether a soluble form of the receptor exists which can perhaps reconcile the human and mouse findings.

## Data Availability Statement

The dataset generated for this study can be found in the Gene Expression Omnibus (GEO) under superseries GSE145384. Other raw data supporting the conclusions of this article will be made available by the authors, without undue reservation, to any qualified researcher.

## Ethics Statement

The studies involving human participants were reviewed and approved by the Frederick National Laboratory for Cancer Research Institutional Review Board (IRB approval number 16-003). Human blood was obtained from healthy volunteers recruited through the National Cancer Institute-Frederick Research Donor Program in accordance with the Declaration of Helsinki and provided written informed consent under IRB-approved protocol. Ethics approval for the animal experiments detailed in this manuscript was received from the Institutional Animal Care and Use Committee (Permit Number: 000386) at the NCI-Frederick.

## Author Contributions

MG-C planned and conducted the experiments, analyzed the data, and wrote the manuscript. LG, MK, CC, EP, and KB conducted the experiments and animal work and edited the manuscript. SS, JB, and FE analyzed the NGS data and edited the manuscript. FK, AF, and LB contributed to experimental design, edited the manuscript, and contributed the reagents. DM planned and supervised the experiments and edited the manuscript.

## Conflict of Interest

MK and FE were employed by Leidos Biomedical Research, Inc. LG, CC, FK, and AF were employed by CVPath Institute, Inc. The remaining authors declare that the research was conducted in the absence of any commercial or financial relationships that could be construed as a potential conflict of interest.
