# Supplementary material for: TREML4 Promotes Inflammatory Programs in Human and Murine Macrophages and Alters Atherosclerosis Lesion Composition in the Apolipoprotein E Deficient Mouse
Source: Front Immunol. 2020 Mar 30;11:397. doi: 10.3389/fimmu.2020.00397 (PMC7133789; doi:10.3389/fimmu.2020.00397)
Supplement: Supplementary file 1 [file Data_Sheet_1.PDF]

Supplemental Methods

Histological analyses of atherosclerosis progression and calcification

Mice were perfused *in situ* after anesthesia with 1X PBS by inserting a 20-gauge hypodermic needle with an IV line attached to lactated ringers into the heart left apex. A nick was made in the right atrium to allow perfusate to flow by physiological pressure. After 15 minutes, 4% Paraformaldehyde (PFA) was used to fix-perfuse until the liver was pale in color (approximately 15 minutes). For harvest of fresh, non-fixed tissue, only cold PBS was used for the perfusions. When necessary, tissues were further fixed by submersion in 10% formalin. Hearts were mounted in paraffin or flash frozen in OCT for sectioning and 8-10 µm sections were collected of the aortic sinus.

Polarized light microscopy

Images of picrosirius red staining under polarized light were acquired as previously described[33] using an Olympus BX51 microscope with an Olympus DP73 camera. The collagen type I (yellow-red birefringence) and III (green birefringence) signals were quantified on HALO platform (Indica Labs, Corrales, NM).

Mouse bone marrow isolation, culture and treatments

Bone marrow cells were flushed from femurs and tibias with cold PBS. Cell suspensions were grown for 6-7 days in DMEM, 10% FBS, and 50 ng/ml MCSF (Peprotech, Rocky Hills, NJ).

TaqMan Gene Expression Assay probes:

Mouse: *Treml4* (Mm00553947_m1), *Cd36* (AssayID: Mm00432403_m1) and *Gapdh* (AssayID: Mm99999915_g1).

Human: HPRT (Hs02800695_m1), *MRC1* (Hs00267207_m1), *TNF* (Hs00174128_m1) and *IRG1* (*ACOD1*) (Hs00985781_m1).

Serum cholesterol analysis

After overnight fasting, blood was collected into serum separator tubes and saved at -80 °C for further analysis. Cholesterol levels were measured using the Cholesterol-E colorimetric kit (Wako Diagnostics, Richmond, VA) according to manufacturer's instructions.

Serum cytokine analysis

Serum levels of MCP-1, IL10, IL6, IL12, and TNF were measured using the Becton-Dickinson Multiplexed Cytometric Bead Array (Becton-Dickinson Bioscience, Franklin Lakes, NJ) according to manufacturer's instructions. Acquisition was performed using a Becton-Dickinson LSRII flow cytometer.

Isolation and treatment of peripheral blood human monocytes

Blood from donors was drawn into PaxGene tubes and PBMCs were prepared by Ficoll gradient. PBMCs were differentiated into macrophages with G-CSF for seven days and either left unstimulated or treated with 100 ng/ul LPS for 8 hours. Cells were harvested, and RNA isolation was performed using PAXgene Blood RNA Kit IVD (QIAGEN, USA) or the Rneasy Mini Kit (QIAGEN). Genotyping for rs2803495 and rs2803496 was done using TaqMan SNP Genotyping Assays (Life Technologies, Carlsbad, CA) on an Applied Biosystems 7900HT Fast Real-Time PCR System (Life Technologies).

Gene ontology pathway and process enrichment analysis

Enrichment terms were determined using Metascape (Tripathi et. al. 2015) using default parameters. Significant differentially expressed genes for comparisons of interest were used as input and murine or human whole genome was used as background as appropriate. RNASeq of oxLDL loaded cells was analyzed using Partek Genomics Suites version 7 build-in pipeline. In short, aligned BAM files were imported and mRNA quantification was performed using mm10 RefSeq Transcripts (2018-11-08), where genes with less than 3 reads were filtered out. Rpkm gene counts were generated and used thereafter. Differentially expressed genes (DEG) were identified by build-in ANOVA model, taking the genotype and treatments as factors and a significant p value of 0.05 was used for generation of DEG lists. All related plots were generated using Partek and enrichment analysis was performed using Cytoscape.

Treml4 promotes inflammatory programs in human and murine macrophages and alters atherosclerosis lesion composition in the Apolipoprotein E deficient mouse

Supplemental figure 1

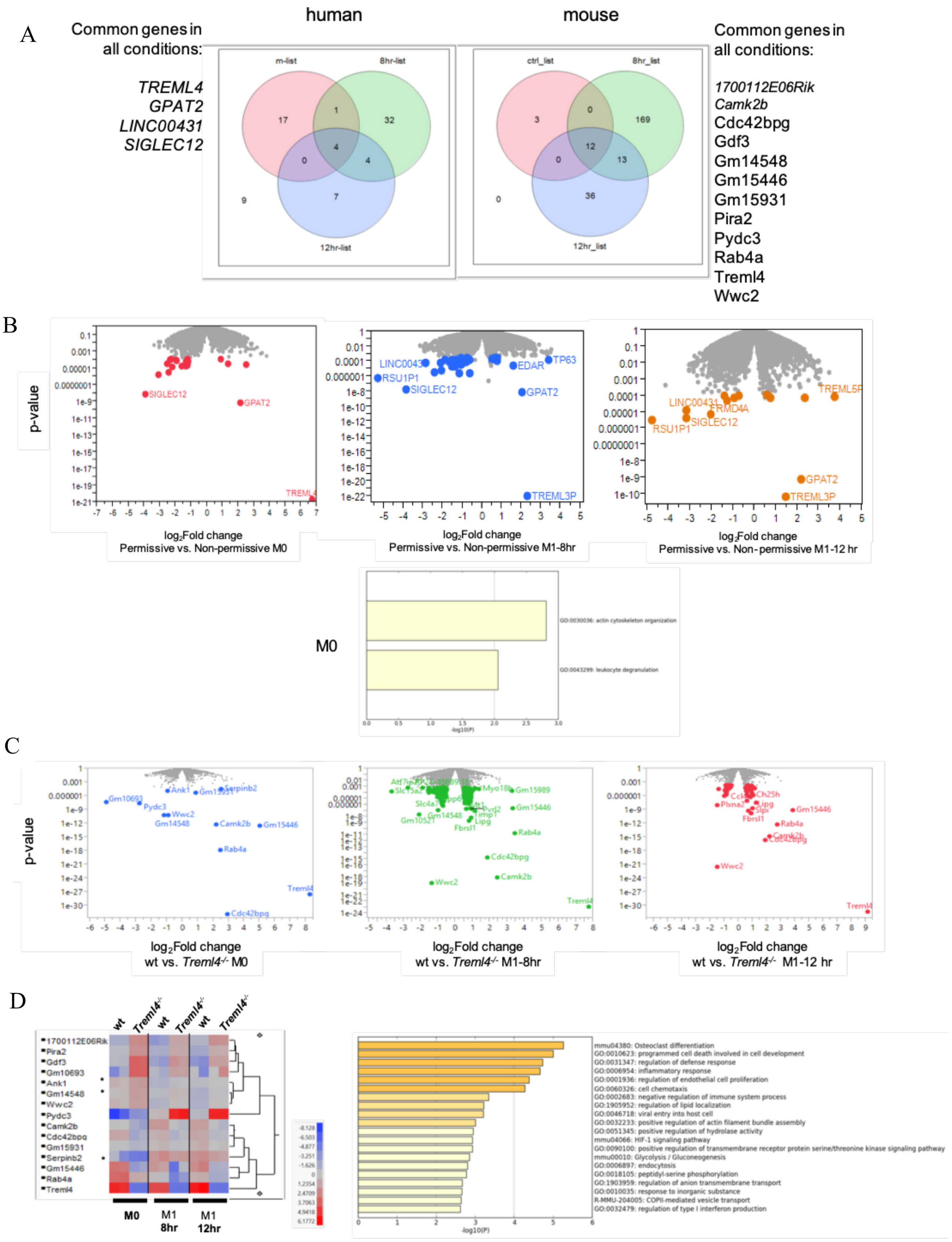

Supplemental figure 1- Transcription profiling of human and murine Treml4 positive M1 macrophages (A) Venn diagrams of differentially expressed genes in both human and murine treatment groups. (B) Volcano plots of DEG in M0, M1-8hr and M1-12hr in permissive human macrophages. Colored dots represent genes that passed FDR and fold-change filters. (C) Volcano plots of DEG in M0, M1-8hr and M1-12hr wild type murine macrophages compared to *Treml4*$^{-/-}$. Colored dots represent genes that passed FDR and fold-change filters. (D) Heatmap of expression values of genes constitutively dysregulated in all conditions in murine macrophages and Enrichment ontology graph of M1-8hr genes.

Supplemental figure 2

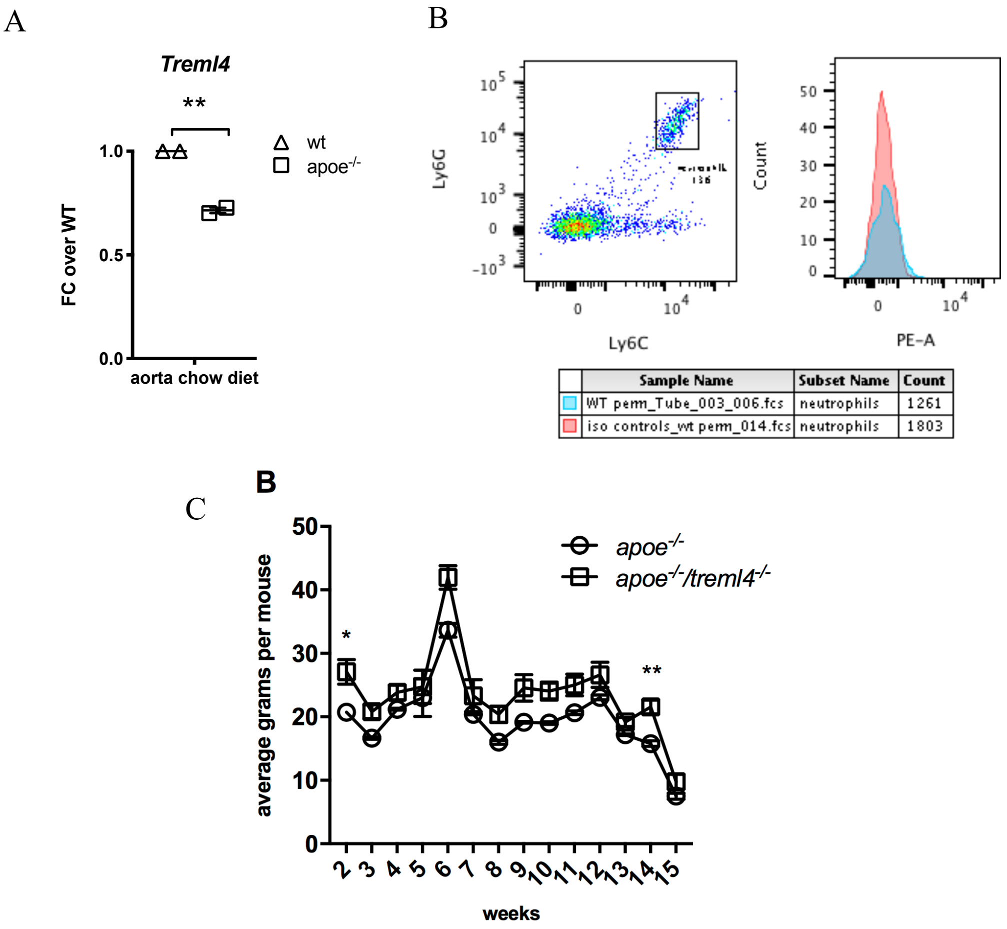

Supplemental figure 2- *Treml4* expression and food consumption in chow diet fed mice over 16 weeks of WD.

(A) *Treml4* expression in murine whole aorta lysates in 26-week-old mice. **P<0.01, n=2 per genotype (Student's t-test). (B) Whole blood flow cytometry for Treml4 expression in Ly6C/Ly6G double-positive cells (neutrophils). (C) Food consumption was monitored per cage and amount consumed was averaged by number of mice, n=14 per genotype (Two-way ANOVA with Sidak's multiple comparison test).

Supplemental figure 3

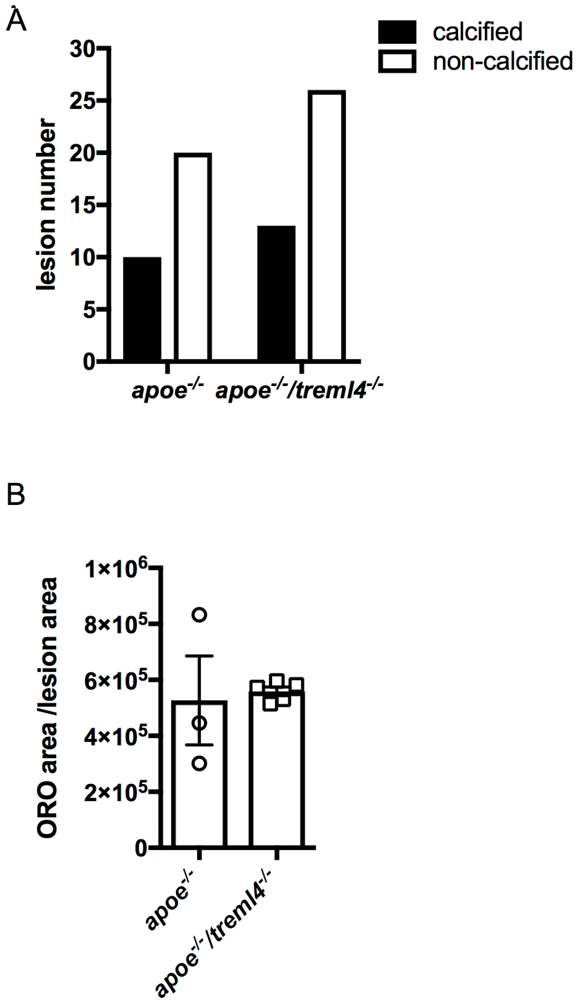

Supplemental figure 3- *Additional features in 16 weeks WD aortic sinus lesions.*

(A) Frequency of lesions that were positive for atherosclerotic calcification with in the aortic sinus. N= 30 lesions for *Apoe*$^{-/-}$ and n=23 for *Apoe*$^{-/-}$/*Treml4*$^{-/-}$. Data were analyzed by Fisher's exact test. (B) ORO-positive areas normalized to lesion areas, n= 3-5 (Student's t-test).

Supplemental figure 4

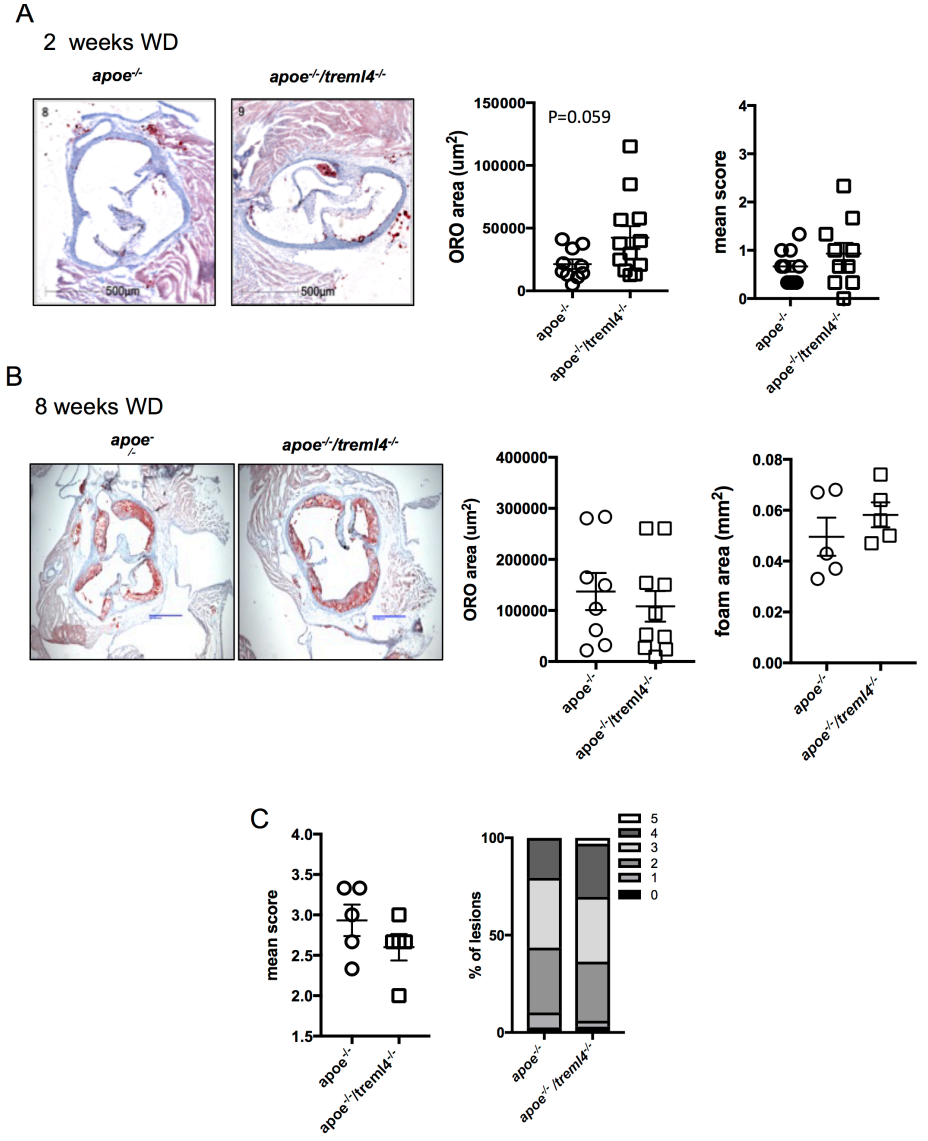

Supplemental figure 4- *Treml4* effect on atherosclerosis development after 2 and 8 weeks of WD. (A) Representative images of ORO-stained aortic sinus, ORO-positive and mean histological score of aortic sinus lesions after 2 weeks of WD, n=10 for *Apoe*$^{-/-}$ and n=12 for *Apoe*$^{-/-}$/*Treml4*$^{-/-}$ (Student's t-test). (B) Representative images of ORO-stained aortic sinus and ORO-positive and foam cell area quantifications in aortic sinus lesions after 8 weeks WD, n=8-9 mice per genotype (Student's t-test). (C) Mean lesion score and frequency of lesion types after 8 weeks WD, n-5 mice per genotype (Student's t-test).

Supplemental figure 5

A

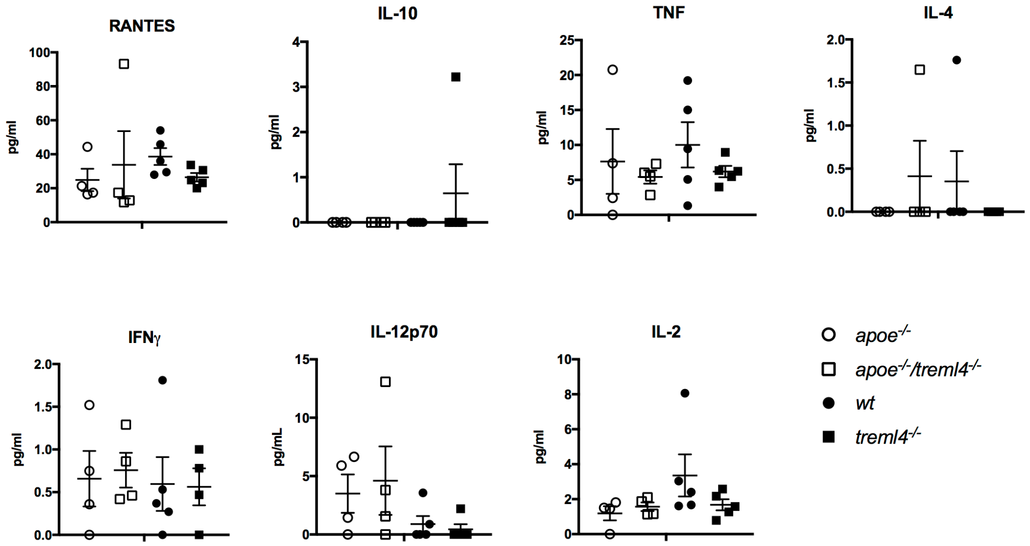

Supplemental figure 5- *Serum cytokines after WD.*

(A) Analysis of the indicated cytokines in fasting serum from mice fed WD for 16 weeks. Data represent mean ± SEM of individual mice, n= 4 for *Apoe*$^{-/-}$ and *Apoe*$^{-/-}$/*Treml4*$^{-/-}$ mice, n=5 for wt and *Treml4*$^{-/-}$ mice (Two-way ANOVA with Tukey post-test).

Supplemental figure 6

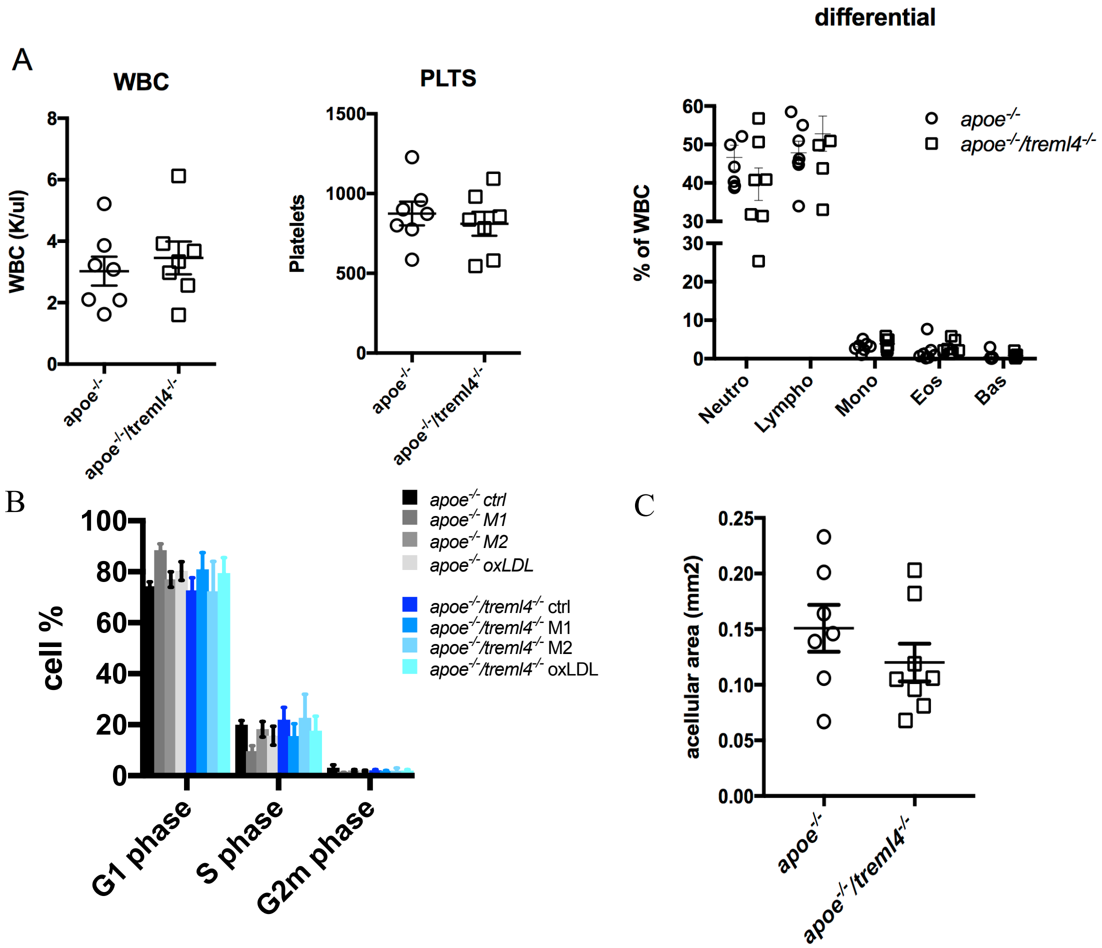

Supplemental figure 6- *Blood cell numbers after 16 weeks HFD*

(A) White cells and platelets numbers and differential blood counts in EDTA-blood samples of mice fed WD for 16 weeks, n= 7 mice per genotype. (B) Cell cycle analysis of BMDM treated with LPS (M1), IL4 (M2), oxLDL of left untreated overnight. C) Morphometric analyses of acellular areas in lesions after 16 weeks of WD, n= 7 *Apoe*$^{-/-}$, n=8 *Apoe*$^{-/-}$/*Treml4*$^{-/-}$ (Student's t-test).

Supplemental figure 7

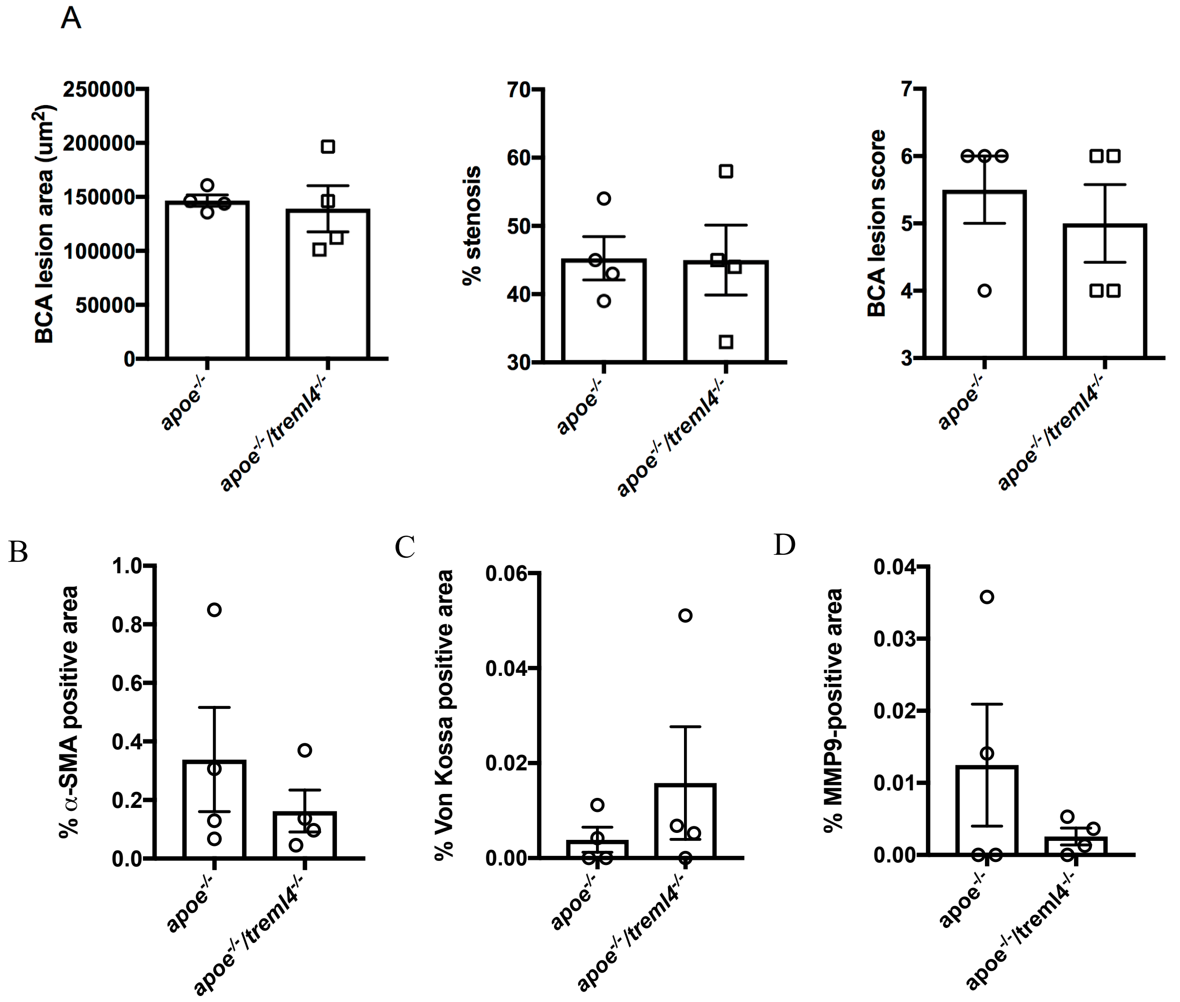

Supplemental figure 7 - *Atherosclerosis development in the brachiocephalic artery after western diet.*

(A) Lesion area, percentage of stenosis and lesion score of atherosclerotic lesions in the BCA after 16 weeks of WD, n=4 per genotype (Student's t-test). (B) Immunostaining for smooth muscle cell actin in the BCA, (C) VonKossa staining and (D) Immunostaining of Mmp-9. Data represents percentage of positive area/total lesion area.

Supplemental figure 8

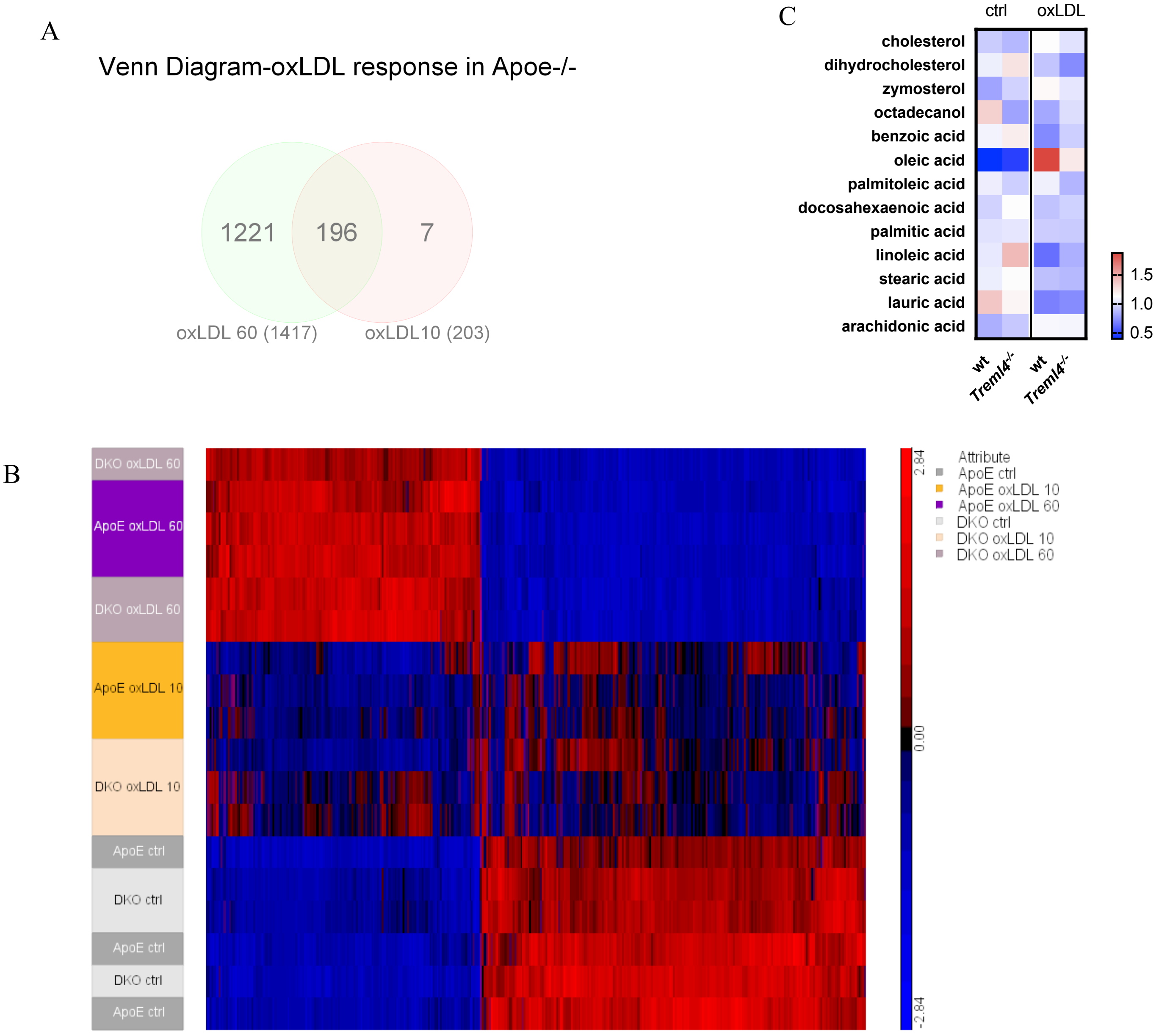

Supplemental figure 8 – *Transcriptomics and metabolomics analysis of oxLDL-loaded macrophages*

(A) Venn diagram of DEG after oxLDL in *Apoe*$^{-/-}$ macrophages. (B) Hierarchical clustering of 1009 genes differentially expressed in both genotypes after oxLDL60. (C) Lipid metabolites detected in BMDM after oxLDL-loading.

Supplemental table 1- RNA-seq human DEG list

Treml4 promotes inflammatory programs in human and murine macrophages and alters atherosclerosis lesion composition in the Apolipoprotein E deficient mouse

gene symbol	M0-list	PBMC_list	M1_8hr-list	M1_12hr-list	P-log2FC	P-pval	P-FDR-pval	M-log2FC	M-pval	M-FDR-pval	8hr-log2FC	8hr-pval	8hr-FDR-pval	12hr-log2FC	12hr-pval	12hr-FDR-pval
TREML4	1	1	1	1	7.6776158	1.10E-27	2.43E-23	6.73669607	2.01E-21	4.44E-17	4.01106139	2.18E-80	4.81E-76	3.75394765	2.65E-71	5.85E-67
AFAP1	1	1	0	0	-1.2223314	0.0000696	0.1280118	-1.1768913	0.0000571	0.08345597	-0.7363895	0.01207826	0.57959781	-1.0097864	0.0005724	0.2509464
GPAT2	1	0	1	1	1.39983727	0.00016867	0.23266834	2.12136822	7.53E-10	8.3097E-06	2.02611783	8.14E-09	5.9886E-05	2.20079654	7.84E-10	5.7679E-06
LINC00431	1	0	1	1	-2.5071127	0.00178327	0.57041444	-3.0970125	0.00000172	0.00949053	-2.8631526	0.0000726	0.0784046	-3.1599383	0.000013	0.040989
SIGLEC12	1	0	1	1	-2.3955971	0.00063292	0.40564172	-3.9367306	7.28E-09	5.3559E-05	-3.8687907	1.97E-08	0.0001087	-3.1883139	0.00000439	0.01937834
AP001434.2	1	0	1	0	0.06377406	0.91918065	0.99656968	-1.2249669	0.0000224	0.05493227	-1.0853689	0.0001241	0.10020234	-0.6938867	0.01581706	0.70382706
AQP1	1	0	0	0	-0.2156979	0.60244207	0.99656968	-1.9562129	0.0000979	0.11372373	0.16716291	0.80377165	0.99445439	-0.4435973	0.52454276	0.98116344
ATF3	1	0	0	0	-0.1529647	0.67574408	0.99656968	1.33520055	0.0000309	0.05501985	0.21418666	0.50169265	0.98145695	0.02587901	0.93530303	0.9955032
CDC42EP1	1	0	0	0	-1.0207353	0.09106611	0.99656968	-2.3051758	0.00013049	0.13387466	-2.0225659	0.00078057	0.22373946	-2.203379	0.00024972	0.20038641
CHI3L1	1	0	0	0	0.24664423	0.68354093	0.99656968	2.53036791	0.0000293	0.05501985	2.01664144	0.00085907	0.2340793	2.10154373	0.00051376	0.24535438
IGLON5	1	0	0	0	0.98881095	0.39243736	0.99656968	-2.5408581	0.0000329	0.05501985	-0.9588473	0.17317195	0.92717461	-1.9712018	0.02985855	0.76710327
KCNJ15	1	0	0	0	-0.0603593	0.84553532	0.99656968	-1.3333314	0.0000187	0.05159096	-0.725398	0.01941319	0.66223882	-0.5872315	0.05844356	0.83305709
MRC2	1	0	0	0	-0.5984909	0.06931823	0.99656968	-1.3733206	0.0000261	0.05501985	-0.8293125	0.01097983	0.56298202	-0.9495625	0.00368873	0.44621771
MYLK2	1	0	0	0	1.24482733	0.29494315	0.99656968	-2.3473849	0.0000886	0.10863837	-0.8607735	0.39472462	0.97979686	-0.1597387	0.88005074	0.9955032
NR4A3	1	0	0	0	0.29896998	0.64574679	0.99656968	0.9020878	0.00013344	0.13387466	-0.2635621	0.2538479	0.95066902	-0.3839828	0.09656376	0.86749516
NXPH4	1	0	0	0	0.27300515	0.61706181	0.99656968	-2.2164932	0.0000136	0.0500276	-1.0849352	0.03324337	0.74302303	-0.6563351	0.1972018	0.92142572
PTPRB	1	0	0	0	-0.1037173	0.87789463	0.99656968	-1.9258518	0.0000833	0.1081479	-1.2361238	0.01378865	0.59733119	-1.2938494	0.00790218	0.59934347
PTPRF	1	0	0	0	-0.4539186	0.40708246	0.99656968	-2.4249451	0.00000346	0.01527313	-1.1155397	0.03339067	0.74302303	-0.5036887	0.33354464	0.94775258
SH3PXD2B	1	0	0	0	-0.412518	0.31643747	0.99656968	-1.5882887	0.0000185	0.05159096	-0.7983598	0.03127366	0.73017313	-0.4313495	0.24464712	0.93100306
SPATA20P1	1	0	0	0	0.36508014	0.47613148	0.99656968	-1.2518082	0.0000349	0.05501985	-0.9330417	0.00186035	0.32398386	-0.6726358	0.02568433	0.75592179
SRCRB4D	1	0	0	0	0.7155343	0.11678892	0.99656968	-1.221536	0.00013059	0.13387466	-0.8916027	0.02004265	0.66713241	-0.597533	0.11286034	0.87523953
ZNF462	1	0	0	0	-1.0809653	0.16426666	0.99656968	-1.3455147	0.0000605	0.08345597	-0.7231908	0.02194826	0.67450909	-0.7310587	0.01877155	0.7118673
TREML3P	0	1	1	1	1.4343493	1.80E-11	1.3243E-07	0.8244881	0.05915795	0.99990292	2.31438744	1.07E-22	1.18E-18	1.46132952	6.71E-11	7.4048E-07
TREML5P	0	1	0	1	4.48276223	1.07E-22	1.18E-18	2.18651787	0.0247432	0.99990292	2.49909367	0.01267574	0.58651189	3.736568	0.00008	0.13582154
A4GALT	0	1	0	0	3.42405494	0.00000501	0.01842929	0.30792749	0.64493577	0.99990292	0.25957952	0.69637483	0.98656569	0.02957793	0.96452982	0.9955032
C1orf226	0	1	0	0	3.26285465	0.0000255	0.05628105	1.42220833	0.05629583	0.99990292	0.49049384	0.52735241	0.98145695	1.02849971	0.17847848	0.91091383
C4BPA	0	1	0	0	-4.0143555	0.0000436	0.08748142	-0.7311975	0.53056499	0.99990292	-0.9379148	0.37229054	0.97841581	0.13891258	0.90203319	0.9955032
EEF1A2	0	1	0	0	-2.0877228	0.0000851	0.14448016	-0.7147351	0.11427438	0.99990292	-0.2923736	0.51798177	0.98145695	-0.5561513	0.21474053	0.93100306
IFI27	0	1	0	0	3.25697192	0.00000205	0.00904911	1.29091194	0.0601581	0.99990292	-0.1831483	0.78881287	0.99445439	-0.1118403	0.87006985	0.9955032
KB-226F1.2	0	1	0	0	4.87480769	0.00000919	0.02253694	3.8300806	0.00031171	0.24570698	3.49448702	0.00141543	0.2835185	2.69562824	0.01530624	0.69867899
LINC00680	0	1	0	0	-1.4949907	2.99E-07	0.00164981	-0.5284471	0.04056724	0.99990292	-0.2381106	0.36990974	0.97803949	-0.541713	0.04274947	0.80574174
PCGF3	0	1	0	0	-0.5477377	0.00000783	0.02253694	-0.2012192	0.07781365	0.99990292	-0.1542709	0.17978217	0.93623686	-0.1531751	0.182221	0.91271159
PVRL2	0	1	0	0	-2.0147952	0.00000914	0.02253694	-0.2731719	0.54421832	0.99990292	-0.2237368	0.61930192	0.98145695	-0.0594564	0.89496091	0.9955032
COL7A1	0	0	1	1	-0.2925796	0.30504325	0.99656968	-1.0287655	0.00018419	0.17674841	-1.3660215	0.0000203	0.03733678	-1.2645359	0.0000473	0.13049479
FRMD4A	0	0	1	1	-0.0128618	0.97794511	0.99939567	-0.7899489	0.07891175	0.99990292	-2.0648189	0.00000569	0.0125584	-2.034467	0.00000681	0.02505059
RSU1P1	0	0	1	1	-3.0731425	0.00722764	0.87649046	-3.0591845	0.00285422	0.59429666	-5.3221294	6.29E-07	0.00231378	-4.7820886	0.00000302	0.01666361
ASNS	0	0	1	0	0.27654958	0.19307871	0.99656968	0.38018009	0.06943826	0.99990292	0.80005215	0.00011581	0.09831187	0.78966293	0.0001824	0.18088748
BCL2L11	0	0	1	0	-0.1111818	0.55924252	0.99656968	0.23208765	0.21183437	0.99990292	0.67230008	0.00031564	0.1482232	0.57242658	0.00206744	0.38473908
BLZF1	0	0	1	0	-0.1520241	0.63376298	0.99656968	-0.0270241	0.9267437	0.99990292	-1.0595107	0.00030244	0.14510963	-0.6623289	0.02345697	0.75592179
CDCA7L	0	0	1	0	0.10132	0.58724821	0.99656968	0.23199757	0.19225733	0.99990292	0.69448356	0.00014187	0.10020234	0.58778802	0.00136308	0.33441475
CREB5	0	0	1	0	0.07799987	0.77898609	0.99656968	0.26355805	0.34986522	0.99990292	-1.2070046	0.0000453	0.06248852	-0.6283213	0.04023632	0.80228319
CRYBB1	0	0	1	0	0.64532705	0.22752726	0.99656968	-0.4894195	0.03959315	0.99990292	-1.0858632	0.0000409	0.06018026	-0.1062904	0.75725928	0.9950863
DHX32	0	0	1	0	0.23351792	0.22162384	0.99656968	0.16813031	0.30226347	0.99990292	0.62185251	0.00026744	0.13553651	0.46583584	0.00584813	0.5400589
DIRC2	0	0	1	0	0.0611211	0.80745604	0.99656968	0.00037426	0.99875034	0.99990292	-0.9611571	0.0000873	0.08758174	-0.655218	0.00684221	0.56867649
EDAR	0	0	1	0	0.67562226	0.08657896	0.99656968	0.65718718	0.05582373	0.99990292	1.60923384	0.00003	0.047295	0.68134386	0.06591446	0.84038778
GAL3ST4	0	0	1	0	-0.3050723	0.32480742	0.99656968	-1.0766188	0.00039344	0.248102	-1.1994108	0.00013516	0.10020234	0.0806725	0.79972465	0.9955032
HTRA1	0	0	1	0	0.11798466	0.79085663	0.99656968	-0.9897564	0.01369004	0.99990292	-1.5122277	0.00017815	0.10626769	-1.1690105	0.00430079	0.47148409
IVNS1ABP	0	0	1	0	0.15100843	0.47063542	0.99656968	-0.0464603	0.82317584	0.99990292	0.76670229	0.00024203	0.12825563	0.55682122	0.00757715	0.59795494
MED25	0	0	1	0	-0.205019	0.24393312	0.99656968	-0.3024817	0.08576515	0.99990292	-0.6652606	0.00015768	0.10020234	-0.5114414	0.0036859	0.44621771
MERTK	0	0	1	0	0.0447676	0.90582487	0.99656968	-0.6300098	0.06671493	0.99990292	-1.3157031	0.00016016	0.10020234	-0.5213489	0.13506003	0.88519871
MGST3	0	0	1	0	-0.3008632	0.07188522	0.99656968	-0.395115	0.01405921	0.99990292	-0.6143632	0.00014646	0.10020234	-0.3664637	0.02413528	0.75592179
MMP2	0	0	1	0	-1.3281375	0.10256056	0.99656968	-1.6143293	0.00222309	0.53332484	-2.1207819	0.0000611	0.07318279	-1.7158591	0.00118954	0.33441475
OSBPL3	0	0	1	0	-0.0645419	0.66863558	0.99656968	-0.013996	0.91381733	0.99990292	0.50789572	0.00019645	0.11117446	0.32395077	0.0154115	0.69867899
OSM	0	0	1	0	0.09256941	0.76314231	0.99656968	-0.5068165	0.08145977	0.99990292	-1.048731	0.00030013	0.14510963	-0.5820053	0.04455534	0.80875958
PDK1	0	0	1	0	0.08726611	0.67731251	0.99656968	0.28057089	0.14907545	0.99990292	0.73024558	0.00024406	0.12825563	0.21748992	0.27466122	0.93295223
PHACTR4	0	0	1	0	0.11934316	0.42174304	0.99656968	-0.0287994	0.83086225	0.99990292	-0.6360523	0.00000225	0.00709425	-0.4333019	0.00129875	0.33441475
PTPRU	0	0	1	0	0.16439607	0.74103586	0.99656968	-0.4514273	0.21032468	0.99990292	-1.5018645	0.0000181	0.03631683	-1.2695049	0.0002511	0.20038641
RBMS2	0	0	1	0	-0.0946741	0.79005075	0.99656968	-0.1736352	0.60417371	0.99990292	-1.3277763	0.0000746	0.0784046	-1.150001	0.00060388	0.25167811
RP11-1036E20.7	0	0	1	0	1.60497681	0.02076743	0.99656968	0.05071291	0.86852252	0.99990292	-1.030698	0.00005	0.06491471	-0.8951633	0.00052248	0.24535438
TGFBI	0	0	1	0	0.29024429	0.23734814	0.99656968	-0.6290022	0.01021694	0.91315772	-1.1489559	0.00000272	0.00750414	-0.9016984	0.00023215	0.20038641
TMEM110	0	0	1	0	-0.4612925	0.07711004	0.99656968	-0.2850727	0.16772433	0.99990292	-0.7332767	0.0002702	0.13553651	-0.5650997	0.00529157	0.51449484
TMEM171	0	0	1	0	-1.1784516	0.04625899	0.99656968	-0.0837135	0.87917089	0.99990292	-1.8868876	0.00015265	0.10020234	-1.5535555	0.00182131	0.3589121
TP63	0	0	1	0	1.42365621	0.12804006	0.99656968	0.03000095	0.95496693	0.99990292	3.38476542	0.00013974	0.10020234	0.68719557	0.37536096	0.95788147
TPTEP1	0	0	1	0	-1.1972512	0.00473763	0.78033058	-1.3227579	0.00180582	0.5118472	-1.6593589	0.0000962	0.088284	-1.3264982	0.00179062	0.3589121
UNC5C	0	0	1	0	0.17906358	0.85814465	0.99656968	-2.2000008	0.00888631	0.86472058	-2.4310959	0.00000357	0.00875483	-1.9845535	0.0001553	0.18088748
VIM-AS1	0	0	1	0	0.05948691	0.7802805	0.99656968	-0.491116	0.01907316	0.99990292	-0.8157697	0.0000938	0.088284	-0.7883513	0.00019492	0.18088748
ZNF467	0	0	1	0	0.10430264	0.70052934	0.99656968	-0.2036048	0.453409	0.99990292	-1.0429431	0.00016344	0.10020234	-0.8801395	0.0013094	0.33441475
ZNF516	0	0	1	0	0.02406514	0.89663596	0.99656968	-0.1456891	0.42658852	0.99990292	-0.6904585	0.00019643	0.11117446	-0.5448654	0.00341795	0.44621771
AP000347.4	0	0	0	1	-0.0555396	0.78423225	0.99656968	-0.4999878	0.03785756	0.99990292	0.17418409	0.67628592	0.98333894	-1.3943063	0.0000934	0.13742876
CHD1L	0	0	0	1	0.20962382	0.19128196	0.99656968	0.31041305	0.03042576	0.99990292	0.45513741	0.00268232	0.38592734	0.58751059	0.00010319	0.14234416
CHIT1	0	0	0	1	0.59841472	0.31382763	0.99656968	1.14659301	0.05462582	0.99990292	2.1117466	0.00045295	0.18862376	2.37070543	0.0000766	0.13582154
CLUHP3	0	0	0	1	-0.2721086	0.16777706	0.99656968	0.17534389	0.35743628	0.99990292	-0.2862295	0.12802345	0.88931878	-0.7379399	0.0000902	0.13742876
EIF3C	0	0	0	1	0.18908079	0.33952918	0.99656968	0.43159927	0.02357164	0.99990292	0.60365337	0.00164049	0.30426265	0.76717585	0.0000703	0.13582154
SPTBN5	0	0	0	1	-0.1496671	0.54140226	0.99656968	-0.1474345	0.52217194	0.99990292	-0.7283941	0.0018146	0.3229847	-0.9248989	0.0000695	0.13582154

Supplemental table 2- Enrichment analysis human M1 macrophages in Metascape

GroupID	Category	Term	Description	LogP	Log(q-value)	InTerm_InList	Symbols
1_Summary	GO Biological Processes	GO:0042475	odontogenesis of dentin-containing tooth	-5.007413	-0.6960407	4/87	HTRA1,TP63,BCL2L11,EDAR,MERTK,ASNS
1_Member	GO Biological Processes	GO:0042475	odontogenesis of dentin-containing tooth	-5.007413	-0.6960407	4/87	HTRA1,TP63,BCL2L11,EDAR
1_Member	GO Biological Processes	GO:0042476	odontogenesis	-4.4003432	-0.3900008	4/124	HTRA1,TP63,BCL2L11,EDAR
1_Member	GO Biological Processes	GO:0048608	reproductive structure development	-2.3889541	0	4/425	HTRA1,TP63,BCL2L11,MERTK
1_Member	GO Biological Processes	GO:0061458	reproductive system development	-2.3781187	0	4/428	HTRA1,TP63,BCL2L11,MERTK
1_Member	GO Biological Processes	GO:0048732	gland development	-2.3602501	0	4/433	ASNS,TP63,BCL2L11,EDAR
1_Member	GO Biological Processes	GO:0007548	sex differentiation	-2.0879493	0	3/268	TP63,BCL2L11,MERTK
2_Summary	Reactome Gene Sets	R-HSA-147422	Degradation of the extracellular matrix	-2.8797083	0	3/140	COL7A1,MMP2,HTRA1,TGFBI,ZNF516,TP63
2_Member	Reactome Gene Sets	R-HSA-147422	Degradation of the extracellular matrix	-2.8797083	0	3/140	COL7A1,MMP2,HTRA1
2_Member	GO Biological Processes	GO:0030198	extracellular matrix organization	-2.7008997	0	4/348	COL7A1,MMP2,HTRA1,TGFBI
2_Member	GO Biological Processes	GO:0043062	extracellular structure organization	-2.4827585	0	4/400	COL7A1,MMP2,HTRA1,TGFBI
2_Member	GO Biological Processes	GO:0061448	connective tissue development	-2.1377279	0	3/257	COL7A1,TGFBI,ZNF516
2_Member	GO Biological Processes	GO:0001501	skeletal system development	-2.1264012	0	4/505	COL7A1,MMP2,TGFBI,TP63
3_Summary	GO Biological Processes	GO:0035148	tube formation	-2.844584	0	3/144	BCL2L11,EDAR,PHACTR4
3_Member	GO Biological Processes	GO:0035148	tube formation	-2.844584	0	3/144	BCL2L11,EDAR,PHACTR4
4_Summary	GO Biological Processes	GO:0048598	embryonic morphogenesis	-2.7469534	0	5/573	COL7A1,MMP2,TP63,BCL2L11,PHACTR4
4_Member	GO Biological Processes	GO:0048598	embryonic morphogenesis	-2.7469534	0	5/573	COL7A1,MMP2,TP63,BCL2L11,PHACTR4
5_Summary	GO Biological Processes	GO:0097193	intrinsic apoptotic signaling pathway	-2.0070767	0	3/287	PDK1,BCL2L11,IVNS1ABP
5_Member	GO Biological Processes	GO:0097193	intrinsic apoptotic signaling pathway	-2.0070767	0	3/287	PDK1,BCL2L11,IVNS1ABP

Supplemental table 3- RNA-seq mouse DEG list 5%FDR, 1.4log2 FC

gene symbol	M0	M1_ 8hr	M2_ 12hr	M0-log2FC	M0-pval	M1_8hr-log2FC	M1_8hr-pval	M0_12hr-log2FC	M0_12hr-pval	FDR-M0-pval	FDR-M1_8hr-pval	FDR-M1_12hr-pval
1700112E06Rik	1	1	1	3.878504335	1.14E-61	3.976937508	2.40E-40	3.054650036	7.11E-44	6.22E-58	9.82E-37	2.91E-40
Camk2b	1	1	1	-2.218742971	6.12E-13	-2.427037657	8.92E-19	-2.211576832	1.43E-15	1.25E-09	2.09E-15	2.93E-12
Cdc42bpg	1	1	1	-2.943262253	1.22E-32	-1.881287992	1.69E-15	-1.920681133	2.29E-16	4.99E-29	3.46E-12	5.35E-13
Gdf3	1	1	1	4.431008241	9.93E-98	4.314256808	2.10E-63	4.838845844	3.79E-60	1.63E-93	1.15E-59	2.07E-56
Gm14548	1	1	1	1.184144527	5.09E-11	1.010543703	0.000000118	1.01071475	0.000000895	9.26E-08	0.000137942	0.000770925
Gm15446	1	1	1	-5.044054737	2.96E-13	-3.322539447	0.000000261	-3.853953114	6.23E-10	6.92E-10	0.000237307	8.50E-07
Gm15931	1	1	1	-0.88618239	0.00000506	-0.976244433	0.000000183	-0.790615675	0.0000192	0.006370151	0.000199665	0.0098196
Pira2	1	1	1	2.53410646	5.56E-74	2.536992737	7.97E-64	2.886718122	1.55E-74	4.55E-70	6.52E-60	1.27E-70
Pydc3	1	1	1	2.802769239	0.000000023	4.973041367	2.45E-218	6.201898877	2.93E-301	3.42E-05	4.01E-214	4.80E-297
Rab4a	1	1	1	-2.488220952	1.43E-18	-3.441841693	1.80E-11	-2.734320405	5.60E-13	3.90E-15	3.27E-08	1.02E-09
Treml4	1	1	1	-8.255290056	2.77E-28	-7.739453145	1.15E-23	-9.162091448	3.88E-32	9.07E-25	3.76E-20	1.27E-28
Wwc2	1	1	1	0.895925059	6.66E-11	1.358009358	1.02E-19	1.480675842	2.69E-22	1.09E-07	2.78E-16	7.34E-19
Ank1	1	0	0	0.964746567	0.000013	0.682702939	0.011212556	0.467694716	0.063106137	0.015197	0.150413682	0.99911736
Gm10693	1	0	0	4.913796737	4.57E-08	2.237979759	0.011369575	2.502626859	0.00459953	6.23E-05	0.151148166	0.34530233
Serpinb2	1	0	0	-2.514443496	0.0000331	-0.547801438	0.086542548	-0.740440944	0.021168514	0.036114307	0.373547955	0.734764146
A530040E14Rik	0	1	1	-0.429181642	0.423851175	-0.735200557	0.00000178	-0.756796887	0.000000819	0.999996572	0.00130928	0.000744653
Ch25h	0	1	1	-0.680318516	0.135752882	-0.823895429	0.000229246	-1.247469642	0.000000027	0.999996572	0.029776508	2.95E-05
Fbrsl1	0	1	1	-0.246420289	0.078860646	-0.817603997	2.19E-09	-0.883698665	1.41E-10	0.999996572	3.58E-06	2.31E-07
Gm15056	0	1	1	0.565249444	0.509500412	-0.895037538	0.00000318	-0.689502918	0.0000312	0.999996572	0.001927551	0.01458912
Hilpda	0	1	1	-0.077431392	0.6161907	-0.552752887	0.0000241	-0.569455927	0.0000119	0.999996572	0.008391928	0.00695555
Ier2	0	1	1	-0.255505285	0.168476729	-0.694786935	0.000166469	-0.726846924	0.000110668	0.999996572	0.024786381	0.033540602
Ildr1	0	1	1	-0.029001357	0.972390315	-0.729143985	0.000461558	-0.832616907	0.0000976	0.999996572	0.039548996	0.030717723
Lipg	0	1	1	-0.247778696	0.740656542	-0.910690072	6.63E-09	-0.977245768	1.63E-09	0.999996572	9.86E-06	2.05E-06
Mt1	0	1	1	0.175584813	0.158166825	-0.63763422	0.000000214	-0.591618536	0.00000166	0.999996572	0.000218895	0.001293693
Mt2	0	1	1	0.348056667	0.113553526	-0.628412974	0.000517286	-0.784593088	0.0000158	0.999996572	0.043285482	0.008619427
Nfil3	0	1	1	-0.026842667	0.847776828	-0.503801857	0.000149607	-0.594182974	0.00000822	0.999996572	0.023186299	0.005381141
Thbs4	0	1	1	0.243109758	0.769492315	-0.886021868	0.000062	-0.920725213	0.0000227	0.999996572	0.014921941	0.011257824
Timp1	0	1	1	0.441499918	0.134219818	-1.146535668	8.47E-08	-1.035620034	0.00000129	0.999996572	0.000106631	0.001055607
2310022B05Rik	0	1	0	-0.114994332	0.434917523	-0.540431566	0.000256796	-0.258546991	0.075667297	0.999996572	0.030676813	0.99911736
Abca5	0	1	0	0.606895368	0.030360815	1.532124383	0.000613751	0.579310313	0.170186805	0.999996572	0.04652187	0.99911736
Adam10	0	1	0	0.052933524	0.703754854	0.534461507	0.000150174	0.114213569	0.418926564	0.999996572	0.023186299	0.99911736
Akt2	0	1	0	-0.074357644	0.6215462	-0.56683235	0.000166978	-0.224187989	0.138445096	0.999996572	0.024786381	0.99911736
Aldh2	0	1	0	-0.114038796	0.414208007	-0.513241707	0.000245789	-0.188727029	0.178158223	0.999996572	0.030129204	0.99911736
Ankrd28	0	1	0	0.090648063	0.661731642	0.901516344	0.0000176	0.497170975	0.017494333	0.999996572	0.006806768	0.686600129
Arf5	0	1	0	-0.13069151	0.304319339	-0.520036706	0.0000403	-0.324675574	0.010440439	0.999996572	0.011685898	0.517984147
Atf7ip2	0	1	0	0.456617023	0.512640941	2.756463147	0.000697459	1.3758752	0.052382491	0.999996572	0.049453665	0.988127267
AW549877	0	1	0	0.074396389	0.711641435	0.698145653	0.000494986	0.0773471	0.698401201	0.999996572	0.041757427	0.99911736
Bet1	0	1	0	0.235066225	0.209958753	0.661527342	0.000708921	0.237768635	0.21148836	0.999996572	0.049453665	0.99911736
C330027C09Rik	0	1	0	0.41495755	0.158611802	1.149726755	0.000403182	0.006962586	0.983818489	0.999996572	0.037922279	0.99911736
Calcrl	0	1	0	0.16956079	0.337886219	0.74596499	0.0000252	0.655895652	0.000669842	0.999996572	0.00859215	0.109193142
Cav2	0	1	0	0.113396231	0.517995488	0.657912886	0.000268488	0.592169051	0.001388273	0.999996572	0.030988849	0.170830646
Cbr2	0	1	0	-0.608814062	0.02241599	-1.023072884	0.000185076	-0.663601883	0.017420911	0.999996572	0.026416005	0.68536209
Ccrl2	0	1	0	-0.047045399	0.771225013	-0.566698814	0.000091	-0.395308441	0.006412226	0.999996572	0.016923932	0.394092854
Cd207	0	1	0	-0.434446503	0.001863782	-0.590779505	0.0000369	-0.194043715	0.264782983	0.648992685	0.011183433	0.99911736
Cd300lf	0	1	0	-0.194539322	0.465637443	0.848129934	0.00041499	0.292578082	0.190552805	0.999996572	0.038589354	0.99911736
Cd59a	0	1	0	-0.65851128	0.000162171	-0.746006899	0.0000833	-0.251258616	0.227534419	0.120640481	0.0162491	0.99911736
Cd80	0	1	0	0.28165925	0.258705038	-0.79593002	0.000445871	-0.608118842	0.008146263	0.999996572	0.039453725	0.455023004
Celf2	0	1	0	0.08524644	0.540625284	0.515425859	0.000269118	0.130867672	0.361514477	0.999996572	0.030988849	0.99911736
Celsr3	0	1	0	-0.159798468	0.402762215	0.777435432	0.000652297	-0.049683154	0.792387146	0.999996572	0.047295217	0.99911736
Cnn3	0	1	0	-0.419776813	0.353913441	-0.679801655	0.0000712	-0.478296163	0.005443792	0.999996572	0.015746746	0.374340756
Cnppd1	0	1	0	-0.057126639	0.721298425	-0.606407913	0.000155582	-0.229268423	0.153596251	0.999996572	0.023576435	0.99911736
Ctdnep1	0	1	0	-0.07641251	0.634715425	-0.64105335	0.000067	-0.320321139	0.04675953	0.999996572	0.015229472	0.962599331
Dennd1b	0	1	0	0.135567074	0.391181075	0.67304606	0.0000171	-0.045317466	0.771014955	0.999996572	0.006806768	0.99911736
Dgka	0	1	0	-0.134186051	0.510610855	-0.686897321	0.000632643	-0.02549101	0.901483676	0.999996572	0.046849934	0.99911736
Dmwd	0	1	0	-0.479608781	0.02837344	-0.673163823	0.0000148	-0.389718986	0.008731996	0.999996572	0.006503332	0.47320479
Dnase1l3	0	1	0	0.267369803	0.678992019	-0.702287441	0.000262483	-0.538047044	0.005114923	0.999996572	0.030905013	0.360822542
Ece1	0	1	0	-0.11887959	0.470191187	-0.770171907	0.00000244	-0.273146105	0.097186149	0.999996572	0.001597322	0.99911736
Edil3	0	1	0	0.716327812	0.002655771	0.901962144	0.0003883	-0.022678887	0.921839516	0.739590358	0.037789094	0.99911736
Ehd1	0	1	0	-0.223172761	0.212175294	-0.616938928	0.000536675	-0.221487056	0.214283211	0.999996572	0.043697627	0.99911736
Eif3f	0	1	0	-0.027680659	0.855088831	-0.540179032	0.00038837	-0.279156652	0.067463149	0.999996572	0.037789094	0.99911736
Ell2	0	1	0	0.094047389	0.548821088	0.536223392	0.000626681	0.082241753	0.598154037	0.999996572	0.046849934	0.99911736
Eva1b	0	1	0	-0.273735335	0.184685038	-0.720146595	0.000213472	-0.48228401	0.014172946	0.999996572	0.028873411	0.609465384
F10	0	1	0	-0.283842371	0.098025384	-0.614963426	0.000291734	-0.259388435	0.126472213	0.999996572	0.032479719	0.99911736
Fabp7	0	1	0	-0.700284503	0.000792223	-0.732461757	0.000546221	-0.462506237	0.032983577	0.381338871	0.044036714	0.852538976
Fam115a	0	1	0	1.077404544	0.002018429	1.542635044	0.000094	1.302249776	0.00226894	0.674155286	0.017285438	0.238035077
Fam134a	0	1	0	-0.074701219	0.657329951	-0.674868283	0.0000645	-0.263293651	0.119406293	0.999996572	0.014936856	0.99911736
Fam135a	0	1	0	0.626871143	0.033627533	1.020563176	0.000582277	0.540076934	0.070056961	0.999996572	0.045815122	0.99911736
Fam53a	0	1	0	-0.205290812	0.248132975	-0.671529408	0.000238179	0.083199297	0.669375081	0.999996572	0.030129204	0.99911736
Fam84b	0	1	0	-0.075681358	0.571465361	0.583044058	0.0000782	0.16390795	0.234776086	0.999996572	0.0162491	0.99911736
Fcgrt	0	1	0	-0.055767196	0.735806467	-0.580702088	0.000590841	-0.161890886	0.341428064	0.999996572	0.046266525	0.99911736
Fcrls	0	1	0	-0.428828799	0.0185533	-0.886488905	0.00000594	-0.440328333	0.039862331	0.999996572	0.003240468	0.914533934
Flrt2	0	1	0	0.110282822	0.498243377	0.780279262	0.00000199	0.107125739	0.503850205	0.999996572	0.001357014	0.99911736
Foxp4	0	1	0	-0.241959948	0.320013988	-0.835397012	0.000450874	-0.408723559	0.08810245	0.999996572	0.039453725	0.99911736
Gadd45gip1	0	1	0	-0.053891258	0.739612358	-0.680037391	0.000088	-0.24154919	0.182197266	0.999996572	0.016860786	0.99911736
Gbp4	0	1	0	-0.035849642	0.903358744	0.566073183	0.0000287	-0.123351263	0.361529782	0.999996572	0.009394084	0.99911736
Glipr2	0	1	0	0.007335164	0.965200685	-0.586063082	0.00000588	-0.409482411	0.001601018	0.999996572	0.003240468	0.190331836

Gm10521	0	1	0	0.007868133	0.98004914	2.116038189	2.24E-08	0.330470997	0.302301417	0.999996572	3.05E-05	0.99911736
Gm10524	0	1	0	0.114156613	0.702042608	1.365481866	0.000243914	1.000695241	0.011786767	0.999996572	0.030129204	0.550152296
Gm13340	0	1	0	-0.297443893	0.214141361	0.883300564	0.000222682	0.283097176	0.236822465	0.999996572	0.029629379	0.99911736
Gm14446	0	1	0	-0.047261447	0.824565972	0.721204531	0.0000684	-0.221939603	0.218763022	0.999996572	0.015334718	0.99911736
Gm15989	0	1	0	-2.171010503	0.01280363	-3.312432402	0.000197724	-3.004307987	0.001830281	0.999996572	0.027657701	0.208881531
Gm20204	0	1	0	-0.140903062	0.367889934	0.751396006	0.000304308	0.131983729	0.485932505	0.999996572	0.033650708	0.99911736
Gm28439	0	1	0	-0.380579535	0.283767456	1.265227035	0.000249866	0.482959907	0.16652294	0.999996572	0.030129204	0.99911736
Gmfb	0	1	0	0.176937938	0.32815718	0.621613544	0.000601776	0.22355114	0.220943294	0.999996572	0.04652187	0.99911736
Got1	0	1	0	0.005186056	0.9721802	-0.547405546	0.000247667	-0.396866561	0.007695351	0.999996572	0.030129204	0.444178354
Gprc5c	0	1	0	-0.360938777	0.082885147	-0.92286943	0.0000407	-0.575919996	0.046429206	0.999996572	0.011685898	0.960632598
Gstm2	0	1	0	-0.273590586	0.059502195	-0.559121536	0.00027467	-0.290544466	0.059996827	0.999996572	0.031217008	0.99911736
H2afj	0	1	0	-0.318369493	0.043736869	-0.644079075	0.000112049	-0.442916565	0.008653802	0.999996572	0.019303094	0.472093745
Hccs	0	1	0	0.22495169	0.294069865	0.799654437	0.000402268	0.245074237	0.282469612	0.999996572	0.037922279	0.99911736
Hmgb2	0	1	0	0.469117533	0.011204252	0.784971814	0.0000475	0.110110688	0.575614471	0.999996572	0.012670452	0.99911736
Hmgcs1	0	1	0	0.058151904	0.661759234	0.557500677	0.0000435	0.183707827	0.182201521	0.999996572	0.011919903	0.99911736
Hmmr	0	1	0	0.606711172	0.006990432	1.281387473	0.000129557	0.025320573	0.938898764	0.999996572	0.021025935	0.99911736
Hnmt	0	1	0	0.32331761	0.104051407	0.790015288	0.000388444	0.514162615	0.025006626	0.999996572	0.037789094	0.775195285
Htra4	0	1	0	-0.471569774	0.025995852	-0.748636961	0.0000538	-0.545550777	0.004197116	0.999996572	0.013546012	0.325170829
Ido1	0	1	0	1.268506662	0.044756466	0.67666963	0.00010102	0.182647509	0.291429037	0.999996572	0.01797058	0.99911736
Ifitm2	0	1	0	-0.203723029	0.245383946	-0.682260996	0.0000958	-0.425471739	0.015006042	0.999996572	0.017420698	0.624444772
Ifitm3	0	1	0	0.017977171	0.906841732	-0.563791045	0.000220316	-0.354586835	0.020134878	0.999996572	0.02955485	0.730164761
Impact	0	1	0	0.124440502	0.539370159	0.698688778	0.000715145	0.348980697	0.082586504	0.999996572	0.049593488	0.99911736
Irf8	0	1	0	-0.086899315	0.528459112	-0.510679498	0.000200014	-0.215901179	0.116443686	0.999996572	0.027740925	0.99911736
Jade2	0	1	0	-0.322926598	0.139285713	-0.775638392	0.000411886	-0.22281536	0.320083956	0.999996572	0.038519579	0.99911736
Junb	0	1	0	-0.304119356	0.047682667	-0.546820437	0.000358352	-0.491275307	0.001353956	0.999996572	0.036493435	0.168362621
Jund	0	1	0	-0.423813974	0.014167459	-0.611780576	0.000393692	-0.308806386	0.074195675	0.999996572	0.03790096	0.99911736
Kdm6b	0	1	0	-0.250751401	0.127494979	-0.59289532	0.000210861	-0.250065083	0.116904642	0.999996572	0.028757926	0.99911736
Kif11	0	1	0	0.550221426	0.031648877	1.180165516	0.000262389	0.047118481	0.91050906	0.999996572	0.030905013	0.99911736
Kitl	0	1	0	0.69438565	0.002446605	0.905631009	0.000014	0.678306509	0.001269269	0.725167985	0.006364556	0.161029895
Ldhb	0	1	0	-0.343899723	0.07002334	-0.794663614	0.000337093	-0.404044424	0.099001715	0.999996572	0.035592671	0.99911736
Lgals9	0	1	0	0.238932705	0.063626185	-0.522574234	0.0000363	-0.202972032	0.108669225	0.999996572	0.011183433	0.99911736
Lipo1	0	1	0	0.204191554	0.257353957	0.739337515	0.000048	0.183873963	0.309147733	0.999996572	0.012670452	0.99911736
Lrrc4	0	1	0	-0.884143934	0.196555851	-0.576086892	0.00000184	-0.348338863	0.00543935	0.999996572	0.00130928	0.374340756
Lyz2	0	1	0	-0.469639982	0.005060654	-0.607842366	0.000285901	-0.406150229	0.015357284	0.999996572	0.032048327	0.631999243
Mag	0	1	0	-0.143169808	0.431244545	-0.670293816	0.000106914	-0.306473627	0.082773471	0.999996572	0.01861441	0.99911736
Man1a2	0	1	0	0.08855863	0.486157272	0.54594094	0.0000283	0.164338537	0.205412275	0.999996572	0.009394084	0.99911736
Map4k3	0	1	0	0.091343683	0.573139901	0.589091344	0.000324098	0.288824361	0.076120122	0.999996572	0.034692818	0.99911736
Mapkbp1	0	1	0	-0.269493822	0.152550215	-0.686082701	0.000242687	-0.369616673	0.048608527	0.999996572	0.030129204	0.969419669
Mmgt1	0	1	0	0.213892666	0.245255587	0.707081116	0.000603844	0.12692892	0.542473172	0.999996572	0.04652187	0.99911736
Mmp25	0	1	0	0.211394743	0.284818917	-0.536678127	0.0000156	-0.450028024	0.000314878	0.999996572	0.0065464	0.066925888
mmu-mir-5103	0	1	0	0.287019938	0.324532038	1.035215576	0.00070654	0.696472932	0.014874839	0.999996572	0.049453665	0.622612826
Mob3a	0	1	0	-0.062349933	0.675362384	-0.542192044	0.000320448	-0.159079395	0.289653131	0.999996572	0.034692818	0.99911736
Mpp6	0	1	0	0.097522624	0.521923961	0.766237777	0.00000103	0.355100444	0.034503981	0.999996572	0.000842849	0.862711002
Mpp7	0	1	0	0.889071188	0.000502954	1.033852703	0.0000639	0.311178932	0.22480551	0.274378172	0.014936856	0.99911736
Mpst	0	1	0	-0.017522491	0.898894427	-0.528293044	0.000390221	-0.297542535	0.043098429	0.999996572	0.037789094	0.939212901
Msmo1	0	1	0	-0.005547516	0.968463426	0.510231783	0.000431928	0.280938211	0.052144235	0.999996572	0.03936649	0.988127267
mt-Co1	0	1	0	-0.214518866	0.187390811	0.585028529	0.000323875	0.186169419	0.252566705	0.999996572	0.034692818	0.99911736
mt-Nd4	0	1	0	-0.174812657	0.376284351	0.692268099	0.000458526	0.170729023	0.387749145	0.999996572	0.039495982	0.99911736
Myo18b	0	1	0	1.359679001	0.006248212	-1.356585735	0.000354867	-0.51836692	0.308962765	0.999996572	0.036493435	0.99911736
Napb	0	1	0	-0.02375196	0.932573208	1.169901786	0.0000886	0.492756462	0.064644265	0.999996572	0.016860786	0.99911736
Ndrg4	0	1	0	0.549158028	0.002904485	1.046755423	0.0000183	0.580003642	0.00752628	0.792246692	0.006806768	0.436791129
Nek2	0	1	0	0.247230017	0.291828157	1.209633235	0.000526287	0.443270097	0.221762684	0.999996572	0.043525212	0.99911736
Nfix	0	1	0	-0.258151183	0.409136608	-1.161548961	0.000539849	-0.769642505	0.025125416	0.999996572	0.043738459	0.775195285
Nfkb2	0	1	0	-0.090599918	0.648230305	-0.711719617	0.000322879	-0.329386943	0.096308981	0.999996572	0.034692818	0.99911736
Nfkbib	0	1	0	-0.089607598	0.572497499	-0.581780984	0.000204936	-0.297553372	0.057971457	0.999996572	0.028184728	0.99911736
Nktr	0	1	0	-0.02018562	0.924871592	0.863139019	0.0000549	0.150679934	0.479846979	0.999996572	0.013613536	0.99911736
Nr4a1	0	1	0	0.005993486	0.97712798	-0.737660512	0.00033218	-0.663481597	0.00566676	0.999996572	0.035301675	0.38009096
Nusap1	0	1	0	0.567437417	0.02562545	1.084035052	0.000104396	0.268379443	0.465210222	0.999996572	0.018371451	0.99911736
Pdcd2	0	1	0	0.088804419	0.707675677	-0.946333742	0.0000797	-0.487244903	0.054651869	0.999996572	0.0162491	0.997061789
Pde1b	0	1	0	-0.284972866	0.212930907	0.809167924	0.000359003	0.25000517	0.270568517	0.999996572	0.036493435	0.99911736
Pde7b	0	1	0	0.136730068	0.445251203	0.555525193	0.0000181	0.028989021	0.820351378	0.999996572	0.006806768	0.99911736
Pdlim4	0	1	0	-0.203928505	0.147835077	-0.637047768	0.00000795	-0.44544981	0.001670273	0.999996572	0.004065928	0.196659625
Pfkl	0	1	0	-0.146773987	0.338346703	-0.59930592	0.0000827	-0.356255149	0.019093464	0.999996572	0.0162491	0.718353177
Pgap1	0	1	0	-0.008679677	0.946214888	0.640473161	0.0000228	0.430888399	0.001880652	0.999996572	0.008147422	0.210198001
Pgp	0	1	0	-0.058210099	0.720084598	-0.653787814	0.00011605	-0.395243293	0.016805663	0.999996572	0.019777383	0.669200683
Pim3	0	1	0	-0.165609321	0.445212545	-0.756333863	0.000451257	-0.471644889	0.027752377	0.999996572	0.039453725	0.806741389
Pink1	0	1	0	-0.240336188	0.084675139	-0.600992719	0.000038	-0.214589686	0.143694639	0.999996572	0.011307418	0.99911736
Pla1a	0	1	0	-0.66422637	0.454998535	-1.095365993	0.000439335	-1.005761522	0.001106038	0.999996572	0.03936649	0.145473708
Pls3	0	1	0	0.522488414	0.002518985	0.665857398	0.00012591	0.578622367	0.000933382	0.725167985	0.020814576	0.13399763
Pnpt1	0	1	0	0.276242004	0.099473457	0.558704082	0.000526579	-0.016941536	0.916431775	0.999996572	0.043525212	0.99911736
Ppp1r15a	0	1	0	-0.049145821	0.74214791	-0.54088525	0.000226395	-0.214197506	0.145096364	0.999996572	0.029641445	0.99911736
Prex1	0	1	0	-0.125592401	0.312398358	-0.503997361	0.0000496	-0.145819986	0.240465967	0.999996572	0.012884978	0.99911736
Prkdc	0	1	0	0.11382626	0.556199103	0.756796105	0.000639265	0.111147942	0.617106769	0.999996572	0.046919707	0.99911736
Ptms	0	1	0	-0.205790952	0.196370095	-0.655142937	0.0000437	-0.10109068	0.528020858	0.999996572	0.011919903	0.99911736
Ptplb	0	1	0	0.022491766	0.890227258	-0.624327652	0.0000759	-0.261827807	0.117105335	0.999996572	0.0162491	0.99911736
Pvrl2	0	1	0	-0.183215936	0.295107184	-0.886198355	0.000000239	-0.358276901	0.038446881	0.999996572	0.000230087	0.901047149
Raver1	0	1	0	0.038508098	0.757805571	-0.520568154	0.0000343	-0.225105968	0.073718263	0.999996572	0.010858212	0.99911736
Rcl1	0	1	0	-0.117031014	0.574305267	-0.843247674	0.0000345	-0.61175057	0.003827266	0.999996572	0.010858212	0.307044291
Relb	0	1	0	-0.237172432	0.120324038	-0.516252485	0.00065006	-0.164893985	0.278228948	0.999996572	0.047295217	0.99911736

Rilpl2	0	1	0	-0.012608398	0.936136581	-0.645503797	0.0000424	-0.108455824	0.492619418	0.999996572	0.011919903	0.99911736
Rnd1	0	1	0	0.155831731	0.551923466	-0.840655865	0.000440185	-0.527739783	0.025767876	0.999996572	0.03936649	0.783860704
Rnf128	0	1	0	0.021155322	0.870153541	0.562356394	0.0000178	0.307418539	0.018491082	0.999996572	0.006806768	0.703779181
Rnf44	0	1	0	-0.163815125	0.17293742	-0.562846437	0.0000033	-0.318464976	0.008496887	0.999996572	0.00192885	0.468215665
RP23-13A13.6	0	1	0	-0.3636166	0.377152191	1.556036101	0.000268912	0.407384649	0.346458585	0.999996572	0.030988849	0.99911736
RP23-456B9.18	0	1	0	-0.106806492	0.826355007	1.879284247	0.000631433	-0.330843269	0.520956139	0.999996572	0.046849934	0.99911736
RP24-443M8.2	0	1	0	-0.122937968	0.585844153	0.844368687	0.000555307	0.433604981	0.0578146	0.999996572	0.04433246	0.99911736
Rps6ka4	0	1	0	-0.089945322	0.552096011	-0.576757856	0.000129758	-0.351218458	0.019815819	0.999996572	0.021025935	0.72937282
Runx3	0	1	0	-0.115684021	0.385137528	-0.545680292	0.0000106	-0.42257201	0.000630803	0.999996572	0.005150476	0.10753877
Scand1	0	1	0	-0.379412048	0.060655299	-0.801775514	0.0000834	-0.538551787	0.008912269	0.999996572	0.0162491	0.478441068
Sdc4	0	1	0	-0.143245112	0.244370548	-0.526864537	0.0000133	-0.404998915	0.000842188	0.999996572	0.00621908	0.127622674
Serpinb6b	0	1	0	0.379698261	0.007668672	0.658077996	0.0000031	0.254755085	0.069789121	0.999996572	0.001927551	0.99911736
Sgk3	0	1	0	0.078082759	0.60028859	0.573699841	0.000124331	0.047329907	0.751762828	0.999996572	0.020763277	0.99911736
Sh3bp4	0	1	0	-0.170272677	0.266714479	-0.509609094	0.000453214	-0.151134993	0.306158765	0.999996572	0.039453725	0.99911736
Slc15a2	0	1	0	1.288090868	0.184137279	3.676997507	0.000145048	2.959949449	0.002312063	0.999996572	0.022825534	0.24099194
Slc16a3	0	1	0	-0.046769078	0.738474009	-0.547784612	0.0000812	-0.320031955	0.021173721	0.999996572	0.0162491	0.734764146
Slc40a1	0	1	0	0.174191942	0.297770103	0.62083362	0.000244654	0.482919325	0.003991781	0.999996572	0.030129204	0.315601391
Slc4a7	0	1	0	0.057345983	0.67336872	0.721896696	0.000000622	0.351541265	0.034287994	0.999996572	0.000535771	0.860670721
Smagp	0	1	0	-0.479576175	0.022401389	-0.91540408	0.0000151	-0.665291987	0.003337924	0.999996572	0.006503332	0.28604829
Spp1	0	1	0	0.587068352	0.000301471	0.639317546	0.0000829	0.531154759	0.001077885	0.180992179	0.0162491	0.143420048
Sptssa	0	1	0	0.069057414	0.671182004	0.698385895	0.0000796	0.378446955	0.030785464	0.999996572	0.0162491	0.825958859
Ssbp4	0	1	0	-0.225630665	0.137043221	-0.742592718	0.00000119	-0.41792707	0.006476525	0.999996572	0.000927407	0.394092854
St18	0	1	0	0.144935757	0.470034737	0.781866303	0.00016811	0.314354497	0.136833817	0.999996572	0.024786381	0.99911736
Stard9	0	1	0	-0.1776379	0.356873921	0.781142957	0.000232823	0.681636126	0.000796662	0.999996572	0.030003002	0.123001607
Stat5a	0	1	0	-0.073826561	0.688065855	-0.628631631	0.000609735	-0.256553457	0.171537238	0.999996572	0.04652187	0.99911736
Synpo	0	1	0	0.36036839	0.111282506	-0.604821402	0.000446873	-0.537612423	0.005066669	0.999996572	0.039453725	0.359557336
Tbc1d1	0	1	0	-0.123039404	0.39005071	-0.57131206	0.0000648	-0.370833993	0.010130769	0.999996572	0.014936856	0.508670491
Tgfbr1	0	1	0	0.110047227	0.524488614	0.844160476	0.00000741	0.425223405	0.019585291	0.999996572	0.003912002	0.725187494
Thbs1	0	1	0	-0.361674229	0.065839644	-0.737389658	0.0000994	-0.70022536	0.000216944	0.999996572	0.017876708	0.05109021
Tlr13	0	1	0	-0.038903219	0.821883781	0.736689037	0.0000229	0.179366022	0.299958014	0.999996572	0.008147422	0.99911736
Tlr4	0	1	0	0.089375186	0.639014137	0.842612068	0.0000107	0.400671942	0.036771603	0.999996572	0.005150476	0.882410637
Tmem110	0	1	0	-0.221173941	0.344249807	-0.838940462	0.000284861	-0.483160123	0.039954281	0.999996572	0.032048327	0.914533934
Tpi1	0	1	0	0.130678146	0.321150098	-0.502010309	0.000135945	-0.286283698	0.029475798	0.999996572	0.021600737	0.823501586
Ttc7	0	1	0	-0.088764149	0.525841186	-0.570476199	0.0000523	-0.217084298	0.125154966	0.999996572	0.013374091	0.99911736
Xkr8	0	1	0	-0.19530555	0.277596629	-0.58178155	0.000400775	-0.383762123	0.020464487	0.999996572	0.037922279	0.730164761
Zc3h7a	0	1	0	0.028828508	0.880364095	0.645093482	0.000655995	0.061773565	0.744590492	0.999996572	0.047295217	0.99911736
Zdhhc2	0	1	0	0.999951763	0.002236492	0.883078708	0.000155296	0.566327344	0.016199846	0.725167985	0.023576435	0.654633777
Zfand2b	0	1	0	-0.009262204	0.952600275	-0.557734703	0.00038287	-0.311267431	0.048516798	0.999996572	0.037789094	0.969419669
Zfp445	0	1	0	0.004136011	0.985871265	0.835558008	0.000458024	0.048333413	0.838404747	0.999996572	0.039495982	0.99911736
Zfp451	0	1	0	-0.026016012	0.876094672	0.676665174	0.000117219	0.113249891	0.498327201	0.999996572	0.019777383	0.99911736
Zfp597	0	1	0	0.108205577	0.608073861	0.809722823	0.000173325	0.101610718	0.628531759	0.999996572	0.025327116	0.99911736
Zmym6	0	1	0	0.104591869	0.650514712	0.926433964	0.000225771	0.392390095	0.09520649	0.999996572	0.029641445	0.99911736
Adra1a	0	0	1	0.123525906	0.526677174	0.403848713	0.061095215	0.901168803	0.0000101	0.999996572	0.320895593	0.006122096
Agap1	0	0	1	-0.209099534	0.146711564	0.108944496	0.531545116	0.724742949	0.000180147	0.999996572	0.823794258	0.046798187
Angptl2	0	0	1	0.055835207	0.766708115	0.523471517	0.006373483	0.817868165	0.0000183	0.999996572	0.122715792	0.009661219
Apbb2	0	0	1	-0.083980288	0.610498176	0.511608597	0.003424317	0.677059768	0.0000813	0.999996572	0.096832721	0.0271542
Arhgef18	0	0	1	0.334390742	0.144634579	0.831805815	0.00088348	0.907175296	0.000198617	0.999996572	0.054977314	0.049900678
Bcl2l14	0	0	1	0.337061247	0.632960514	-0.621484471	0.00124865	-0.768497378	0.000139412	0.999996572	0.064250761	0.038671471
Bsn	0	0	1	0.147232629	0.313325231	0.333665931	0.204272902	0.962458675	0.000015	0.999996572	0.55085357	0.008465172
C77080	0	0	1	0.162200722	0.339738632	0.594595893	0.001861916	0.845373344	0.00000191	0.999996572	0.07356096	0.001420866
Cacna1a	0	0	1	-0.05676569	0.735603749	0.17136476	0.410752299	0.806794415	0.0000458	0.999996572	0.744378515	0.019555272
Cacna1d	0	0	1	-0.384525522	0.003449522	-0.425754796	0.001066367	-0.513621559	0.000064	0.910562533	0.06017987	0.024358698
Ccl4	0	0	1	-0.276104986	0.036874892	-0.20776915	0.072584296	-0.620380224	8.38E-08	0.999996572	0.34673514	8.57E-05
Cd83	0	0	1	-0.229357064	0.188894043	-0.528830917	0.00135769	-0.65598756	0.0000785	0.999996572	0.064727007	0.0271542
Dio2	0	0	1	0.415640309	0.201692604	1.078086383	0.007550451	1.402340997	0.0000328	0.999996572	0.131039959	0.014911244
Dock10	0	0	1	-0.054888755	0.681644129	0.480562505	0.000356145	0.508762076	0.000201237	0.999996572	0.036493435	0.049900678
Dusp2	0	0	1	-0.214623479	0.420254035	-0.622254567	0.006309316	-0.965773829	0.0000812	0.999996572	0.121910585	0.0271542
F3	0	0	1	0.215298593	0.557364915	-0.411010191	0.033922173	-0.784760448	0.000102861	0.999996572	0.246033848	0.0317627
Gdpd1	0	0	1	0.018084075	0.904438855	0.194211493	0.217155829	0.657382921	0.0000466	0.999996572	0.566550661	0.019555272
H2-Q7	0	0	1	0.006315004	0.983849284	-0.826730911	0.00324051	-1.128728493	0.00005	0.999996572	0.094199266	0.0204575
Kcnj2	0	0	1	0.07249103	0.660471855	0.361779584	0.109793774	0.862658488	0.0000055	0.999996572	0.413391266	0.003750542
Maoa	0	0	1	0.43382499	0.020572031	0.382862893	0.054162085	0.832119409	0.000122846	0.999996572	0.305766362	0.035901744
Nav2	0	0	1	-0.020932154	0.91574623	-0.216172573	0.32004069	0.842980393	0.000068	0.999996572	0.672373034	0.025292909
Pcdh7	0	0	1	-0.003932802	0.980440674	0.420397323	0.009980025	0.643811586	0.000113661	0.999996572	0.144413238	0.03382138
Pecam1	0	0	1	0.588030454	0.044299894	-0.397248723	0.025118823	-0.685832554	0.0000831	0.999996572	0.216287632	0.027200292
Plxna2	0	0	1	0.030243016	0.848166501	0.459628351	0.127678319	1.509741743	7.86E-09	0.999996572	0.444724979	9.19E-06
Plxnd1	0	0	1	-0.140595651	0.281913348	-0.083038942	0.551847708	0.538298972	0.0000797	0.999996572	0.836031575	0.0271542
Rgcc	0	0	1	-0.213897626	0.410972307	-0.326014234	0.017543279	-0.574403152	0.0000624	0.999996572	0.18523439	0.0243152
Rhoh	0	0	1	0.28249173	0.166381542	-0.536132315	0.001869813	-0.642252473	0.000193649	0.999996572	0.07356096	0.04951968
Sectm1a	0	0	1	-0.395969155	0.402865906	-0.139956202	0.419570768	-0.66030394	0.000143043	0.999996572	0.750913278	0.038958861
Selenbp1	0	0	1	0.010342819	0.948914768	-0.467085248	0.012845721	-0.663223002	0.0000367	0.999996572	0.158666468	0.016233303
Serpinb9b	0	0	1	0.428147082	0.018637342	0.543837875	0.002573589	0.691214869	0.00013448	0.999996572	0.084851914	0.037946546
Slc4a11	0	0	1	0.131475288	0.759648837	-0.397267075	0.022024803	-0.868678697	0.000000468	0.999996572	0.205272167	0.000450546
Slpi	0	0	1	-0.312189837	0.018729801	-0.391877749	0.000518389	-0.694803689	4.62E-10	0.999996572	0.043285482	6.87E-07
Steap4	0	0	1	-0.368553349	0.681287203	-0.141756603	0.388297209	-0.930332474	0.00000481	0.999996572	0.725810877	0.003422629
Tmem8b	0	0	1	-0.47415155	0.027674195	-0.907375331	0.001709433	-0.971038122	0.000128959	0.999996572	0.071439651	0.03702707
Trem1	0	0	1	-0.049969129	0.878903703	-0.76112388	0.006949999	-1.092469844	0.0000522	0.999996572	0.12615616	0.020836712
Vegfa	0	0	1	-0.551337226	0.002340865	-0.575901176	0.001093032	-0.655147221	0.000145209	0.725167985	0.060373954	0.038958861

Treml4 promotes inflammatory programs in human and murine macrophages and alters atherosclerosis lesion composition in the Apolipoprotein E deficient mouse

Supplemental table 4- Metascape results- M1 8hr DEG mouse

GroupID	Category	Term	Description	LogP	Log(q-value)	InTerm_InList	Symbols
**1_Summary**	**KEGG Pathway**	**mmu04380**	**Osteoclast differentiation**	**-5.26389**	**-1.10107**	**8/130**	**Akt2,Junb,Jund,Nfkb2,Pira2,Relb,Tgfbr1,Gm14548,Nr4a1,Rps6ka4,Map4k3,Impact,Pde1b,Prkdc,Synpo,Ndrg4**
1_Member	KEGG Pathway	mmu04380	Osteoclast differentiation	-5.26389	-1.10107	8/130	Akt2,Junb,Jund,Nfkb2,Pira2,Relb,Tgfbr1,Gm14548
1_Member	KEGG Pathway	mmu04010	MAPK signaling pathway	-3.24579	-0.40333	8/252	Akt2,Nr4a1,Jund,Nfkb2,Relb,Tgfbr1,Rps6ka4,Map4k3
1_Member	GO Biological Processes	GO:0009314	response to radiation	-2.62053	-0.22727	9/389	Akt2,Impact,Junb,Jund,Pde1b,Prkdc,Synpo,Map4k3,Ndrg4
**2_Summary**	**GO Biological Processes**	**GO:0010623**	**programmed cell death involved in cell development**	**-5.00158**	**-1.10107**	**4/19**	**Dnase1l3,Kitl,Prkdc,Slc4a7**
2_Member	GO Biological Processes	GO:0010623	programmed cell death involved in cell development	-5.00158	-1.10107	4/19	Dnase1l3,Kitl,Prkdc,Slc4a7
3_Summary	GO Biological Processes	GO:0031347	regulation of defense response	-4.72691	-1.10107	15/563	Cd59a,Dnase1l3,Ido1,Lgals9,Gbp4,Prkdc,Nectin2,Stat5a,Tlr4,Mapkbp1,Calcrl,Hmgb2,Treml4,Cd300lf,Tlr13,Adam10,Relb,Thbs1,Dennd1b
3_Member	GO Biological Processes	GO:0031347	regulation of defense response	-4.72691	-1.10107	15/563	Cd59a,Dnase1l3,Ido1,Lgals9,Gbp4,Prkdc,Nectin2,Stat5a,Tlr4,Mapkbp1,Calcrl,Hmgb2,Treml4,Cd300lf,Tlr13
3_Member	GO Biological Processes	GO:0045089	positive regulation of innate immune response	-4.64705	-1.10107	9/206	Lgals9,Prkdc,Nectin2,Stat5a,Tlr4,Hmgb2,Treml4,Cd300lf,Tlr13
3_Member	GO Biological Processes	GO:0002274	myeloid leukocyte activation	-4.04491	-0.87915	8/192	Adam10,Dnase1l3,Lgals9,Nectin2,Relb,Thbs1,Tlr4,Cd300lf
3_Member	GO Biological Processes	GO:0045088	regulation of innate immune response	-4.00926	-0.87826	9/249	Lgals9,Prkdc,Nectin2,Stat5a,Tlr4,Hmgb2,Treml4,Cd300lf,Tlr13
3_Member	GO Biological Processes	GO:0031349	positive regulation of defense response	-3.79679	-0.69797	10/327	Ido1,Lgals9,Prkdc,Nectin2,Stat5a,Tlr4,Hmgb2,Treml4,Cd300lf,Tlr13
3_Member	GO Biological Processes	GO:0002755	MyD88-dependent toll-like receptor signaling pathway	-3.39381	-0.40414	3/20	Tlr4,Cd300lf,Tlr13
3_Member	GO Biological Processes	GO:0002224	toll-like receptor signaling pathway	-3.20743	-0.40333	5/93	Lgals9,Tlr4,Treml4,Cd300lf,Tlr13
3_Member	GO Biological Processes	GO:0002218	activation of innate immune response	-3.2074	-0.40333	6/141	Lgals9,Prkdc,Tlr4,Treml4,Cd300lf,Tlr13
3_Member	GO Biological Processes	GO:0002221	pattern recognition receptor signaling pathway	-2.6651	-0.22727	5/123	Lgals9,Tlr4,Treml4,Cd300lf,Tlr13
3_Member	GO Biological Processes	GO:0002758	innate immune response-activating signal transduction	-2.63458	-0.22727	5/125	Lgals9,Tlr4,Treml4,Cd300lf,Tlr13
3_Member	GO Biological Processes	GO:0002697	regulation of immune effector process	-2.53999	-0.20985	9/400	Cd59a,Dnase1l3,Lgals9,Gbp4,Prkdc,Nectin2,Stat5a,Tlr4,Treml4
3_Member	GO Biological Processes	GO:0050778	positive regulation of immune response	-2.19784	-0.10699	12/710	Cd59a,Ido1,Lgals9,Prkdc,Nectin2,Stat5a,Tlr4,Hmgb2,Treml4,Cd300lf,Tlr13,Dennd1b
**4_Summary**	**GO Biological Processes**	**GO:0006954**	**inflammatory response**	**-4.66207**	**-1.10107**	**16/641**	**Cd59a,Dnase1l3,Ido1,Lgals9,Relb,Stat5a,Thbs1,Timp1,Tlr4,Ccrl2,Calcrl,Rps6ka4,Hmgb2,Kdm6b,Mmp25,Tlr13,Gbp4,Mapkbp1,Rnf128,Runx3,Irf8,Hilpda,Dennd1b,Adam10,Akt2,Fabp7,Thbs4,Treml4,Cd80,Junb,Jund,Nfkbib,Got1**
4_Member	GO Biological Processes	GO:0006954	inflammatory response	-4.66207	-1.10107	16/641	Cd59a,Dnase1l3,Ido1,Lgals9,Relb,Stat5a,Thbs1,Timp1,Tlr4,Ccrl2,Calcrl,Rps6ka4,Hmgb2,Kdm6b,Mmp25,Tlr13
4_Member	GO Biological Processes	GO:0001818	negative regulation of cytokine production	-4.21557	-0.94524	9/234	Cd59a,Ido1,Lgals9,Gbp4,Relb,Thbs1,Tlr4,Mapkbp1,Rnf128
4_Member	GO Biological Processes	GO:0001816	cytokine production	-3.61065	-0.56331	15/709	Runx3,Cd59a,Irf8,Ido1,Lgals9,Gbp4,Relb,Stat5a,Thbs1,Tlr4,Mapkbp1,Rnf128,Hilpda,Hmgb2,Dennd1b
4_Member	GO Biological Processes	GO:0032101	regulation of response to external stimulus	-3.60414	-0.56331	15/710	Adam10,Akt2,Fabp7,Cd59a,Dnase1l3,Ido1,Lgals9,Gbp4,Stat5a,Thbs1,Thbs4,Tlr4,Mapkbp1,Calcrl,Treml4
4_Member	GO Biological Processes	GO:0032655	regulation of interleukin-12 production	-3.27167	-0.40333	4/51	Irf8,Ido1,Thbs1,Tlr4

4_Member	GO Biological Processes	GO:0032615	interleukin-12 production	-3.14651	-0.40333	4/55	Irf8,Ido1,Thbs1,Tlr4
4_Member	GO Biological Processes	GO:0001817	regulation of cytokine production	-3.0585	-0.38462	13/636	Cd59a,Irf8,Ido1,Lgals9,Gbp4,Relb,Stat5a,Thbs1,Tlr4,Mapkbp1,Rnf128,Hilpda,Hmgb2
4_Member	GO Biological Processes	GO:0043032	positive regulation of macrophage activation	-3.04979	-0.38462	3/26	Lgals9,Thbs1,Tlr4
4_Member	GO Biological Processes	GO:0032496	response to lipopolysaccharide	-2.96766	-0.32478	9/346	Cd80,Irf8,Ido1,Junb,Jund,Lgals9,Nfkbib,Tlr4,Hmgb2
4_Member	GO Biological Processes	GO:0032103	positive regulation of response to external stimulus	-2.90087	-0.29938	8/285	Adam10,Akt2,Ido1,Lgals9,Stat5a,Thbs1,Thbs4,Tlr4
4_Member	GO Biological Processes	GO:0002237	response to molecule of bacterial origin	-2.79941	-0.27006	9/366	Cd80,Irf8,Ido1,Junb,Jund,Lgals9,Nfkbib,Tlr4,Hmgb2
4_Member	GO Biological Processes	GO:0032735	positive regulation of interleukin-12 production	-2.70645	-0.23966	3/34	Irf8,Ido1,Tlr4
4_Member	GO Biological Processes	GO:0043030	regulation of macrophage activation	-2.41305	-0.17432	3/43	Lgals9,Thbs1,Tlr4
4_Member	GO Biological Processes	GO:0032653	regulation of interleukin-10 production	-2.27807	-0.11629	3/48	Ido1,Lgals9,Tlr4
4_Member	KEGG Pathway	mmu05144	Malaria	-2.25295	-0.112	3/49	Thbs1,Thbs4,Tlr4
4_Member	GO Biological Processes	GO:0042035	regulation of cytokine biosynthetic process	-2.1966	-0.10699	4/100	Stat5a,Thbs1,Tlr4,Rnf128
4_Member	GO Biological Processes	GO:0032613	interleukin-10 production	-2.1809	-0.1003	3/52	Ido1,Lgals9,Tlr4
4_Member	GO Biological Processes	GO:0050727	regulation of inflammatory response	-2.08305	-0.04773	7/311	Cd59a,Dnase1l3,Ido1,Lgals9,Stat5a,Tlr4,Calcrl
4_Member	GO Biological Processes	GO:0009612	response to mechanical stimulus	-2.04061	-0.02112	5/173	Got1,Junb,Jund,Thbs1,Tlr4
4_Member	GO Biological Processes	GO:0042089	cytokine biosynthetic process	-2.02619	-0.01929	4/112	Stat5a,Thbs1,Tlr4,Rnf128
**5_Summary**	**GO Biological Processes**	**GO:0001936**	**regulation of endothelial cell proliferation**	**-4.38467**	**-0.94524**	**7/126**	**Cav2,Nr4a1,Stat5a,Tgfbr1,Thbs1,Thbs4,Hmgb2,Camk2b,Runx3,Pink1,St18,Fabp7,Ido1,Pdcd2,Prkdc,Tlr4,Kitl,Sgk3,Sdc4,Timp1,Glipr2**
5_Member	GO Biological Processes	GO:0001936	regulation of endothelial cell proliferation	-4.38467	-0.94524	7/126	Cav2,Nr4a1,Stat5a,Tgfbr1,Thbs1,Thbs4,Hmgb2
5_Member	GO Biological Processes	GO:0001935	endothelial cell proliferation	-4.09419	-0.89064	7/140	Cav2,Nr4a1,Stat5a,Tgfbr1,Thbs1,Thbs4,Hmgb2
5_Member	GO Biological Processes	GO:0001938	positive regulation of endothelial cell proliferation	-3.34023	-0.40333	5/87	Cav2,Nr4a1,Stat5a,Thbs4,Hmgb2
5_Member	GO Biological Processes	GO:2001235	positive regulation of apoptotic signaling pathway	-2.69907	-0.23945	6/177	Camk2b,Runx3,Tgfbr1,Thbs1,Pink1,St18
5_Member	GO Biological Processes	GO:0050673	epithelial cell proliferation	-2.46245	-0.19523	9/411	Fabp7,Cav2,Runx3,Nr4a1,Stat5a,Tgfbr1,Thbs1,Thbs4,Hmgb2
5_Member	GO Biological Processes	GO:0050678	regulation of epithelial cell proliferation	-2.37461	-0.15934	8/347	Cav2,Runx3,Nr4a1,Stat5a,Tgfbr1,Thbs1,Thbs4,Hmgb2
5_Member	GO Biological Processes	GO:0043065	positive regulation of apoptotic process	-2.29253	-0.11629	11/603	Camk2b,Runx3,Nr4a1,Ido1,Pdcd2,Prkdc,Tgfbr1,Thbs1,Tlr4,Pink1,St18
5_Member	GO Biological Processes	GO:0043068	positive regulation of programmed cell death	-2.26112	-0.112	11/609	Camk2b,Runx3,Nr4a1,Ido1,Pdcd2,Prkdc,Tgfbr1,Thbs1,Tlr4,Pink1,St18
5_Member	GO Biological Processes	GO:0097191	extrinsic apoptotic signaling pathway	-2.16308	-0.09156	6/228	Runx3,Kitl,Tgfbr1,Thbs1,Hmgb2,Sgk3
5_Member	GO Biological Processes	GO:2001236	regulation of extrinsic apoptotic signaling pathway	-2.15723	-0.09156	5/162	Runx3,Tgfbr1,Thbs1,Hmgb2,Sgk3
5_Member	GO Biological Processes	GO:0001667	ameboidal-type cell migration	-2.07269	-0.04259	8/391	Nr4a1,Kitl,Stat5a,Sdc4,Tgfbr1,Thbs1,Timp1,Glipr2
5_Member	GO Biological Processes	GO:2001233	regulation of apoptotic signaling pathway	-2.01066	-0.01499	8/401	Camk2b,Runx3,Tgfbr1,Thbs1,Pink1,Hmgb2,Sgk3,St18
**6_Summary**	**GO Biological Processes**	**GO:0060326**	**cell chemotaxis**	**-4.27562**	**-0.94524**	**10/286**	**Adam10,Ch25h,Nr4a1,Lgals9,Spp1,Thbs1,Thbs4,Ccrl2,Hmgb2,Prex1,Akt2,Runx3,Celsr3,Flrt2,Hmmr,Sdc4,Stat5a,Edil3,Cav2,Kitl,Tgfbr1,Glipr2**
6_Member	GO Biological Processes	GO:0060326	cell chemotaxis	-4.27562	-0.94524	10/286	Adam10,Ch25h,Nr4a1,Lgals9,Spp1,Thbs1,Thbs4,Ccrl2,Hmgb2,Prex1
6_Member	GO Biological Processes	GO:0006935	chemotaxis	-4.20689	-0.94524	14/553	Adam10,Akt2,Runx3,Ch25h,Nr4a1,Lgals9,Spp1,Thbs1,Thbs4,Ccrl2,Hmgb2,Celsr3,Prex1,Flrt2

6_Member	GO Biological Processes	GO:0042330	taxis	-4.19019	-0.94524	14/555	Adam10,Akt2,Runx3,Ch25h,Nr4a1,Lgals9,Spp1,Thbs1,Thbs4,Ccrl2,Hmgb2,Celsr3,Prex1,Flrt2
6_Member	KEGG Pathway	mmu04512	ECM-receptor interaction	-3.43469	-0.4202	5/83	Hmmr,Spp1,Sdc4,Thbs1,Thbs4
6_Member	GO Biological Processes	GO:0030595	leukocyte chemotaxis	-3.12379	-0.40333	7/202	Adam10,Ch25h,Lgals9,Spp1,Thbs1,Thbs4,Prex1
6_Member	Reactome Gene Sets	R-MMU-18679	Signaling by PDGF	-3.05911	-0.38462	4/58	Spp1,Stat5a,Thbs1,Thbs4
6_Member	GO Biological Processes	GO:0010811	positive regulation of cell-substrate adhesion	-2.72793	-0.25383	5/119	Edil3,Spp1,Sdc4,Thbs1,Prex1
6_Member	GO Biological Processes	GO:0040017	positive regulation of locomotion	-2.61009	-0.22727	11/547	Adam10,Akt2,Cav2,Lgals9,Kitl,Stat5a,Tgfbr1,Thbs1,Thbs4,Prex1,Glipr2
6_Member	GO Biological Processes	GO:0050900	leukocyte migration	-2.59546	-0.22727	8/319	Adam10,Ch25h,Lgals9,Kitl,Spp1,Thbs1,Thbs4,Prex1
6_Member	GO Biological Processes	GO:0002687	positive regulation of leukocyte migration	-2.56075	-0.20985	5/130	Adam10,Lgals9,Kitl,Thbs1,Thbs4
6_Member	GO Biological Processes	GO:0030335	positive regulation of cell migration	-2.41924	-0.17432	10/497	Adam10,Akt2,Lgals9,Kitl,Stat5a,Tgfbr1,Thbs1,Thbs4,Prex1,Glipr2
6_Member	GO Biological Processes	GO:2000147	positive regulation of cell motility	-2.31196	-0.12772	10/515	Adam10,Akt2,Lgals9,Kitl,Stat5a,Tgfbr1,Thbs1,Thbs4,Prex1,Glipr2
6_Member	GO Biological Processes	GO:0048247	lymphocyte chemotaxis	-2.25295	-0.112	3/49	Adam10,Ch25h,Lgals9
6_Member	GO Biological Processes	GO:0051272	positive regulation of cellular component movement	-2.22111	-0.10995	10/531	Adam10,Akt2,Lgals9,Kitl,Stat5a,Tgfbr1,Thbs1,Thbs4,Prex1,Glipr2
6_Member	GO Biological Processes	GO:0002688	regulation of leukocyte chemotaxis	-2.15181	-0.09156	4/103	Adam10,Lgals9,Thbs1,Thbs4
6_Member	GO Biological Processes	GO:0002685	regulation of leukocyte migration	-2.00048	-0.00839	5/177	Adam10,Lgals9,Kitl,Thbs1,Thbs4
7_Summary	GO Biological Processes	GO:0002683	negative regulation of immune system process	-3.34443	-0.40333	11/443	Runx3,Cd59a,Cd80,Ido1,Lgals9,Kitl,Prkdc,Stat5a,Sdc4,Thbs1,Cd300lf,Adam10,Rnd1,Tlr4,Edil3,Spp1,Prex1,Cav2,Tgfbr1,Ifitm3,Sh3bp4,Ndrg4,St18,Mag,Nectin2,Lrrc4,Relb
7_Member	GO Biological Processes	GO:0002683	negative regulation of immune system process	-3.34443	-0.40333	11/443	Runx3,Cd59a,Cd80,Ido1,Lgals9,Kitl,Prkdc,Stat5a,Sdc4,Thbs1,Cd300lf
7_Member	GO Biological Processes	GO:0007162	negative regulation of cell adhesion	-3.06139	-0.38462	8/269	Adam10,Runx3,Cd80,Ido1,Lgals9,Sdc4,Thbs1,Rnd1
7_Member	GO Biological Processes	GO:0050868	negative regulation of T cell activation	-2.7767	-0.27006	5/116	Runx3,Cd80,Ido1,Lgals9,Sdc4
7_Member	GO Biological Processes	GO:0002695	negative regulation of leukocyte activation	-2.77464	-0.27006	6/171	Runx3,Cd80,Ido1,Lgals9,Sdc4,Cd300lf
7_Member	GO Biological Processes	GO:0070663	regulation of leukocyte proliferation	-2.76563	-0.26888	7/233	Cd80,Ido1,Lgals9,Kitl,Stat5a,Sdc4,Tlr4
7_Member	GO Biological Processes	GO:0042130	negative regulation of T cell proliferation	-2.75404	-0.26497	4/70	Cd80,Ido1,Lgals9,Sdc4
7_Member	GO Biological Processes	GO:1903038	negative regulation of leukocyte cell-cell adhesion	-2.64976	-0.22727	5/124	Runx3,Cd80,Ido1,Lgals9,Sdc4
7_Member	GO Biological Processes	GO:0045785	positive regulation of cell adhesion	-2.61308	-0.22727	9/390	Runx3,Cd80,Edil3,Lgals9,Spp1,Stat5a,Sdc4,Thbs1,Prex1
7_Member	GO Biological Processes	GO:0050866	negative regulation of cell activation	-2.53432	-0.20985	6/191	Runx3,Cd80,Ido1,Lgals9,Sdc4,Cd300lf
7_Member	GO Biological Processes	GO:0030155	regulation of cell adhesion	-2.53052	-0.20985	12/644	Adam10,Runx3,Cd80,Edil3,Ido1,Lgals9,Spp1,Stat5a,Sdc4,Thbs1,Rnd1,Prex1
7_Member	GO Biological Processes	GO:0032945	negative regulation of mononuclear cell proliferation	-2.48409	-0.19819	4/83	Cd80,Ido1,Lgals9,Sdc4
7_Member	GO Biological Processes	GO:0050672	negative regulation of lymphocyte proliferation	-2.48409	-0.19819	4/83	Cd80,Ido1,Lgals9,Sdc4
7_Member	GO Biological Processes	GO:0070664	negative regulation of leukocyte proliferation	-2.41066	-0.17432	4/87	Cd80,Ido1,Lgals9,Sdc4
7_Member	GO Biological Processes	GO:0008285	negative regulation of cell proliferation	-2.39857	-0.16647	12/669	Cav2,Runx3,Cd80,Ido1,Lgals9,Sdc4,Tgfbr1,Thbs1,Ifitm3,Sh3bp4,Ndrg4,St18
7_Member	GO Biological Processes	GO:0051250	negative regulation of lymphocyte activation	-2.33306	-0.13823	5/147	Runx3,Cd80,Ido1,Lgals9,Sdc4
7_Member	GO Biological Processes	GO:0050670	regulation of lymphocyte proliferation	-2.21786	-0.10995	6/222	Cd80,Ido1,Lgals9,Stat5a,Sdc4,Tlr4
7_Member	GO Biological Processes	GO:0032944	regulation of mononuclear cell proliferation	-2.19939	-0.10699	6/224	Cd80,Ido1,Lgals9,Stat5a,Sdc4,Tlr4

7_Member	GO Biological Processes	GO:0042129	regulation of T cell proliferation	-2.09244	-0.05161	5/168	Cd80,Ido1,Lgals9,Stat5a,Sdc4
7_Member	KEGG Pathway	mmu04514	Cell adhesion molecules (CAMs)	-2.08192	-0.04773	5/169	Cd80,Mag,Nectin2,Sdc4,Lrrc4
7_Member	GO Biological Processes	GO:0070661	leukocyte proliferation	-2.03307	-0.02112	7/318	Cd80,Ido1,Lgals9,Kitl,Stat5a,Sdc4,Tlr4
7_Member	GO Biological Processes	GO:0042110	T cell activation	-2.01868	-0.01499	9/483	Runx3,Cd80,Ido1,Lgals9,Prkdc,Relb,Stat5a,Sdc4,Prex1
7_Member	GO Biological Processes	GO:0022408	negative regulation of cell-cell adhesion	-2.0104	-0.01499	5/176	Runx3,Cd80,Ido1,Lgals9,Sdc4
**8_Summary**	**GO Biological Processes**	**GO:1905952**	**regulation of lipid localization**	**-3.22367**	**-0.40333**	**6/140**	**Akt2,Ehd1,Lipg,Spp1,Thbs1,Hilpda,Cav2,Stat5a,Abca5,Pink1,Slc16a3,Pgap1**
8_Member	GO Biological Processes	GO:1905952	regulation of lipid localization	-3.22367	-0.40333	6/140	Akt2,Ehd1,Lipg,Spp1,Thbs1,Hilpda
8_Member	GO Biological Processes	GO:0010876	lipid localization	-2.84857	-0.28538	9/360	Akt2,Cav2,Ehd1,Lipg,Spp1,Stat5a,Thbs1,Hilpda,Abca5
8_Member	GO Biological Processes	GO:1905954	positive regulation of lipid localization	-2.42865	-0.17432	4/86	Ehd1,Lipg,Spp1,Hilpda
8_Member	GO Biological Processes	GO:0015850	organic hydroxy compound transport	-2.19024	-0.10365	6/225	Lipg,Spp1,Pink1,Slc16a3,Abca5,Pgap1
8_Member	GO Biological Processes	GO:0032368	regulation of lipid transport	-2.05304	-0.02821	4/110	Akt2,Lipg,Spp1,Thbs1
8_Member	GO Biological Processes	GO:0097006	regulation of plasma lipoprotein particle levels	-2.00952	-0.01499	3/60	Ehd1,Lipg,Abca5
9_Summary	GO Biological Processes	GO:0046718	viral entry into host cell	-3.20778	-0.40333	4/53	Cav2,Nectin2,Ifitm3,Ifitm2,Irf8,Lgals9,Gbp4,Timp1,Mapkbp1,Treml4,Cd207,Tlr13,Ifit1bl1,Lyz2,Tlr4,Hmgb2,Pnpt1
9_Member	GO Biological Processes	GO:0046718	viral entry into host cell	-3.20778	-0.40333	4/53	Cav2,Nectin2,Ifitm3,Ifitm2
9_Member	GO Biological Processes	GO:0043900	regulation of multi-organism process	-3.19315	-0.40333	10/390	Cav2,Irf8,Lgals9,Gbp4,Nectin2,Timp1,Mapkbp1,Ifitm3,Ifitm2,Treml4
9_Member	GO Biological Processes	GO:0009615	response to virus	-3.15712	-0.40333	8/260	Lgals9,Gbp4,Ifitm3,Ifitm2,Treml4,Cd207,Tlr13,Ifit1bl1
9_Member	GO Biological Processes	GO:0030260	entry into host cell	-3.1168	-0.40333	4/56	Cav2,Nectin2,Ifitm3,Ifitm2
9_Member	GO Biological Processes	GO:0044409	entry into host	-3.1168	-0.40333	4/56	Cav2,Nectin2,Ifitm3,Ifitm2
9_Member	GO Biological Processes	GO:0051806	entry into cell of other organism involved in symbiotic interaction	-3.1168	-0.40333	4/56	Cav2,Nectin2,Ifitm3,Ifitm2
9_Member	GO Biological Processes	GO:0051828	entry into other organism involved in symbiotic interaction	-3.1168	-0.40333	4/56	Cav2,Nectin2,Ifitm3,Ifitm2
9_Member	GO Biological Processes	GO:0046596	regulation of viral entry into host cell	-2.74424	-0.26272	3/33	Nectin2,Ifitm3,Ifitm2
9_Member	GO Biological Processes	GO:0098542	defense response to other organism	-2.53052	-0.20985	12/644	Irf8,Lgals9,Lyz2,Gbp4,Tlr4,Mapkbp1,Ifitm3,Ifitm2,Hmgb2,Treml4,Cd207,Ifit1bl1
9_Member	GO Biological Processes	GO:0034341	response to interferon-gamma	-2.50409	-0.19866	5/134	Irf8,Gbp4,Tlr4,Ifitm3,Ifitm2
9_Member	GO Biological Processes	GO:0002831	regulation of response to biotic stimulus	-2.43603	-0.17432	5/139	Lgals9,Gbp4,Nectin2,Mapkbp1,Treml4
9_Member	GO Biological Processes	GO:0051607	defense response to virus	-2.28421	-0.11629	6/215	Gbp4,Ifitm3,Ifitm2,Treml4,Cd207,Ifit1bl1
9_Member	GO Biological Processes	GO:0035456	response to interferon-beta	-2.25295	-0.112	3/49	Ifitm3,Pnpt1,Ifitm2
9_Member	Reactome Gene Sets	R-MMU-19893	Immunoregulatory interactions between a Lymphoid and a non-Lymphoid cell	-2.15181	-0.09156	4/103	Nectin2,Ifitm3,Ifitm2,Treml4
10_Summary	GO Biological Processes	GO:0032233	positive regulation of actin filament bundle assembly	-3.0036	-0.34972	4/60	Sdc4,Tgfbr1,Pdlim4,Synpo,Pls3,Rnd1,Gmfb,Prex1
10_Member	GO Biological Processes	GO:0032233	positive regulation of actin filament bundle assembly	-3.0036	-0.34972	4/60	Sdc4,Tgfbr1,Pdlim4,Synpo
10_Member	GO Biological Processes	GO:0051017	actin filament bundle assembly	-2.88058	-0.28884	6/163	Sdc4,Tgfbr1,Pdlim4,Pls3,Synpo,Rnd1
10_Member	GO Biological Processes	GO:0061572	actin filament bundle organization	-2.84013	-0.28538	6/166	Sdc4,Tgfbr1,Pdlim4,Pls3,Synpo,Rnd1

10_Member	GO Biological Processes	GO:0051492	regulation of stress fiber assembly	-2.46536	-0.19523	4/84	Sdc4,Tgfbr1,Pdlim4,Synpo
10_Member	GO Biological Processes	GO:0110020	regulation of actomyosin structure organization	-2.27487	-0.11629	4/95	Sdc4,Tgfbr1,Pdlim4,Synpo
10_Member	GO Biological Processes	GO:0051496	positive regulation of stress fiber assembly	-2.2284	-0.10995	3/50	Sdc4,Tgfbr1,Pdlim4
10_Member	GO Biological Processes	GO:0030038	contractile actin filament bundle assembly	-2.22735	-0.10995	4/98	Sdc4,Tgfbr1,Pdlim4,Synpo
10_Member	GO Biological Processes	GO:0043149	stress fiber assembly	-2.22735	-0.10995	4/98	Sdc4,Tgfbr1,Pdlim4,Synpo
10_Member	GO Biological Processes	GO:0032231	regulation of actin filament bundle assembly	-2.21188	-0.10995	4/99	Sdc4,Tgfbr1,Pdlim4,Synpo
10_Member	GO Biological Processes	GO:0007015	actin filament organization	-2.03521	-0.02112	8/397	Sdc4,Tgfbr1,Pdlim4,Gmfb,Pls3,Synpo,Rnd1,Prex1
11_Summary	GO Biological Processes	GO:0051345	positive regulation of hydrolase activity	-2.95034	-0.32089	11/495	Akt2,Camk2b,Cav2,Ppp1r15a,Pdcd2,Tbc1d1,Pink1,Hmgb2,St18,Prex1,Dennd1b
11_Member	GO Biological Processes	GO:0051345	positive regulation of hydrolase activity	-2.95034	-0.32089	11/495	Akt2,Camk2b,Cav2,Ppp1r15a,Pdcd2,Tbc1d1,Pink1,Hmgb2,St18,Prex1,Dennd1b
12_Summary	KEGG Pathway	mmu04066	HIF-1 signaling pathway	-2.93237	-0.32089	5/107	Akt2,Camk2b,Pfkl,Timp1,Tlr4,Nr4a1,Kitl,Spp1,Thbs1,Thbs4,Sgk3,Cav2,Sdc4,Cd80,Ldhb
12_Member	KEGG Pathway	mmu04066	HIF-1 signaling pathway	-2.93237	-0.32089	5/107	Akt2,Camk2b,Pfkl,Timp1,Tlr4
12_Member	KEGG Pathway	mmu04151	PI3K-Akt signaling pathway	-2.38208	-0.15934	8/346	Akt2,Nr4a1,Kitl,Spp1,Thbs1,Thbs4,Tlr4,Sgk3
12_Member	KEGG Pathway	mmu05205	Proteoglycans in cancer	-2.37369	-0.15934	6/206	Akt2,Camk2b,Cav2,Sdc4,Thbs1,Tlr4
12_Member	KEGG Pathway	mmu04620	Toll-like receptor signaling pathway	-2.21188	-0.10995	4/99	Akt2,Cd80,Spp1,Tlr4
12_Member	KEGG Pathway	mmu04922	Glucagon signaling pathway	-2.16657	-0.09156	4/102	Akt2,Camk2b,Ldhb,Pfkl
13_Summary	GO Biological Processes	GO:0090100	positive regulation of transmembrane receptor protein serine/threonine kinase signaling pathway	-2.93237	-0.32089	5/107	Gdf3,Got1,Lgals9,Tgfbr1,Thbs1,Cav2,Zfp451,Runx3,Cd59a,Ehd1,Nr4a1,Ier2,Flrt2,Jade2
13_Member	GO Biological Processes	GO:0090100	positive regulation of transmembrane receptor protein serine/threonine kinase signaling pathway	-2.93237	-0.32089	5/107	Gdf3,Got1,Lgals9,Tgfbr1,Thbs1
13_Member	GO Biological Processes	GO:0090092	regulation of transmembrane receptor protein serine/threonine kinase signaling pathway	-2.86321	-0.28538	7/224	Cav2,Gdf3,Got1,Lgals9,Tgfbr1,Thbs1,Zfp451
13_Member	GO Biological Processes	GO:0070848	response to growth factor	-2.7764	-0.27006	12/601	Cav2,Runx3,Cd59a,Ehd1,Gdf3,Got1,Nr4a1,Ier2,Tgfbr1,Thbs1,Zfp451,Flrt2
13_Member	GO Biological Processes	GO:0090288	negative regulation of cellular response to growth factor stimulus	-2.4765	-0.19553	5/136	Cav2,Cd59a,Gdf3,Thbs1,Zfp451
13_Member	GO Biological Processes	GO:0001937	negative regulation of endothelial cell proliferation	-2.47199	-0.19553	3/41	Cav2,Tgfbr1,Thbs1
13_Member	GO Biological Processes	GO:0071559	response to transforming growth factor beta	-2.41505	-0.17432	6/202	Cav2,Runx3,Got1,Tgfbr1,Thbs1,Zfp451
13_Member	GO Biological Processes	GO:0007178	transmembrane receptor protein serine/threonine kinase signaling pathway	-2.37461	-0.15934	8/347	Cav2,Gdf3,Got1,Lgals9,Tgfbr1,Thbs1,Jade2,Zfp451
13_Member	GO Biological Processes	GO:0017015	regulation of transforming growth factor beta receptor signaling pathway	-2.243	-0.112	4/97	Cav2,Got1,Thbs1,Zfp451
13_Member	GO Biological Processes	GO:1903844	regulation of cellular response to transforming growth factor beta stimulus	-2.21188	-0.10995	4/99	Cav2,Got1,Thbs1,Zfp451
13_Member	GO Biological Processes	GO:0007179	transforming growth factor beta receptor signaling pathway	-2.20211	-0.10699	5/158	Cav2,Got1,Tgfbr1,Thbs1,Zfp451
**14_Summary**	**KEGG Pathway**	**mmu00010**	**Glycolysis / Gluconeogenesis**	**-2.84873**	**-0.28538**	**4/66**	**Aldh2,Ldhb,Pfkl,Tpi1,Cbr2,Got1,Gstm2,Stat5a,Pink1,Hmgcs1**
14_Member	KEGG Pathway	mmu00010	Glycolysis / Gluconeogenesis	-2.84873	-0.28538	4/66	Aldh2,Ldhb,Pfkl,Tpi1

14_Member	GO Biological Processes	GO:0051186	cofactor metabolic process	-2.03522	-0.02112	9/480	Cbr2,Got1,Gstm2,Ldhb,Pfkl,Stat5a,Tpi1,Pink1,Hmgcs1
15_Summary	GO Biological Processes	GO:0006897	endocytosis	-2.8037	-0.27006	13/679	Cav2,Ehd1,Irf8,Napb,Rab4a,Thbs1,Calcrl,Ifitm3,Sh3bp4,Treml4,Cd300lf,Dennd1b,Xkr8,Relb
15_Member	GO Biological Processes	GO:0006897	endocytosis	-2.8037	-0.27006	13/679	Cav2,Ehd1,Irf8,Napb,Rab4a,Thbs1,Calcrl,Ifitm3,Sh3bp4,Treml4,Cd300lf,Dennd1b,Xkr8
15_Member	GO Biological Processes	GO:0043277	apoptotic cell clearance	-2.53419	-0.20985	3/39	Thbs1,Cd300lf,Xkr8
15_Member	GO Biological Processes	GO:0098657	import into cell	-2.34626	-0.14271	13/768	Cav2,Ehd1,Irf8,Napb,Rab4a,Thbs1,Calcrl,Ifitm3,Sh3bp4,Treml4,Cd300lf,Dennd1b,Xkr8
15_Member	GO Biological Processes	GO:0019882	antigen processing and presentation	-2.1228	-0.07925	4/105	Rab4a,Relb,Thbs1,Treml4
16_Summary	GO Biological Processes	GO:0018105	peptidyl-serine phosphorylation	-2.77874	-0.27006	8/298	Akt2,Camk2b,Ppp1r15a,Prkdc,Tgfbr1,Rps6ka4,Pink1,Sgk3,Nek2,Sdc4,Pdlim4,Gmfb,Synpo,Nusap1,Impact,Pim3
16_Member	GO Biological Processes	GO:0018105	peptidyl-serine phosphorylation	-2.77874	-0.27006	8/298	Akt2,Camk2b,Ppp1r15a,Prkdc,Tgfbr1,Rps6ka4,Pink1,Sgk3
16_Member	GO Biological Processes	GO:0018209	peptidyl-serine modification	-2.59546	-0.22727	8/319	Akt2,Camk2b,Ppp1r15a,Prkdc,Tgfbr1,Rps6ka4,Pink1,Sgk3
16_Member	GO Biological Processes	GO:0010638	positive regulation of organelle organization	-2.48559	-0.19819	11/568	Akt2,Camk2b,Nek2,Sdc4,Tgfbr1,Pdlim4,Rps6ka4,Gmfb,Pink1,Synpo,Nusap1
16_Member	GO Biological Processes	GO:0046777	protein autophosphorylation	-2.04225	-0.02112	6/242	Camk2b,Impact,Nek2,Tgfbr1,Pink1,Pim3
17_Summary	GO Biological Processes	GO:1903959	regulation of anion transmembrane transport	-2.66986	-0.22727	3/35	Akt2,Thbs1,Tcaf1,Pink1,Zfand2b,Zdhhc2,Pnpt1,Gadd45gip1
17_Member	GO Biological Processes	GO:1903959	regulation of anion transmembrane transport	-2.66986	-0.22727	3/35	Akt2,Thbs1,Tcaf1
17_Member	GO Biological Processes	GO:1903533	regulation of protein targeting	-2.1354	-0.08358	3/54	Akt2,Pink1,Tcaf1
17_Member	GO Biological Processes	GO:0006612	protein targeting to membrane	-2.1228	-0.07925	4/105	Akt2,Zfand2b,Zdhhc2,Tcaf1
17_Member	GO Biological Processes	GO:0043467	regulation of generation of precursor metabolites and energy	-2.1228	-0.07925	4/105	Akt2,Pink1,Pnpt1,Gadd45gip1
18_Summary	GO Biological Processes	GO:0010035	response to inorganic substance	-2.65833	-0.22727	10/460	Camk2b,Nr4a1,Impact,Junb,Jund,Mt1,Thbs1,Slc40a1,Pink1,Kdm6b
18_Member	GO Biological Processes	GO:0010035	response to inorganic substance	-2.65833	-0.22727	10/460	Camk2b,Nr4a1,Impact,Junb,Jund,Mt1,Thbs1,Slc40a1,Pink1,Kdm6b
19_Summary	Reactome Gene Sets	R-MMU-20400	COPII-mediated vesicle transport	-2.64394	-0.22727	4/75	Bet1,Cd59a,Napb,Ankrd28,Arf5,Man1a2,Akt2,Kif11,Rab4a,Tbc1d1,Dennd1b
19_Member	Reactome Gene Sets	R-MMU-20400	COPII-mediated vesicle transport	-2.64394	-0.22727	4/75	Bet1,Cd59a,Napb,Ankrd28
19_Member	Reactome Gene Sets	R-MMU-94802	Transport to the Golgi and subsequent modification	-2.61476	-0.22727	6/184	Arf5,Bet1,Cd59a,Man1a2,Napb,Ankrd28
19_Member	Reactome Gene Sets	R-MMU-19999	Membrane Trafficking	-2.48559	-0.19819	11/568	Akt2,Arf5,Bet1,Cd59a,Kif11,Man1a2,Napb,Rab4a,Tbc1d1,Ankrd28,Dennd1b
19_Member	Reactome Gene Sets	R-MMU-56536	Vesicle-mediated transport	-2.28201	-0.11629	11/605	Akt2,Arf5,Bet1,Cd59a,Kif11,Man1a2,Napb,Rab4a,Tbc1d1,Ankrd28,Dennd1b
19_Member	Reactome Gene Sets	R-MMU-19997	ER to Golgi Anterograde Transport	-2.2484	-0.112	5/154	Arf5,Bet1,Cd59a,Napb,Ankrd28
19_Member	Reactome Gene Sets	R-MMU-68078	COPI-mediated anterograde transport	-2.243	-0.112	4/97	Arf5,Bet1,Cd59a,Napb
20_Summary	**GO Biological Processes**	**GO:0032479**	**regulation of type I interferon production**	**-2.62292**	**-0.22727**	**4/76**	**Gbp4,Relb,Tlr4,Hmgb2**
20_Member	GO Biological Processes	GO:0032479	regulation of type I interferon production	-2.62292	-0.22727	4/76	Gbp4,Relb,Tlr4,Hmgb2
20_Member	GO Biological Processes	GO:0032606	type I interferon production	-2.5617	-0.20985	4/79	Gbp4,Relb,Tlr4,Hmgb2
20_Member	GO Biological Processes	GO:0032648	regulation of interferon-beta production	-2.2284	-0.10995	3/50	Relb,Tlr4,Hmgb2
20_Member	GO Biological Processes	GO:0032608	interferon-beta production	-2.1809	-0.1003	3/52	Relb,Tlr4,Hmgb2

Supplemental table 5- Lesion scores distribution in aortic root leaflets- 16 weeks WD

Lesion type	Frequency	
	***Apoe*$^{-/-}$**	***Apoe*$^{-/-}$/*Treml4*$^{-/-}$**
III	1/30 (3.3%)	8/39 (20.5%)
IV	12/30 (40%)	17/39 (43.6%)
V	10/30 (33.3%)	4/39 (10.3%)
VI	7/30 (23.3%)	10/39 (25.6%)

Supplemental table 6- DEG *Apoe*$^{-/-}$ /*Treml4*$^{-/-}$ oxLDL60 vs. *Apoe*$^{-/-}$ oxLDL60

Cluster 1

gene	Fold change	p-value
Sox9	-19.0257	0.00956002
Tmem240	-12.0326	0.0243995
Ect2l	-11.5275	0.00494744
Gm13278	-10.091	0.0302291
Vmn1r32	-9.88038	0.0315939
Fam131b	-9.65811	0.0107308
Ccn5	-8.94419	0.026509
F2rl1	-8.80283	0.0388109
Tenm4	-8.68859	0.0210419
Slc16a8	-8.33382	0.0454222
Hoxc5	-8.21338	0.00669559
Scrn1	-7.73917	0.022394
Tcaf2	-7.65591	0.0203842
Pcdhga3	-7.55801	0.0273524
Henmt1	-7.30573	0.0269455
Gm29686	-7.30573	0.0466291
Calca	-6.3249	0.0273803
Podn	-5.78885	0.0329171
Pde6a	-5.75288	0.00988554
Ush1c	-5.42889	0.0448806
Stfa2l1	-5.21894	0.00120131
Tchh	-5.20229	0.0355136
Nnmt	-5.13899	0.0195096
Slc44a4	-5.13647	0.0346067
Fzd8	-4.9397	0.0291514
Atp1a2	-4.78641	0.0284284
Sp6	-4.72203	0.0488831
Sfrp2	-4.60666	0.0164472
Tmeff2	-4.58521	0.0460576
Gucy1a2	-4.54504	0.0452236
Lrrc74b	-4.35931	0.0235242
Gm48908	-4.20584	0.0287744
Nek11	-4.18277	0.0045785
Mrgpra6	-4.13774	0.0391213
Myl4	-4.07189	0.0191682

Aldh3a1	-4.02915	0.0375738
Rdh9	-3.95001	0.00206341
Hic1	-3.89035	0.0282618
4930432J09Rik	-3.69485	0.0440756
Thsd4	-3.53802	0.0475236
Spdye4b	-3.39753	0.032091
Abhd16b	-3.29228	0.0195396
Dkk2	-3.20806	0.0193172
Ncmap	-2.9419	0.0028391
4930470G03Rik	-2.91923	0.0262223
4430402I18Rik	-2.79011	0.0283828
4930451G09Rik	-2.6834	0.0318638
Lrrc32	-2.66747	0.0488204
Asb5	-2.66142	0.0230051
Tubg2	-2.64364	0.00805588
Cd209f	-2.64056	0.0390906
Rpl39l	-2.61537	3.01E-05
Eps8l1	-2.60993	0.00301783
Pdk4	-2.57579	0.0260786
Itm2a	-2.52803	0.0287467
Slc6a17	-2.32674	0.00191438
Casp12	-2.30844	0.0257566
Rtn4rl1	-2.28854	0.00482121
Slc16a12	-2.25906	0.0316896
1700028J19Rik	-2.25418	0.00772578
Gm14461	-2.24438	0.0227622
Ccdc148	-2.16815	0.0487256
Nsg1	-2.1005	0.0497046
Exoc3l	-2.08899	0.037493
Slit1	-2.06383	0.00409161
Fam174b	-2.02341	0.0251629
Spa17	-2.01358	8.18E-05
Xkrx	-2.00202	0.0192908
Iqcd	-1.94264	0.0344983
Gm5547	-1.85774	0.0459374
Ramp3	-1.85698	0.0352808
Armc2	-1.82141	0.031015
Epdr1	-1.81922	0.0022696
Fgd1	-1.81775	0.0358961
Man2c1os	-1.8077	0.00894556
Stkld1	-1.79537	0.00845022

Zfp385c	-1.77164	0.00200197
Glis2	-1.74988	0.00962954
Fam170b	-1.74041	0.0406194
Gpr68	-1.73817	3.60E-05
Slc18b1	-1.72793	0.036967
Dclk2	-1.7211	0.0460778
C8g	-1.6899	0.0263555
Ednrb	-1.68952	0.00184109
Nxpe5	-1.68619	0.0479868
Entpd4	-1.68261	1.30E-06
Smco3	-1.67836	0.0408975
Epb41l3	-1.67077	0.0297459
Stac2	-1.67071	0.0287905
Gpd1	-1.65492	0.00333926
Ctsg	-1.64585	0.031862
Slc39a12	-1.6418	0.0453547
Rhbdf1	-1.64051	0.0276474
Zfp2	-1.63378	0.00387471
Mmp9	-1.6244	0.0474773
Arhgef5	-1.62189	0.0131353
Triqk	-1.61653	0.0224577
Gpx7	-1.60341	0.0457074
Gm15972	-1.58775	0.0443164
Entpd4b	-1.5828	1.30E-06
Gpx3	-1.56247	0.0201724
Ubr2	-1.55401	0.00342941
Agmo	-1.54497	0.0328485
Fbxo32	-1.54	0.0172469
2610035D17Rik	-1.5376	0.0222783
Tmem151a	-1.51348	0.0454698
Rnase4	-1.50752	1.88E-05

Cluster 2

gene	Fold change	p-value
Tnfrsf10b	1.50405	0.0367875
AI507597	1.50648	0.00840288
Igflr1	1.51741	0.00245009
Zfp469	1.51852	0.016779
Fbxw10	1.52091	0.0447728

Tmem25	1.52908	0.0219922
E330020D12Rik	1.53261	0.0275611
Xk	1.53327	0.00220164
2010300C02Rik	1.53717	8.92E-05
H2-Q6	1.54091	0.0301836
Efhc1	1.54631	0.0221659
Gdap1l1	1.56871	0.0153938
Gnat3	1.5723	0.022864
Plekhh2	1.58766	0.0219467
Nrip3	1.59705	0.00862041
Gpr35	1.59851	0.0297579
Olfml3	1.60379	5.88E-05
Mast4	1.61976	0.0279317
Osbp2	1.62488	0.0373613
Trp53cor1	1.62586	0.0123841
Camsap3	1.63069	0.00181517
Plekhn1	1.63138	0.00143611
Gm14853	1.63348	0.0277613
Malat1	1.636	0.00954316
Tmem267	1.65246	0.0337494
H2-Q9	1.65399	0.0394903
Efr3b	1.66126	0.018491
1700003E16Rik	1.66212	0.0405339
Dmc1	1.66986	0.0291039
4930565N06Rik	1.67606	0.0496676
Ppm1e	1.69011	0.0150692
Perm1	1.6949	1.56E-05
Nptx1	1.6966	0.00964485
Fads6	1.70034	0.00807159
Ccdc157	1.70188	0.000663196
Adra1a	1.7059	0.0367536
4931406H21Rik	1.70653	0.0156989
Gm36210	1.71543	0.0390999
Slc9a4	1.71596	0.00790945
Ceacam16	1.71802	0.0183032
B230319C09Rik	1.71892	0.0045253
Nnt	1.72399	1.82E-08
Pabpc1l	1.72815	0.0424395
AI450353	1.73213	0.00741307
Fhod3	1.74076	3.67E-05
Terc	1.74266	0.00153114

Adam4	1.7511	0.0398783
Mid1	1.76798	0.000735229
Kcnq1ot1	1.7714	0.00407486
Pdzd2	1.77537	0.0329887
Lrrc66	1.80141	0.00963074
1810021B22Rik	1.81136	0.0402051
Capn3	1.83289	0.0345115
Lrrc39	1.83384	0.0206105
Soat2	1.84274	0.0466075
Igsf11	1.85241	1.96E-06
Nnat	1.85907	0.0404139
Reep1	1.86201	0.0203235
Plin4	1.88574	0.0444597
Rhbdl3	1.9005	0.047935
9430060I03Rik	1.90668	0.0373071
Pnma1	1.91099	0.00189594
Insyn2b	1.91104	0.0281411
Dnd1	1.95059	0.0018584
Lrp8os2	1.97099	0.00333875
Fam83g	1.97282	0.0260282
Ttn	1.98755	0.0226203
Chic1	2.0133	0.040843
Pglyrp1	2.05455	0.00571144
Uchl4	2.10913	0.0337017
4632428C04Rik	2.11197	0.0109858
4930487H11Rik	2.13	0.0016807
Gm4532	2.13943	0.0253055
Xntrpc	2.14412	0.0291438
Slc36a2	2.18022	8.15E-05
Wfikkn2	2.1895	0.011812
Klkb1	2.19702	0.00655187
Tcea2	2.24736	0.00201476
Flrt1	2.26512	0.0184842
Ppp1r16b	2.30717	0.0108949
Saxo2	2.31345	0.0436679
Xlr4a	2.31366	0.0228822
A530088E08Rik	2.32513	0.0425724
Gm11166	2.35552	0.0340776
Cited4	2.38233	0.0448842
Carmil2	2.3904	0.0197007
Slc1a2	2.3965	0.016804

Slc3a1	2.43419	0.0117796
Slc2a5	2.44579	0.00756176
Irf6	2.47681	0.0257976
Nr4a3	2.49307	0.014655
Gm7854	2.49605	0.0498046
Fam167a	2.52594	0.0379082
Lhx2	2.53183	0.0225248
Hnf1a	2.53264	0.00515692
5430402O13Rik	2.5407	0.0107364
Cgn	2.58201	0.012836
Spred3	2.59915	0.000615074
Olfr111	2.61561	0.00558798
Hspa1a	2.61631	0.0230129
Kcnj13	2.63553	0.023351
Gm38397	2.65097	0.0020513
Myo1a	2.67641	0.00822139
Hunk	2.67681	0.0300051
Gm15713	2.71229	0.0484685
Gm13031	2.74809	0.0300936
Gm7334	2.77696	0.0137552
4930478L05Rik	2.80191	0.0130466
9930111H07Rik	2.82357	0.00927893
Bbof1	2.87921	0.00665063
Sdk2	2.92192	0.0307977
Gm15446	2.95134	0.0484946
Serpind1	2.95541	0.0178172
Ppp1r1a	3.06114	0.00380936
Cyb5r2	3.0851	0.00173736
Map9	3.11835	0.0325554
Tmprss9	3.14198	0.00328464
Gm21859	3.1429	0.0247678
Iglon5	3.15035	0.0180769
Diras1	3.1549	0.0326131
Slc16a4	3.17588	0.00584256
6030443J06Rik	3.22849	0.0229823
Arhgap27os3	3.25522	0.0295234
Mmp28	3.26203	0.00741331
Aox4	3.29142	0.0119956
Efcab7	3.29277	3.87E-05
Gm11517	3.30671	0.0399405
Lrrn3	3.35639	0.0271846

4933411K16Rik	3.41873	0.0473281
Zfp965	3.55844	0.0021056
Gm4832	3.57939	0.0425494
Npm2	3.57939	0.0366202
Sv2c	3.57939	0.0437477
Lancl3	3.65853	0.000985426
Myo5b	3.74208	0.0379314
Tgfbr3l	3.79607	0.0239828
Ddx43	3.81645	0.00688051
Slc2a12	3.92608	0.0232287
Hoxa2	3.97369	0.00367299
Oas1h	3.97605	0.017734
Plin5	4.00389	0.0469322
Tmem198	4.01535	0.00875068
Gm16897	4.0557	0.0224556
G530011O06Rik	4.05916	8.02E-06
Gm12216	4.13755	0.0233596
Slc46a2	4.21917	0.0170287
Erdr1	4.34903	0.000672466
Glyat	4.40605	0.0282233
Gm7429	4.54632	0.0165917
Wnt3	4.62649	0.0458572
Rbakdn	4.75658	0.0135087
Ak7	4.78993	0.0215845
Zfp831	4.78993	0.0273582
Gm21119	5.25139	0.00843373
4833415N18Rik	5.51796	0.0339885
Ndufa4l2	5.52876	0.0345875
Nkpd1	5.75276	0.0122426
Ccdc169	5.99982	0.00167315
Scn8a	6.0721	0.00443709
Cxadr	6.15169	0.00526935
Enox1	6.30689	0.043153
9030404E10Rik	6.47462	4.82E-05
Gm7819	6.50716	0.00375066
D930015M05Rik	6.6782	0.0110003
Slc40a1	6.76466	2.51E-10
Prph	7.08366	0.0311977
Spatc1	7.3513	0.0361568
Scnn1b	7.36159	0.000729638
Pip5kl1	7.39358	0.00582486

Usp43	8.10693	0.0319459
Lrrc2	8.28774	0.0398942
Pnma2	8.29641	0.0274442
Gm4651	8.47072	0.0113025
Amhr2	8.50898	0.0146087
Cda	8.61946	0.00126154
Dtx1	8.72263	0.00668185
Omd	9.39605	0.00875667
Slc23a3	9.60922	0.00145792
Snord16a	9.69513	0.0451337
43711	10.0696	0.00185106
Unc93a2	10.1708	9.44E-07
Ankrd22	10.4188	0.0395944
4930447M23Rik	11.3553	0.021801
Mir703	11.7884	0.00426843
Oxct2a	12.9337	0.00980232
Disp2	13.3811	0.013304
